# Visible light-mediated difluoroalkylation of electron-deficient alkenes

**DOI:** 10.3762/bjoc.14.139

**Published:** 2018-07-02

**Authors:** Vyacheslav I Supranovich, Vitalij V Levin, Marina I Struchkova, Jinbo Hu, Alexander D Dilman

**Affiliations:** 1 N. D. Zelinsky Institute of Organic Chemistry, 119991 Moscow, Leninsky prosp. 47, Russian Federation; 2 Key Laboratory of Organofluorine Chemistry, Center for Excellence in Molecular Synthesis, Shanghai Institute of Organic Chemistry, University of Chinese Academy of Sciences, Chinese Academy of Sciences, 345 Ling-Ling Road, Shanghai 200032, China

**Keywords:** difluoroalkylation, organofluorine compounds, radical addition, visible light

## Abstract

A method for the reductive difluoroalkylation of electron-deficient alkenes using 1,1-difluorinated iodides mediated by irradiation with blue light is described. The reaction involves radical addition of 1,1-difluorinated radicals at the double bond followed by hydrogen atom transfer from sodium cyanoborohydride.

## Introduction

Applications of organofluorine compounds in medicinal chemistry and related fields [1–2] have stimulated intensive developments of methods for their synthesis [3–4]. Though the major emphasis has long been placed on trifluoromethylated molecules, compounds bearing the difluoromethylene fragment have attracted increasing attention in recent years [5–8]. Thus, besides the bioisosteric relationship with ethereal oxygen [9–11], the CF_2_-unit can influence the reactivity of adjacent functional groups [12–13] and can affect conformational properties of chain and cyclic molecules [14–15].

**Scheme 1:**
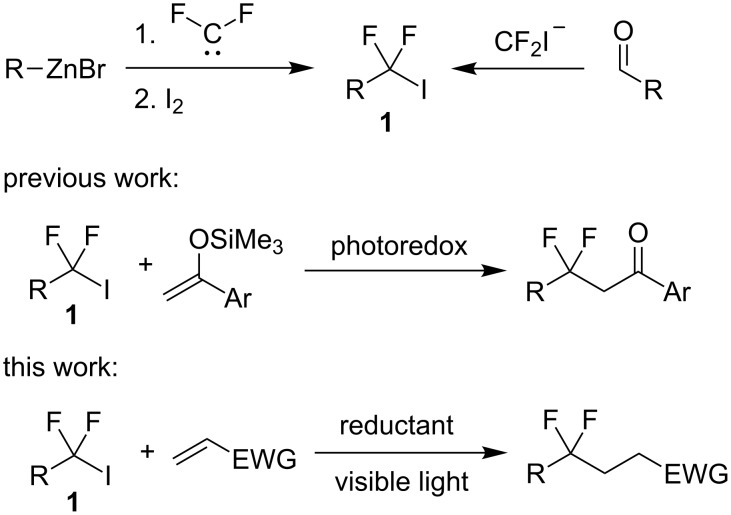
Fluoroalkylation of alkenes.

Radical fluoroalkylation of double bonds is a well-established approach for the synthesis of organofluorine compounds [16–24]. While perfluorinated alkyl iodides and bromides are typically employed, reactions with *gem*-difluorinated iodides are less elaborated, which is primarily associated with availability issues [25–29]. Recently, we developed a general protocol for the synthesis of iodides **1** from organozinc reagents and a source of difluorocarbene [30–32] (Scheme 1). Moreover, it was shown that compounds bearing the CF_2_I group can be obtained from carbonyl compounds and equivalents of the iododifluoromethyl carbanion [33–36]. We also demonstrated that iodides **1** can alkylate silyl enol ethers [37] under photoredox conditions [38–40]. However, the latter protocol is inapplicable to the addition to electron-deficient alkenes since a radical resulting from the addition step cannot be oxidized by photocatalysts. Herein we report a convenient method for performing hydroperfluoroalkylation of electron-deficient alkenes employing iodides **1** mediated by visible light. The reaction proceeds without the use of a photosensitizer or a photocatalyst.

Generation of fluorinated alkyl radicals is typically realized either by single-electron reduction or by radical abstraction of iodine [41–43]. Furthermore, the carbon–iodine bond is prone to homolytic cleavage under UV irradiation [44]. In this regard, we were inspired by the work of Ryu describing the use of energetic UV light (xenon arc lamp) in combination with sodium cyanoborohydride to trigger the reaction of non-fluorinated alkyl iodides [45–47]. Subsequently, we showed that silyldifluoromethyl iodide can generate the corresponding *gem*-difluorinated radical in combination with an N-heterocyclic carbene/borane complex and 400 nm light [48].

## Results and Discussion

Iodide **1a** was selected as a model substrate and its reaction with *tert*-butyl acrylate (**2a**) was evaluated in the presence of various boron hydrides (Table 1). The reaction was performed in methanol with irradiation by a strip of blue light emitting diodes. The use of sodium cyanoborohydride provided a moderate yield of product **3a** and about 60% conversion of **1a** (Table 1, entry 1). The addition of pyridine notably increased the yield, presumably, by scavenging boron species with a B–I bond formed in the reaction (Table 1, entry 2). However, some byproducts were observed in amounts of up to 10%, which were tentatively identified as oligomers arising from multiple addition of *tert*-butyl acrylate to the propagating radical. Fortunately, increase of the amount of borohydride to 2 equiv suppressed the formation of oligomers, and addition product **3a** was isolated in 83% yield (Table 1, entry 6). *N*,*N*'-Dimethylimidazolidene borane complex (NHC·BH_3_) was also efficient (Table 1, entry 5), but because of availability and cost issues, sodium cyanoborohydride was used for further studies. We also evaluated the photoredox-mediated hydrofluoroalkylation process using *fac*-Ir(ppy)_3_ as a photocatalyst in combination with a suitable donor of hydrogen atom. With triethylamine, no expected product was observed. At the same time, with Hantzsch ester (3 equiv), which can serve as a single-electron reductant and as a source of hydrogen [16], 54% of product **3a** was formed. However, difficulties in removing the pyridine byproduct formed from Hantzsch ester and the use of a precious metal photocatalyst make this protocol less practical compared to that with sodium cyanoborohydride.

**Table 1:** Optimization studies.

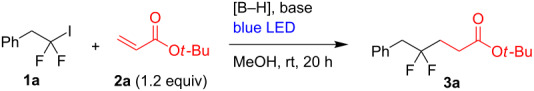			

entry	hydride (equiv)	base (equiv)	yield, %^a^

1	NaBH_3_CN (1.5)	–	46
2	NaBH_3_CN (1.5)	pyridine (1.5)	77^b^
3	Py-BH_3_ (1.5)	pyridine (1.5)	78^b^
4	Py-BH_3_ (2.0)	pyridine (2.0)	80^b^
5	NHC·BH_3_ (1.5)	pyridine (1.5)	76^c^
6	NaBH_3_CN (2.0)	pyridine (1.5)	83^c^
7	NaBH_3_CN (2.0)	NaOAc (1.5)	74^b^

^a^Determined by ^19^F NMR with internal standard. ^b^Oligomeric byproducts (5–11%) were observed. ^c^Isolated yield.

**Figure 1:**
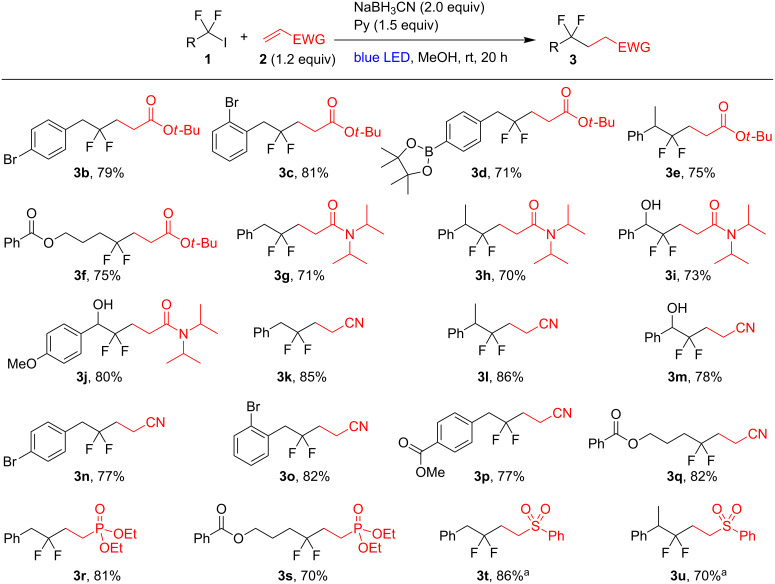
Difluoroalkylation of alkenes. Isolated yields are shown. ^a^2 equiv of the alkene were used.

Under the optimized conditions a series of *gem*-difluorinated iodides **1** were coupled with alkenes **2** (Figure 1). Esters and amides of acrylic acid, acrylonitrile, and vinyl phosphonate used in a small excess (1.2 equiv) were successfully fluoroalkylated. For vinyl(phenyl)sulfone, significant amounts of hydrodeiodination product R_f_H was formed, likely because of the decreased reactivity of the sulfone double bond toward fluorinated radicals. In this case, two equivalents of the alkene have to be used to achieve good yields of products **3t**,**u**. It should also be pointed out that the reaction tolerates aromatic bromide substituents (products **3b**,**c**,**n**,**o**), a boryl fragment (product **3d**) and unprotected hydroxy groups (products **3i**,**j**,**m**). Electron-rich and neutral alkenes were ineffective in this reaction. Indeed, 1-phenyl-(1-trimethylsilyloxy)ethylene and 4-phenylbut-1-ene gave no hydrofluoroalkylation products when combined with iodide **1a** under standard conditions.

**Scheme 2:**
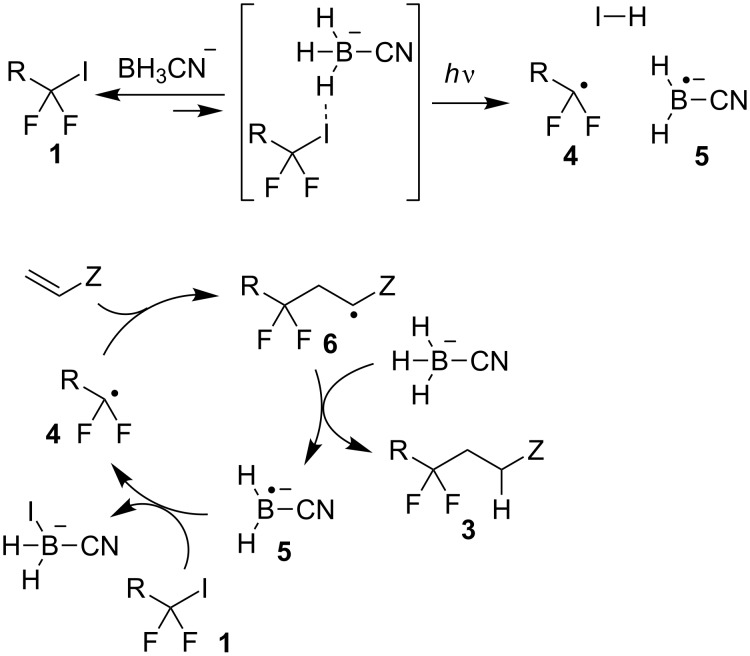
Proposed mechanism.

The proposed mechanism is shown in Scheme 2. First, difluorinated iodide **1** interacts with boron hydride to form a small equilibrium concentration of a halogen-bonded complex [49–50]. This complex is activated by light to effect homolytic cleavage of the carbon–iodine bond with the formation of radical **4** and boron-centered radical anion **5**. After the initiation event, the reaction proceeds via a chain mechanism. Thus, radical addition at the double bond gives radical **6**, which abstracts a hydrogen atom from cyanoborohydride to generate boryl radical anion **5**. The latter species can readily abstract the iodine atom from starting iodide **1** which results in the formation of radical **4** [51–52].

## Conclusion

In summary, a convenient method for the synthesis of *gem*-difluorinated compounds via hydrofluoroalkylation of electron-deficient alkenes is described. Readily available boron cyanohydride is used as a source of hydrogen and as a trigger for the generation of free radicals mediated by visible light.

## Supporting Information

File 1 Full experimental details, compound characterization, and copies of NMR spectra.
