# Supplementary material for: Visible light-mediated difluoroalkylation of electron-deficient alkenes
Source: Beilstein J Org Chem. 2018 Jul 2;14:1637–41. doi: 10.3762/bjoc.14.139 (PMC6036985; doi:10.3762/bjoc.14.139)
Supplement: File 1 — Full experimental details, compound characterization, and copies of NMR spectra. [file Beilstein_J_Org_Chem-14-1637-s001.pdf]

Supporting Information

for

Visible light-mediated difluoroalkylation of electron-deficient alkenes

Vyacheslav I. Supranovich[1], Vitalij V. Levin[1], Marina I. Struchkova[1], Jinbo Hu[2] and Alexander D. Dilman*[1]

Address: [1]N. D. Zelinsky Institute of Organic Chemistry, 119991 Moscow, Leninsky prosp. 47, Russian Federation and [2]Key Laboratory of Organofluorine Chemistry, Center for Excellence in Molecular Synthesis, Shanghai Institute of Organic Chemistry, University of Chinese Academy of Sciences, Chinese Academy of Sciences, 345 Ling-Ling Road, Shanghai 200032, China

Email: Alexander D. Dilman - adil25@mail.ru

*Corresponding author

Full experimental details, compound characterization, and copies of NMR spectra

Content

	Page
General Methods	S2
Synthesis of compounds **3a–u**	S2
Compounds **3a–u**	S2–S9
NMR spectra	S10–S74

General methods: Column chromatography was carried out employing silica gel (230–400 mesh). Precoated silica gel plates F-254 were used for thin-layer analytical chromatography visualizing with UV and/or acidic aq $KMnO_4$ solution. High resolution mass spectra (HRMS) were measured using electrospray ionization (ESI) and time-of-flight (TOF) mass analyzer. The measurements were done in a positive ion mode (interface capillary voltage –4500 V) or in a negative ion mode (3200 V); mass range from *m/z* 50 to *m/z* 3000. For irradiation, a strip of light emitting diodes (2835-120LED 1M-Blue, 12V) was used.

Reagents. The following starting compounds were prepared according to literature procedures: (2,2-difluoro-2-iodoethyl)benzene (**1a**),[1] 1-bromo-2-(2,2-difluoro-2-iodoethyl)benzene,[1] 4-(4,4,5,5-tetramethyl-1,3,2-dioxaborolan-2-yl)-2-(2,2-difluoro-2-iodoethyl)benzene,[1] (2,2-difluoro-2-iodo-1-methylethyl)benzene,[1] 4,4-difluoro-4-iodobutyl benzoate,[1] 2,2-difluoro-2-iodo-1-phenylethanol,[2] 2,2-difluoro-2-iodo-1-(4-methoxyphenyl)ethanol,[2] methyl 4-(2,2-difluoro-1-hydroxy-2-iodoethyl)-benzoate.[1]

Difluoroalkylation of alkenes (General procedure).

The tube containing a stirring bar and sodium cyanoborohydride (63 mg, 1.0 mmol) was evacuated and filled with argon. Then, methanol (1.0 mL), iodide **1** (0.50 mmol), alkene **2** [for **3a–s**, 0.60 mmol; for **3t,u**, 1.00 mmol], and pyridine (59 mg, 0.75 mmol) were added successively. The tube was tightly closed with a screw cap and the reaction mixture was irradiated by a strip of blue LED with cooling maintaining the reaction temperature around 23–25 °C. For the work-up, the reaction mixture was poured into brine (NaCl, 5 mL) and water (5 mL) mixture, and the product was extracted with ethyl acetate (3 × 4 mL). The combined organic phases were dried with Na_2SO_4 and concentrated under vacuum, and the residue was purified by column chromatography.

***tert*-Butyl 4,4-difluoro-5-phenylpentanoate (3a).**

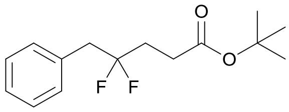

Yield 112 mg (83%). Colorless oil. Chromatography: hexanes/EtOAc, 10/1. R_f 0.29 (hexanes/EtOAc, 10/1).

^{1}H NMR (300 MHz, $CDCl_3$) δ: 7.38–7.27 (m, 5H), 3.17 (t, *J* = 16.0 Hz, 2H), 2.47 (t, *J* = 7.9 Hz, 2H), 2.00–2.03 (m, 2H), 1.46 (s, 9H).

^{13}C{^{1}H} NMR (75.5 MHz, $CDCl_3$) δ: 171.7, 133.2 (t, *J* = 4.8 Hz), 130.4, 128.6, 127.5, 123.5 (t, *J* = 242.6 Hz), 80.8, 43.5 (t, *J* = 25.9 Hz), 31.3 (t, *J* = 25.2 Hz), 28.3 (t, *J* = 4.5 Hz), 28.1.

^{19}F NMR (282 MHz, $CDCl_3$) δ: –99.1 (quint, *J* = 16.0 Hz).

HRMS (ESI): calcd for $C_{15}H_{21}F_2O_2$ (M+H) 271.1504; found 271.1500.

[1] Levin, V. V.; Zemtsov, A. A.; Struchkova, M. I.; Dilman, A. D. *Org. Lett.* **2013**, *15*, 917–919.

[2] Levin, V. V.; Smirnov, V. O.; Struchkova, M. I.; Dilman, A. D. *J. Org. Chem.* **2015**, *80*, 9349–9353.

tert-Butyl 5-(4-bromophenyl)-4,4-difluoropentanoate (3b).

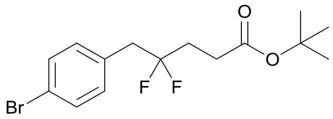

Yield 138 mg (79%). Colorless oil. Chromatography: hexanes/EtOAc, 10/1. R_f 0.36 (hexanes/EtOAc, 10/1).

^{1}H NMR (300 MHz, $CDCl_3$) δ: 7.44 (d, *J* = 8.3 Hz, 2H), 7.13 (d, *J* = 8.3 Hz, 2H), 3.09 (t, *J* = 16.0 Hz, 2H), 2.43 (t, *J* = 7.8 Hz, 2H), 2.16–1.99 (m, 2H), 1.43 (s, 9H).

^{13}C{^{1}H} NMR (75.5 MHz, $CDCl_3$) δ: 171.5, 132.1, 131.7, 123.1 (t, *J* = 242.9 Hz), 121.6, 80.0, 42.8 (t, *J* = 26.2 Hz), 31.3 (t, *J* = 25.1 Hz), 28.2 (t, *J* = 4.4 Hz), 28.1.

^{19}F NMR (282 MHz, $CDCl_3$) δ: –99.4 (quint, *J* = 16.0 Hz).

HRMS (ESI): calcd for $C_{15}H_{19}BrF_2O_2Na$ (M+Na) 371.0429, 373.0409; found 371.0420, 373.0404.

tert-Butyl 5-(2-bromophenyl)-4,4-difluoropentanoate (3c).

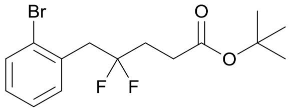

Yield 142 mg (81%). Colorless oil. Chromatography: hexanes/EtOAc, 10/1. R_f 0.39 (hexanes/EtOAc, 10/1).

^{1}H NMR (300 MHz, $CDCl_3$) δ: 7.75 (dd, *J* = 8.0, 1.2 Hz, 1H), 7.37 (d, *J* = 7.8 Hz, 1H), 7.27 (td, *J* = 7.5, 1.3 Hz, 1H), 7.13 (td, *J* = 7.7, 1.8 Hz, 1H), 3.40 (t, *J* = 16.0 Hz, 2H), 2.47 (t, *J* = 7.8 Hz, 2H), 2.27–2.10 (m, 2H), 1.44 (s, 9H).

^{13}C{^{1}H} NMR (75.5 MHz, $CDCl_3$) δ: 171.6, 133.1, 133.0 (t, *J* = 4.2 Hz), 132.4 (t, *J* = 1.3 Hz), 129.1, 127.5, 125.8, 123.4 (t, *J* = 243.6 Hz), 80.7, 42.4 (t, *J* = 26.2 Hz), 31.5 (t, *J* = 24.9 Hz), 28.3 (t, *J* = 4.5 Hz), 28.1.

^{19}F NMR (282 MHz, $CDCl_3$) δ: –98.7 (quint, *J* = 16.0 Hz).

HRMS (ESI): calcd for $C_{15}H_{19}BrF_2O_2Na$ (M+Na) 371.0429, 373.0409; found 371.0418, 373.0400.

tert-Butyl 4,4-difluoro-5-[4-(4,4,5,5-tetramethyl-1,3,2-dioxaborolan-2-yl)phenyl]pentanoate (3d).

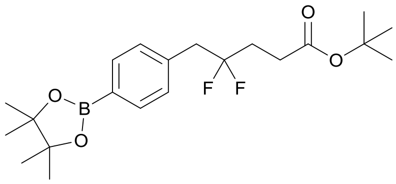

Yield 141 mg (71%). Colorless crystals. Mp 51–53 °C. Chromatography: hexanes/EtOAc, from 8/1 to 5/1. R_f 0.44 (hexanes/EtOAc, 5/1).

^{1}H NMR (300 MHz, $CDCl_3$) δ: 7.79 (d, *J* = 7.6 Hz, 2H), 7.28 (d, *J* = 7.6 Hz, 2H), 3.22 (t, *J* = 15.9 Hz, 2H), 2.43 (t, *J* = 7.8 Hz, 2H), 2.16–1.99 (m, 2H), 1.43 (s, 9H), 1.35 (s, 12H).

^{13}C{^{1}H} NMR (75.5 MHz, $CDCl_3$) δ: 171.7, 136.3 (t, *J* = 4.7 Hz), 135.1, 129.8, 123.5 (t, *J* = 243.2 Hz), 83.9, 80.8, 43.7 (t, *J* = 25.9 Hz), 31.3 (t, *J* = 25.1 Hz), 28.3 (t, *J* = 4.5 Hz), 28.1, 25.0.

^{19}F NMR (282 MHz, $CDCl_3$) δ: –98.7 (quint, *J* = 16.5 Hz).

HRMS (ESI): calcd for $C_{21}H_{31}BF_2O_4Na$ (M+Na) 419.2179; found 419.2171.

***tert*-Butyl 4,4-difluoro-5-phenylhexanoate (3e).**

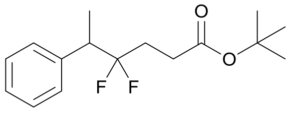

Yield 107 mg (75%). Colorless oil. Chromatography: hexanes/EtOAc, 10/1. R_f 0.42 (hexanes/EtOAc, 10/1).

^{1}H NMR (300 MHz, $CDCl_3$) δ: 7.38–7.29 (m, 5H), 3.24–3.12 (m, 1H), 2.46–2.40 (m, 2H), 2.11-1.94 (m, 2H), 1.47–1.43 (m, 12H).

^{13}C{^{1}H} NMR (75.5 MHz, $CDCl_3$) δ: 171.9, 139.6 (dd, *J* = 6.1, 1.3 Hz), 128.7 (t, *J* = 1.2 Hz), 128.6, 127.5, 124.7 (dd, *J* = 246.7, 245.1 Hz), 80.6, 46.8 (dd, *J* = 24.7, 23.8 Hz), 30.4 (t, *J* = 25.2 Hz), 28.1, 28.1 (t, *J* = 4.7 Hz), 14.8 (t, *J* = 5.0 Hz).

^{19}F NMR (282 MHz, $CDCl_3$) δ: –104.2 (dm, *J* = 241.3 Hz, 1F), –109.3 (dm, *J* = 241.3 Hz, 1F).

HRMS (ESI): calcd for $C_{16}H_{23}F_2O_2$ (M+H) 285.1661; found 285.1653.

7-(*tert*-Butoxy)-4,4-difluoro-7-oxoheptyl benzoate (3f).

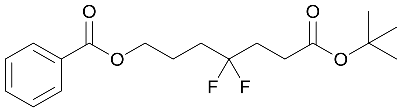

Yield 128 mg (75%). Colorless oil. Chromatography: hexanes/EtOAc, 8/1. R_f 0.25 (hexanes/EtOAc, 8/1).

^{1}H NMR (300 MHz, $CDCl_3$) δ: 8.02 (d, *J* = 7.1, Hz, 2H), 7.54 (t, *J* = 7.4, Hz, 1H), 7.42 (dd, *J* = 7.4, 7.1 Hz, 2H), 4.34 (t, *J* = 6.0 Hz, 2H), 2.43 (t, *J* = 7.8 Hz, 2H), 2.24–1.94 (m, 6H), 1.43 (s, 9H).

^{13}C{^{1}H} NMR (75.5 MHz, $CDCl_3$) δ: 171.6, 166.5, 133.0, 130.2, 129.6, 128.4, 124.0 (t, *J* = 241.0 Hz), 80.8, 64.1, 33.5 (t, *J* = 25.7 Hz), 31.9 (t, *J* = 25.7 Hz), 28.4 (t, *J* = 4.6 Hz), 28.1, 21.9 (t, *J* = 4.6 Hz).

^{19}F NMR (282 MHz, $CDCl_3$) δ: –101.1 (quint, *J* = 16.5 Hz).

HRMS (ESI): calcd for $C_{18}H_{24}F_2O_4Na$ (M+Na) 365.1535; found 365.1536.

4,4-Difluoro-*N,N*-diisopropyl-5-phenylpentanamide (3g).

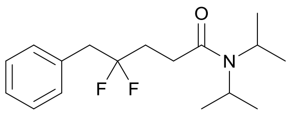

Yield 105 mg (71%). Colorless oil. Chromatography: hexanes/EtOAc, 5/1. R_f 0.26 (hexanes/EtOAc, 5/1).

^{1}H NMR (300 MHz, $CDCl_3$) δ: 7.35–7.24 (m, 5H), 3.94 (sept, *J* = 6.7 Hz, 1H), 3.47 (br. s, 1H), 3.17 (t, *J* = 15.9 Hz, 2H), 2.51–2.46 (m, 2H), 2.25–2.07 (m, 2H), 1.35 (d, *J* = 6.8 Hz, 6H), 1.17 (d, *J* = 6.7 Hz, 6H).

^{13}C{^{1}H} NMR (75.5 MHz, $CDCl_3$) δ: 169.9, 133.3 (t, *J* = 5.0 Hz), 130.4, 128.5, 127.3, 124.1 (t, *J* = 242.6 Hz), 48.3, 45.7, 43.8 (t, *J* = 26.1 Hz), 31.5 (t, *J* = 24.1 Hz), 27.2 (t, *J* = 3.6 Hz), 20.8, 20.7.

^{19}F NMR (282 MHz, $CDCl_3$) δ: –99.0 (quint, *J* = 15.9 Hz).

HRMS (ESI): calcd for $C_{17}H_{26}F_2NO$ (M+H) 298.1977; found 298.1979.

4,4-Difluoro-*N,N*-diisopropyl-5-phenylhexanamide (3h).

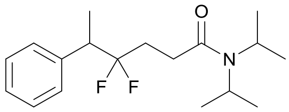

Yield 109 mg (70%). Colorless oil. Chromatography: hexanes/EtOAc, 5/1. R_f 0.27 (hexanes/EtOAc, 5/1).

^{1}H NMR (300 MHz, $CDCl_3$) δ: 7.36–7.22 (m, 5H), 3.92 (sept, *J* = 6.7 Hz, 1H), 3.45 (br. s, 1H), 3.27–3.10 (m, 1H), 2.19–1.95 (m, 2H), 1.44 (d, *J* = 7.2 Hz, 3H), 1.34 (d, *J* = 6.7 Hz, 6H), 1.15 (d, *J* = 6.7 Hz, 6H).

^{13}C{^{1}H} NMR (75.5 MHz, $CDCl_3$) δ: 170.1, 139.6 (dd, *J* = 5.7, 1.7 Hz), 128.8 (t, *J* = 1.2 Hz), 128.5, 127.4, 125.3 (dd, *J* = 246.6, 245.0 Hz), 48.1, 47.0 (t, *J* = 24.5 Hz), 45.7, 30.5 (t, *J* = 24.2 Hz), 27.2 (t, *J* = 3.6 Hz), 20.8, 20.7, 14.8 (t, *J* = 5.0 Hz).

^{19}F NMR (282 MHz, $CDCl_3$) δ: –103.6 (ddt, *J* = 239.4, 28.2, 9.5 Hz), –108.9 (dddd, *J* = 239.4, 27.8, 18.9, 8.4 Hz).

HRMS (ESI): calcd for $C_{18}H_{28}F_2NO$(M+H) 312.2133; found 312.2140.

4,4-Difluoro-5-hydroxy-*N,N*-diisopropyl-5-phenylpentanamide (3i).

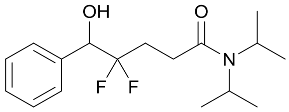

Yield 114 mg (73%). Colorless crystals. Mp 80-82°C (hexanes). Chromatography: hexanes/EtOAc, from 3/1 to 1/1. R_f 0.26 (hexanes/EtOAc, 3/1).

^{1}H NMR (300 MHz, $CDCl_3$) δ: 7.49–7.30 (m, 5H), 4.73 (ddd, *J* = 13.1, 8.5, 4.2 Hz, 1H), 4.47 (d, *J* = 4.3 Hz, 1H), 3.95 (sept, *J* = 6.8 Hz, 1H), 3.49 (br. s, 1H), 2.60–2.39 (m, 3H), 2.19–2.00 (m, 1H), 1.34 (d, *J* = 6.8 Hz, 6H), 1.20 (d, *J* = 6.8 Hz, 3H), 1.19 (d, *J* = 6.8 Hz, 3H).

^{13}C{^{1}H} NMR (75.5 MHz, $CDCl_3$) δ: 170.7, 136.8 (t, *J* = 1.8 Hz), 128.4, 128.2, 127.8 (t, *J* = 1.3 Hz), 123.6 (dd, *J* = 248.2, 246.1 Hz), 74.5 (dd, *J* = 29.2, 28.0 Hz), 48.4, 46.0, 28.0 (t, *J* = 24.2 Hz), 27.2 (dd, *J* = 5.5, 3.4 Hz), 20.9, 20.8, 20.7, 20.6.

^{19}F NMR (282 MHz, $CDCl_3$) δ: –109.0 (dm, *J* = 248.7 Hz), –110.4 (dm, *J* = 248.7 Hz).

HRMS (ESI): calcd for $C_{17}H_{26}F_2NO_2$ (M+H) 314.1926; found 314.1938.

4,4-Difluoro-5-hydroxy-*N,N*-diisopropyl-5-(4-methoxyphenyl)pentanamide (3j).

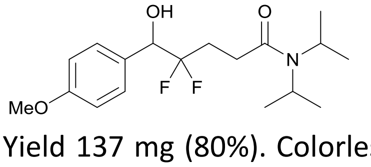

Yield 137 mg (80%). Colorless oil. Chromatography: hexanes/EtOAc, 2/1. R_f 0.26 (hexanes/EtOAc, 2/1).

^{1}H NMR (300 MHz, $CDCl_3$) δ: 7.36 (d, *J* = 8.5 Hz, 2H), 6.86 (d, *J* = 8.5 Hz, 2H), 4.67 (ddd, *J* = 13.9, 9.0, 3.9 Hz, 1H), 4.36 (d, *J* = 4.1 Hz, 1H), 3.94 (sept, *J* = 6.6 Hz, 1H), 3.77 (s, 3H), 3.47 (br. s, *J* = 3.47 Hz, 1H). 2.56–2.02 (m, 4H), 1.32 (d, *J* = 6.7 Hz, 6H), 1.17 (d, *J* = 6.5 Hz, 3H), 1.16 (d, *J* = 6.5 Hz, 3H).

^{13}C{^{1}H} NMR (75.5 MHz, $CDCl_3$) δ: 170.6, 159.7, 129.0, 123.7 (t, *J* = 246.8 Hz), 113.6, 74.2 (t, *J* = 28.5 Hz), 55.3, 48.4, 46.0, 28.0 (t, *J* = 24.2 Hz), 27.2 (t, *J* = 3.8 Hz), 20.84, 20.82, 20.63, 20.60.

^{19}F NMR (282 MHz, $CDCl_3$) δ: –109.5 (dm, *J* = 246.1 Hz, 1F), –110.6 (dm, *J* = 246.1 Hz, 1F).
HRMS (ESI): calcd for $C_{18}H_{28}F_2NO_3$ (M+H) 344.2032; found 344.2035.

4,4-Difluoro-5-phenylpentanenitrile (3k).

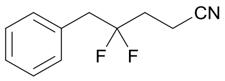

Yield 83 mg (85%). Colorless oil. Chromatography: hexanes/EtOAc, from 5/1 to 3/1. R_f 0.24 (hexanes/EtOAc, 5/1).
^{1}H NMR (300 MHz, $CDCl_3$) δ: 7.41–7.25 (m, 5H), 3.21 (t, *J* = 16.0 Hz, 2H), 2.53 (t, *J* = 7.7 Hz, 2H), 2.15 (tt, *J* = 16.0, 7.7 Hz, 2H).
$^{13}C\{^1H\}$ NMR (75.5 MHz, $CDCl_3$) δ: 132.3 (t, *J* = 4.9 Hz), 130.2, 128.7, 127.7, 122.3 (t, *J* = 243.9 Hz), 118.5, 43.1 (t, *J* = 25.4 Hz), 31.7 (t, *J* = 25.5 Hz), 10.3 (t, *J* = 5.9 Hz).
^{19}F NMR (282 MHz, $CDCl_3$) δ: –100.0 (quint, *J* = 16.0 Hz).
HRMS (ESI): calcd for $C_{11}H_{11}F_2NNa$ (M+Na) 218.0752; found 218.0760.

4,4-Difluoro-5-phenylhexanenitrile (3l).

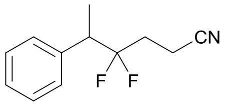

Yield 90 mg (86%). Colorless oil. Chromatography: hexanes/EtOAc, 3/1. R_f 0.43 (hexanes/EtOAc, 3/1).
^{1}H NMR (300 MHz, $CDCl_3$) δ: 7.41–7.26 (m, 5H), 3.28–3.10 (m, 1H), 2.50 (ddd, *J* = 10.5, 5.3, 2.5 Hz, 2H), 2.17–1.94 (m, 2H), 1.48 (d, *J* = 7.2 Hz, 3H).
$^{13}C\{^1H\}$ NMR (75.5 MHz, $CDCl_3$) δ: 138.7 (dd, *J* = 6.5, 1.1 Hz), 128.8, 128.5, 127.9, 123.5 (dd, *J* = 247.7, 246.2 Hz), 118.7, 46.6 (dd, *J* = 24.2, 23.2 Hz), 31.2 (t, *J* = 25.7 Hz), 14.4 (t, *J* = 4.8 Hz), 10.3 (t, *J* = 6.2 Hz).
^{19}F NMR (282 MHz, $CDCl_3$) δ: –104.5 (ddt, *J* = 242.8, 25.9, 8.7 Hz, 1F), –111.2 (dddd, *J* = 242.8, 27.0, 20.8, 7.2 Hz, 1F).
HRMS (ESI): calcd for $C_{12}H_{13}F_2NNa$ (M+Na) 232.0908; found 232.0909.

4,4-Difluoro-5-hydroxy-5-phenylpentanenitrile (3m).[3]

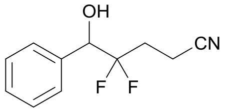

Yield 82 mg (78%). Colorless oil. Chromatography: hexanes/EtOAc, 1/1. R_f 0.36 (hexanes/EtOAc, 1/1).
^{1}H NMR (300 MHz, $CDCl_3$) δ: 7.44–7.35 (m, 5H), 4.86 (td, *J* = 9.9, 3.8 Hz, 1H), 3.12 (d, *J* = 4.1 Hz, 1H), 2.50 (dd, *J* = 7.2, 3.3 Hz, 1H), 2.47 (dd, *J* = 6.7, 2.2 Hz, 1H), 2.42–1.95 (m, 2H).
$^{13}C\{^1H\}$ NMR (75.5 MHz, $CDCl_3$) δ: 136.0 (dd, *J* = 4.0, 1.0 Hz), 129.0, 128.5, 127.3, 121.9 (d, *J* = 248.3 Hz), 118.8, 74.8 (t, *J* = 28.4 Hz), 28.1 (t, *J* = 24.4 Hz), 10.1 (t, *J* = 6.0 Hz).

[3] Panferova, L. I.; Tsymbal, A. V.; Levin, V. V.; Struchkova, M. I.; Dilman, A. D. *Org. Lett.* **2016**, *18*, 996–999.

^{19}F NMR (282 MHz, $CDCl_3$) δ: –110.2 (ddt, *J* = 249.3, 25.7, 9.0 Hz, 1F), –111.7 (ddt, *J* = 249.7, 25.9, 9.1 Hz, 1F).

HRMS (ESI): calcd for $C_{11}H_{11}F_2NONa$ (M+Na) 234.0701; found 234.0707.

5-(4-Bromophenyl)-4,4-difluoropentanenitrile (3n).

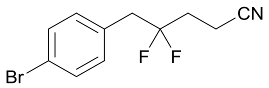

Yield 106 mg (77%). Colorless crystals. Mp 79-80°C. Chromatography: hexanes/EtOAc, 3/1. R_f 0.36 (hexanes/EtOAc, 3/1).

^1H NMR (300 MHz, $CDCl_3$) δ: 7.47 (d, *J* = 8.3 Hz, 2H), 7.13 (d, *J* = 8.3 Hz, 2H), 3.14 (t, *J* = 16.0 Hz, 2H), 2.53 (t, *J* = 7.6 Hz, 2H), 2.21–2.05 (m, 2H).

$^{13}C\{^1H\}$ NMR (75.5 MHz, $CDCl_3$) δ: 132.0, 131.9, 131.2 (t, *J* = 4.8 Hz), 122.0, 121.9 (t, *J* = 244.4 Hz), 118.4, 42.6 (t, *J* = 25.6 Hz), 31.9 (t, *J* = 25.4 Hz), 10.4 (t, *J* = 5.9 Hz).

^{19}F NMR (282 MHz, $CDCl_3$) δ: –100.4 (quint, *J* = 16.0 Hz).

HRMS (ESI): calcd for $C_{11}H_{10}BrF_2NNa$ (M+Na) 295.9857, 297.9837; found 295.9853, 297.9835.

5-(2-Bromophenyl)-4,4-difluoropentanenitrile (3o).

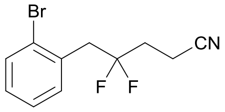

Yield 112 mg (82%). Colorless oil. Chromatography: hexanes/EtOAc, 3/1. R_f 0.37 (hexanes/EtOAc, 3/1).

^1H NMR (300 MHz, $CDCl_3$) δ: 7.60 (dd, *J* = 7.8, 1.5 Hz, 1H), 7.36 (d, *J* = 7.8 Hz, 1H), 7.30 (td, *J* = 7.8 1.5 Hz, 1H), 7.18 (td, *J* = 7.8 1.5 Hz, 1H), 3.45 (t, *J* = 15.7 Hz, 2H), 2.55 (t, *J* = 7.7 Hz, 2H), 2.30–2.15 (m, 2H).

$^{13}C\{^1H\}$ NMR (75.5 MHz, $CDCl_3$) δ: 133.3, 132.4, 132.2 (t, *J* = 4.8 Hz), 129.5, 127.8, 125.5, 122.2 (t, *J* = 244.9 Hz), 118.5, 42.2 (t, *J* = 25.9 Hz), 31.9 (t, *J* = 25.1 Hz), 10.4 (t, *J* = 6.0 Hz).

^{19}F NMR (282 MHz, $CDCl_3$) δ: –99.4 (quint, *J* = 15.7 Hz).

HRMS (ESI): calcd for $C_{11}H_{10}BrF_2NNa$ (M+Na) 295.9857, 297.9837; found 295.9849, 297.9831.

Methyl 4-(4-cyano-2,2-difluorobutyl)benzoate (3p).

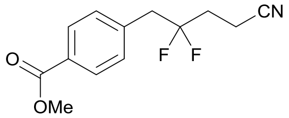

Yield 97 mg (77%). Colorless crystals. Mp 51–53°C. Chromatography: hexanes/EtOAc, from 3/1 to 2/1. R_f 0.18 (hexanes/EtOAc, 3/1).

^1H NMR (300 MHz, $CDCl_3$) δ: 7.99 (d, *J* = 8.2 Hz, 2H), 7.31 (d, *J* = 8.2 Hz, 2H), 3.89 (s, 3H), 3.22 (t, *J* = 16.0 Hz, 2H), 2.52 (t, *J* = 7.6 Hz, 2H), 2.21–2.05 (m, 2H).

$^{13}C\{^1H\}$ NMR (75.5 MHz, $CDCl_3$) δ: 166.6, 137.3 (t, *J* = 4.6 Hz), 130.3, 129.9, 129.7, 121.9 (t, *J* = 244.3 Hz), 118.4, 52.1, 43.0 (t, *J* = 25.5 Hz), 32.0 (t, *J* = 25.5 Hz), 10.3 (t, *J* = 5.9 Hz).

^{19}F NMR (282 MHz, $CDCl_3$) δ: –100.1 (quint, *J* = 16.0 Hz).

HRMS (ESI): calcd for $C_{13}H_{14}F_2NO_2$ (M+H) 254.0987; found 254.0988.

6-Cyano-4,4-difluorohexyl benzoate (3q).

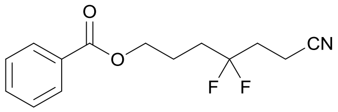

Yield 110 mg (82%). Colorless oil. Chromatography: hexanes/EtOAc, 2/1. R_f 0.32 (hexanes/EtOAc, 2/1).

^{1}H NMR (300 MHz, $CDCl_3$) δ: 8.06–8.00 (m, 2H), 7.60–7.52 (m, 1H), 7.44 (t, *J* = 7.7 Hz, 2H), 4.35 (t, *J* = 6.0 Hz, 2H), 2.56 (t, *J* = 7.7 Hz, 2H), 2.30–2.15 (m, 2H), 2.12–1.93 (m, 4H).

^{13}C{^{1}H} NMR (75.5 MHz, $CDCl_3$) δ: 166.4, 133.1, 130.0, 129.5, 128.4, 122.8 (t, *J* = 242.9 Hz), 118.5, 63.8, 33.3 (t, *J* = 25.1 Hz), 32.6 (t, *J* = 26.1 Hz), 21.7 (t, *J* = 4.9, Hz) , 10.5 (t, *J* =5.8 Hz).

^{19}F NMR (282 MHz, $CDCl_3$) δ: –102.4 (quint, *J* = 16.2 Hz).

HRMS (ESI): calcd for $C_{14}H_{16}F_2NO_2$ (M+H) 268.1144; found 268.1155.

Diethyl (3,3-difluoro-4-phenylbutyl)phosphonate (3r).

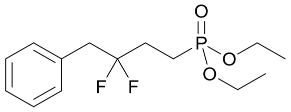

Yield 124 mg (81%). Colorless oil. Chromatography: EtOAc, R_f 0.22 (EtOAc).

^{1}H NMR (300 MHz, $CDCl_3$) δ: 7.32–7.22 (m, 5H), 4.10–3.99 (m, 4H), 3.13 (t, *J* = 15.9 Hz, 2H), 2.12–1.83 (m, 4H), 1.26 (t, *J* = 7.1 Hz, 6H).

^{13}C{^{1}H} NMR (75.5 MHz, $CDCl_3$) δ: 132.8 (t, *J* = 4.8 Hz), 130.2, 128.5, 127.5, 123.0 (td, *J* = 243.4, 19.0 Hz), 61.7 (d, *J* = 6.5 Hz), 43.1 (t, *J* = 29.5 Hz), 29.3 (td, *J* = 26.1, 3.7 Hz), 18.5 (dt, *J* = 145.7, 4.6 Hz), 16.3 (d, *J* = 6.0 Hz).

^{19}F NMR (282 MHz, $CDCl_3$) δ: –99.7 (quint, *J* = 15.9 Hz).

^{31}P NMR (121 MHz, $CDCl_3$) δ: 30.3.

HRMS (ESI): calcd for $C_{14}H_{22}F_2O_3P$ (M+H) 307.1269; found 307.1272.

6-(Diethoxyphosphoryl)-4,4-difluorohexyl benzoate (3s).

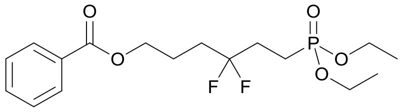

Yield 132 mg (70%). Colorless oil. Chromatography: EtOAc. R_f 0.36 (EtOAc).

^{1}H NMR (300 MHz, $CDCl_3$) δ: 8.00 (d, *J* = 7.1 Hz, 2H), 7.52 (t, *J* = 7.4 Hz, 1H), 7.40 (t, *J* = 7.5 Hz, 2H), 4.32 (t, *J* = 5.5 Hz, 2H), 4.06 (td, *J* = 7.1, 2.9 Hz, 4H), 2.22–1.85 (m, 8H), 1.29 (t, *J* = 7.1 Hz, 6H).

^{13}C{^{1}H} NMR (75.5 MHz, $CDCl_3$) δ: 166.4, 133.0, 130.1, 129.6, 128.4, 123.6 (td, *J* = 242.0, 18.8 Hz), 64.0, 61.9 (d, *J* = 6.5 Hz), 33.3 (t, *J* = 25.7 Hz), 30.4 (td, *J* = 26.6, 4.1 Hz), 21.9 (t, *J* = 4.7 Hz), 18.6 (dt, *J* = 145.6, 4.6 Hz), 16.4 (d, *J* = 5.8 Hz).

^{19}F NMR (282 MHz, $CDCl_3$) δ: –102.2 (quint, *J* = 16.0 Hz).

^{31}P{^{1}H} NMR (121 MHz, $CDCl_3$) δ: 30.3.

HRMS (ESI): calcd for $C_{17}H_{26}F_2O_5P$ (M+H) 379.1480; found 379.1491.

[2,2-Difluoro-4-(phenylsulfonyl)butyl]benzene (3t).

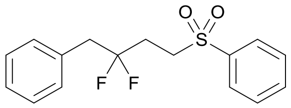

Yield 133 mg (86%). Colorless crystals. Mp 63–64°C (hexanes). Chromatography: hexanes/EtOAc, from 3/1 to 2/1. R_f 0.33 (hexanes/EtOAc, 3/1).

^{1}H NMR (300 MHz, $CDCl_3$) δ: 7.87 (dd, *J* = 7.2, 1.4 Hz, 2H), 7.67 (tt, *J* = 7.4, 1.3 Hz, 1H), 7.56 (tt, *J* = 7.5, 1.2 Hz, 2H), 7.33–7.28 (m, 3H), 7.22–7.17 (m, 2H), 3.28–3.22 (m, 2H), 3.15 (t, *J* = 15.9 Hz, 2H), 2.33–2.16 (m, 2H).

^{13}C{^{1}H} NMR (75.5 MHz, $CDCl_3$) δ: 138.9, 134.1, 132.3 (t, *J* = 4.9 Hz), 130.3, 129.6, 128.8, 128.1, 127.9, 122.5 (t, *J* = 244.1 Hz), 49.7 (t, *J* = 3.8 Hz), 43.6 (t, *J* = 25.5 Hz), 29.3 (t, *J* = 25.7 Hz).

^{19}F NMR (282 MHz, $CDCl_3$) δ: -98.3 (quint, *J* = 15.9 Hz).

HRMS (ESI): calcd for $C_{16}H_{16}F_2O_2SNa$ (M+Na) 333.0731; found 333.0738.

[(3,3-Difluoro-4-phenylpentyl)sulfonyl]benzene (3u).

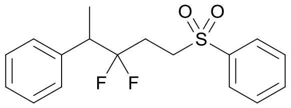

Yield 113 mg (70%). Colorless crystals. Mp 96–98°C (hexanes/EtOAc, 1/1). Chromatography: hexanes/EtOAc, 3/1. R_f 0.33 (hexanes/EtOAc, 3/1).

^{1}H NMR (300 MHz, $CDCl_3$) δ: 7.84 (dd, *J* = 7.2, 1.4 Hz, 2H), 7.65 (tt, *J* = 7.4, 1.3 Hz, 1H), 7.53 (t, *J* = 7.5, 1.4 Hz, 2H), 7.34–7.25 (m, 3H), 7.25–7.20 (m, 2H), 3.31–3.07 (m, 3H), 2.28–2.08 (m, 2H), 1.42 (d, *J* = 7.2 Hz, 3H).

^{13}C{^{1}H} NMR (75.5 MHz, $CDCl_3$) δ: 138.7, 138.6 (dd, *J* = 6.2, 1.2 Hz), 134.0, 129.4, 128.7, 128.5 (t, *J* = 1.5 Hz), 127.9, 127.8, 123.7 (t, *J* = 248.1, 246.3 Hz), 49.6 (t, *J* = 4.3 Hz), 46.7 (dd, *J* = 24.2, 23.3 Hz), 28.5 (t, *J* = 26.0 Hz), 14.5 (t, *J* = 4.8 Hz).

^{19}F NMR (282 MHz, $CDCl_3$) δ: –102.6 (dm, *J* = 240.4 Hz, 1F), –108.6 (dm, *J* = 240.4 Hz, 1F).

HRMS (ESI): calcd for $C_{17}H_{18}F_2O_2SNa$ (M+Na) 347.0888; found 347.0881.

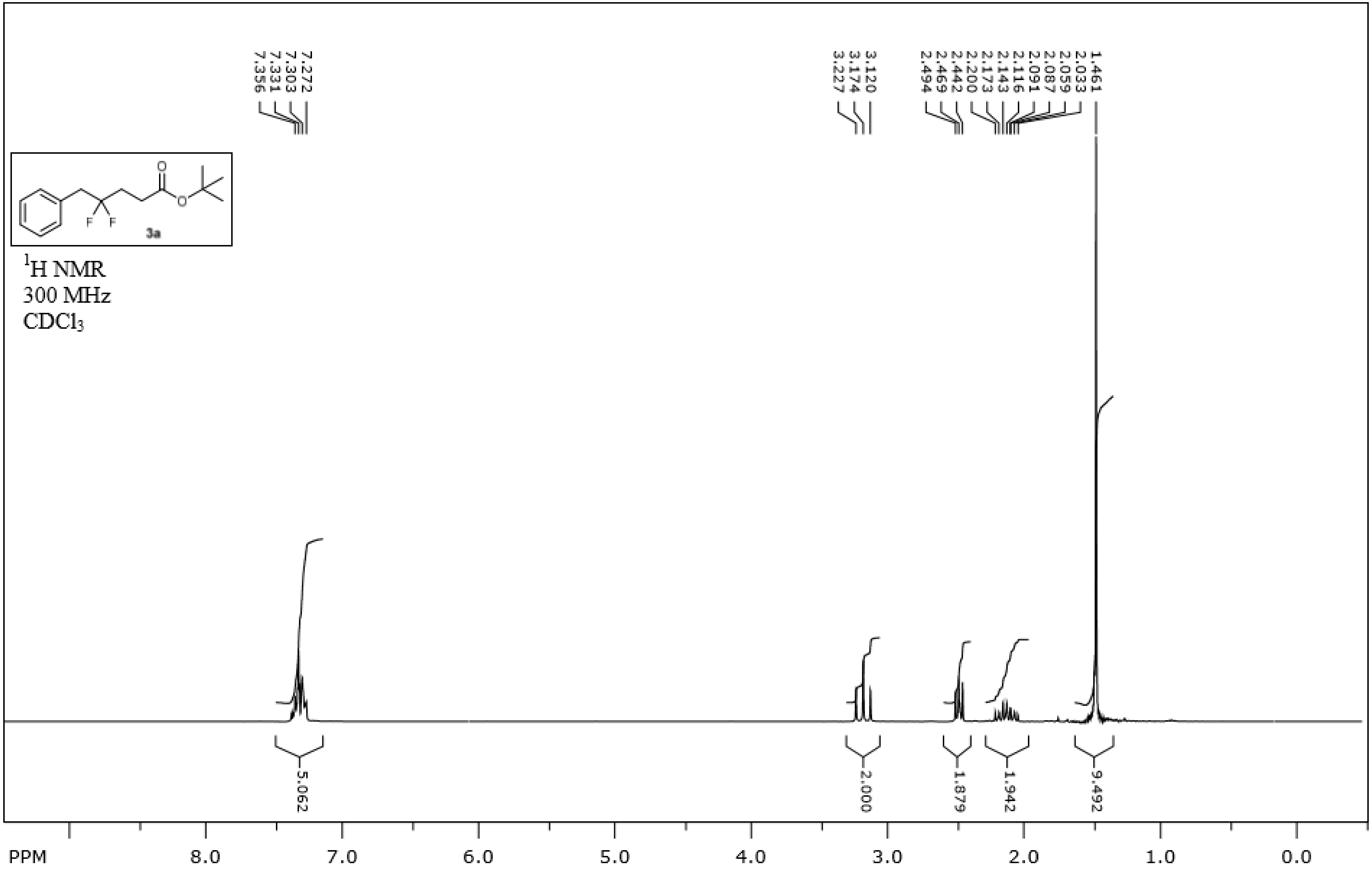

3a
¹H NMR
300 MHz
CDCl3
7.356
7.331
7.303
7.272
3.227
3.174
3.120
2.494
2.469
2.442
2.200
2.173
2.143
2.116
2.091
2.087
2.059
2.033
1.461
5.062
2.000
1.879
1.942
9.492
PPM
8.0
7.0
6.0
5.0
4.0
3.0
2.0
1.0
0.0

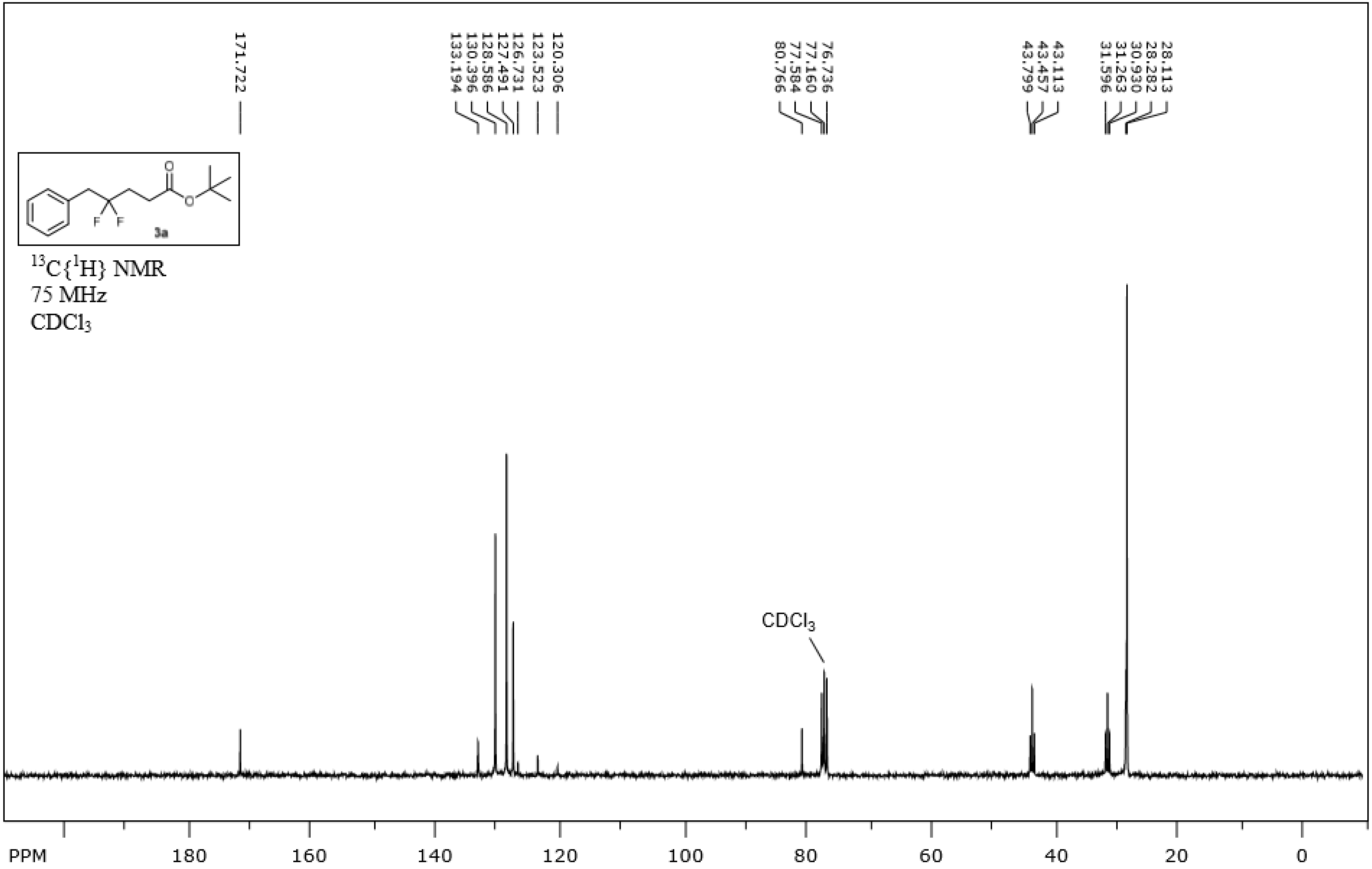

$^{13}C\{^1H\}$ NMR
75 MHz
$CDCl_3$

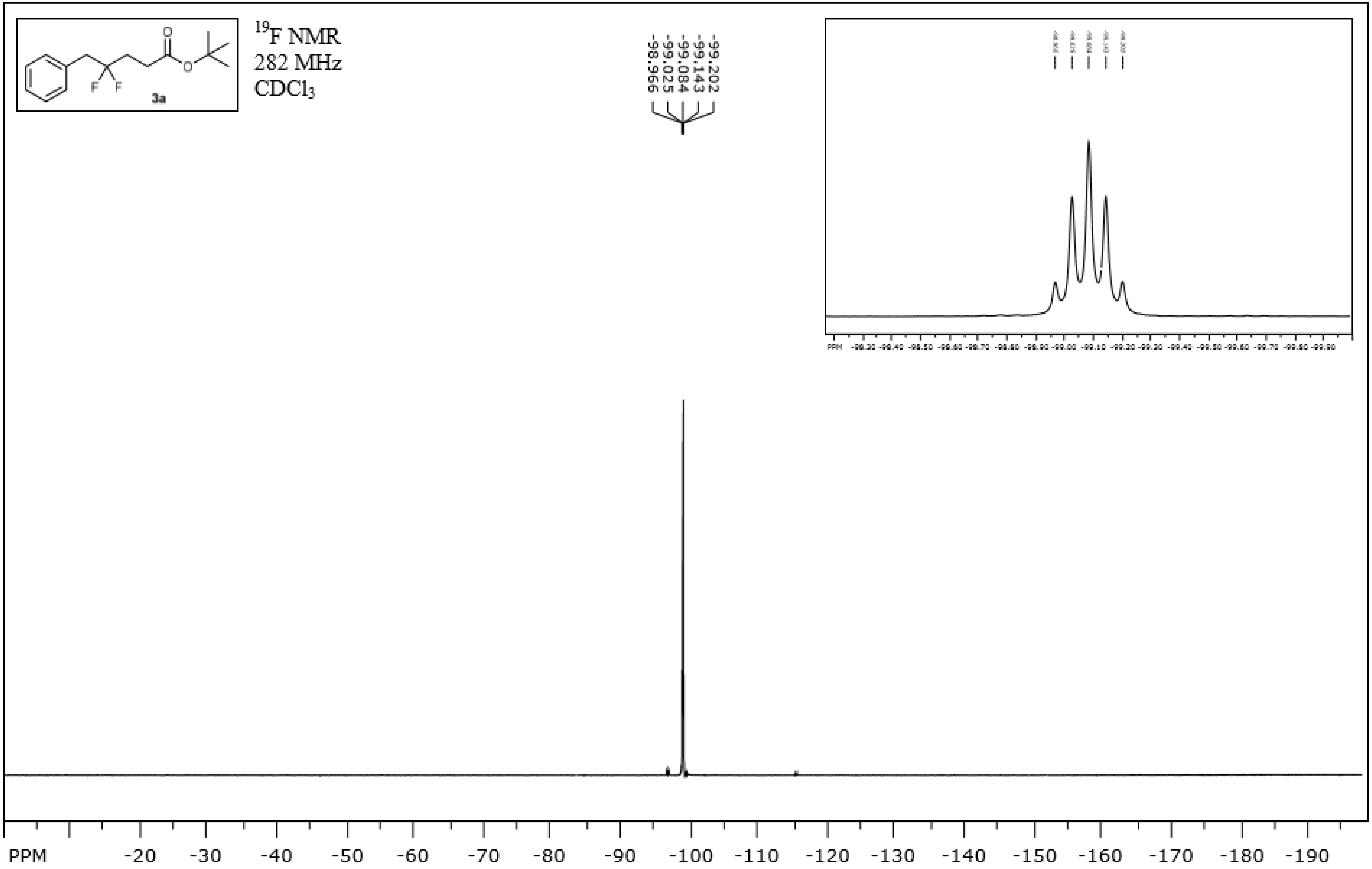

3a
19F NMR
282 MHz
CDCl3
-98.966
-99.025
-99.084
-99.143
-99.202
PPM
-20
-30
-40
-50
-60
-70
-80
-90
-100
-110
-120
-130
-140
-150
-160
-170
-180
-190

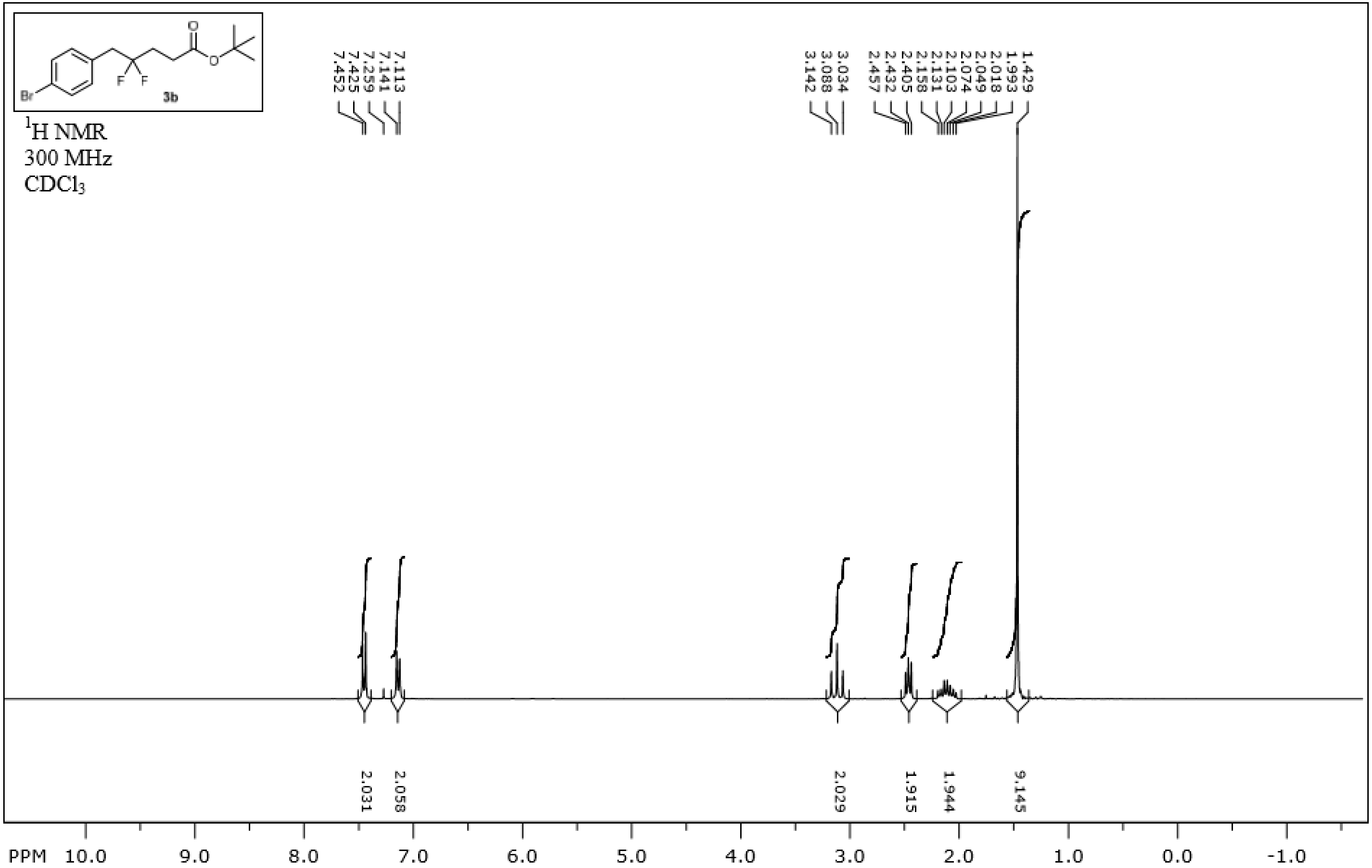

Br
F F
3b
1H NMR
300 MHz
CDCl3
7.452
7.425
7.259
7.141
7.113
3.142
3.088
3.034
2.457
2.432
2.405
2.158
2.131
2.103
2.074
2.049
2.018
1.993
1.429
2.031
2.058
2.029
1.915
1.944
9.145
PPM 10.0 9.0 8.0 7.0 6.0 5.0 4.0 3.0 2.0 1.0 0.0 -1.0

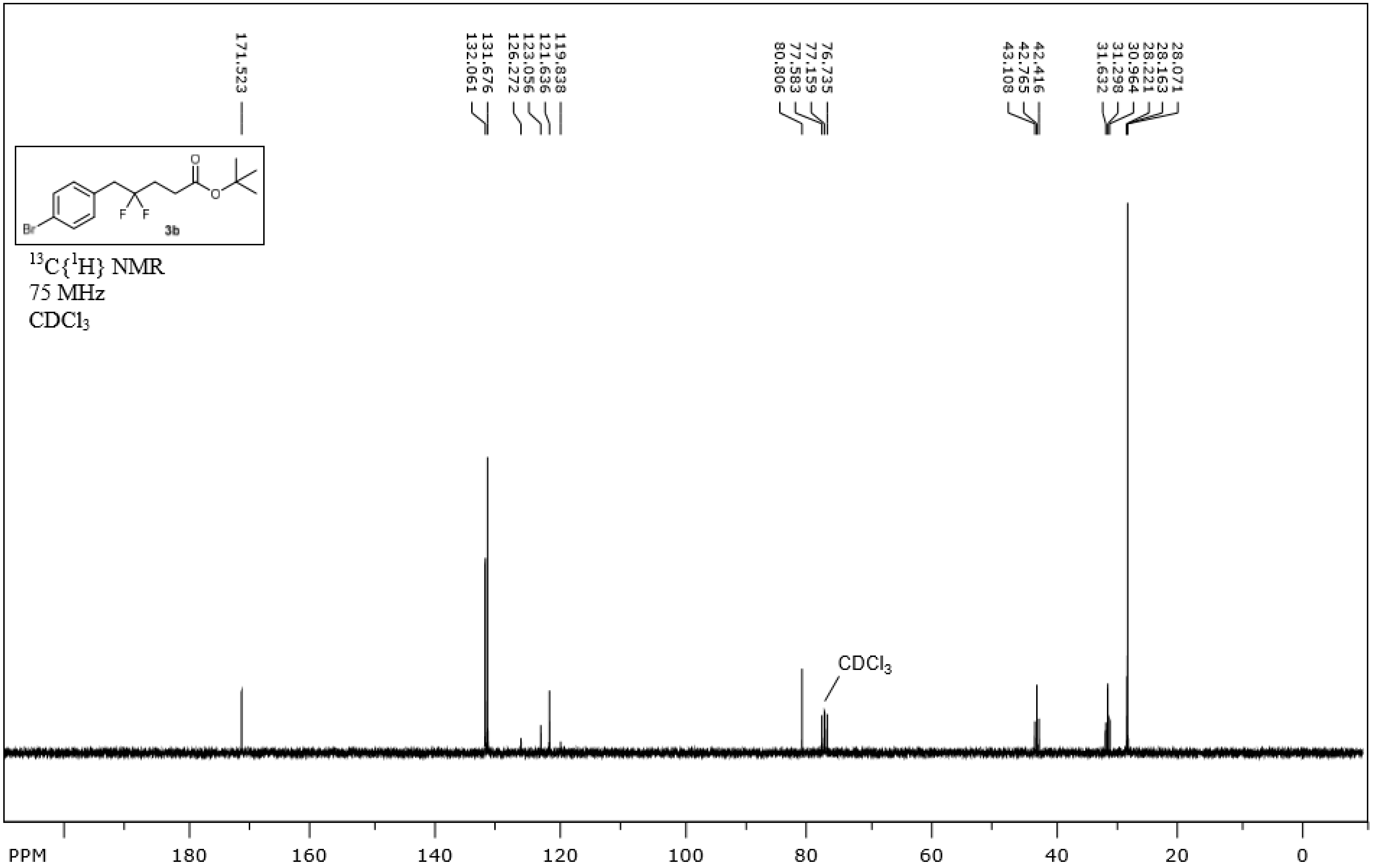
171.523
132.061
131.676
126.272
123.056
121.636
119.838
80.806
77.583
77.159
76.735
43.108
42.765
42.416
31.632
31.298
30.964
28.221
28.163
28.071
Br
F
F
O
O
3b
$^{13}C\{^{1}H\}$ NMR
75 MHz
$CDCl_3$
$CDCl_3$
PPM
180
160
140
120
100
80
60
40
20
0

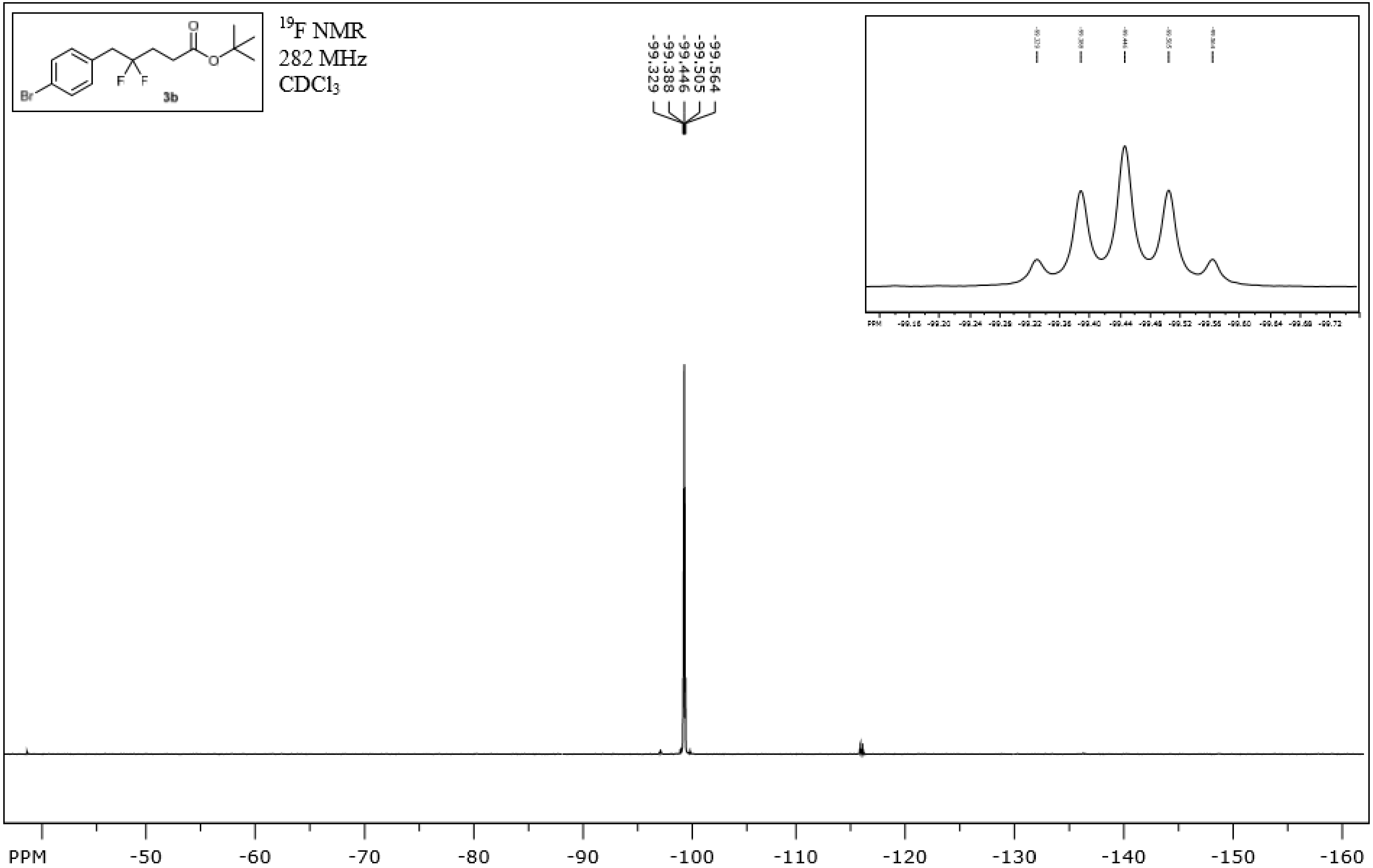

3b
^{19}F NMR
282 MHz
$CDCl_3$
-99.329
-99.388
-99.446
-99.505
-99.564
PPM
-50
-60
-70
-80
-90
-100
-110
-120
-130
-140
-150
-160

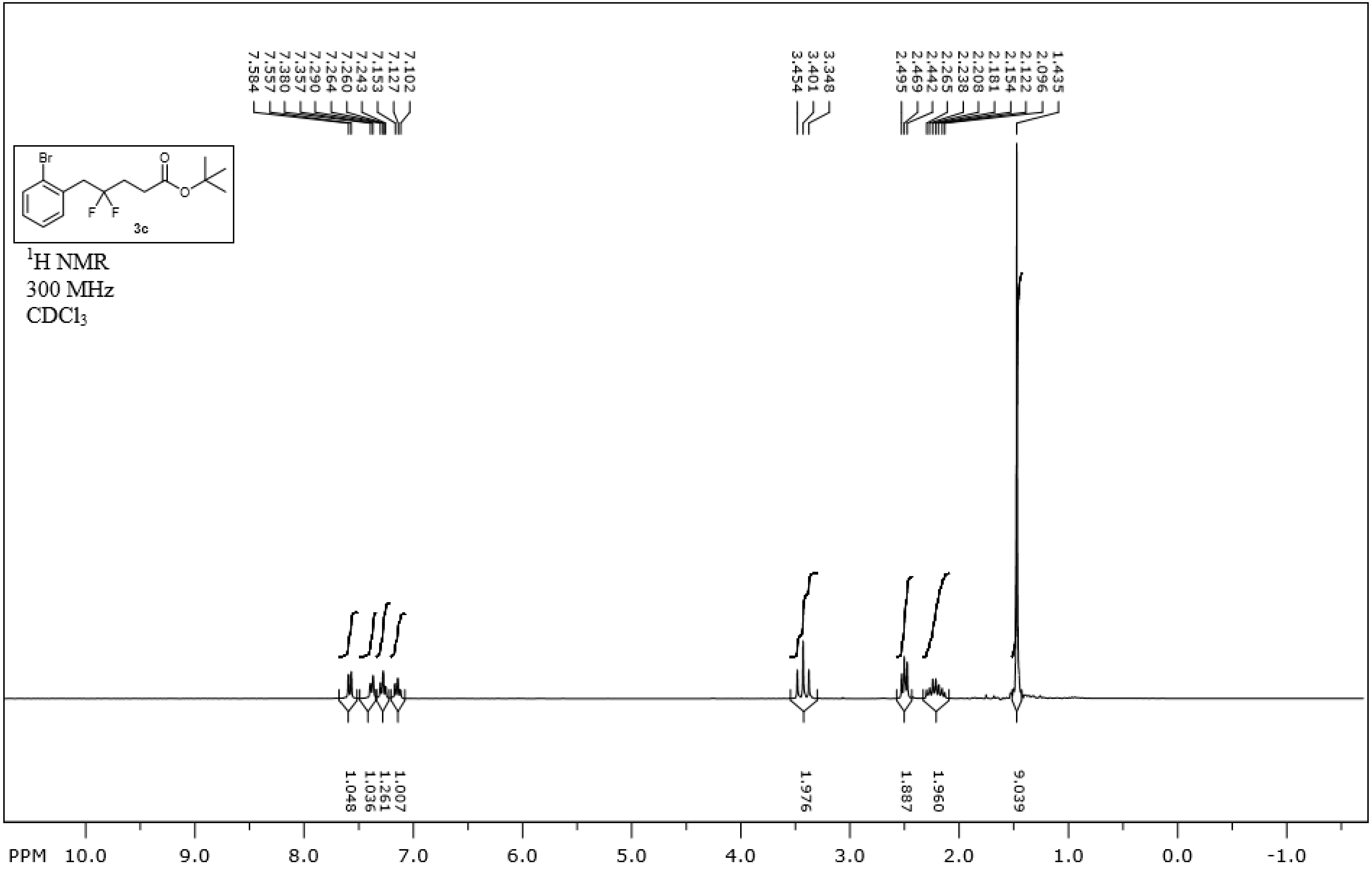
Br
O
O
F F
3c
^{1}H NMR
300 MHz
$CDCl_3$
7.584 7.557 7.380 7.357 7.290 7.264 7.260 7.243 7.153 7.127 7.102
3.454 3.401 3.348
2.495 2.469 2.442 2.265 2.238 2.208 2.181 2.154 2.122 2.096 1.435
1.048 1.036 1.261 1.007
1.976
1.887
1.960
9.039
PPM 10.0 9.0 8.0 7.0 6.0 5.0 4.0 3.0 2.0 1.0 0.0 -1.0

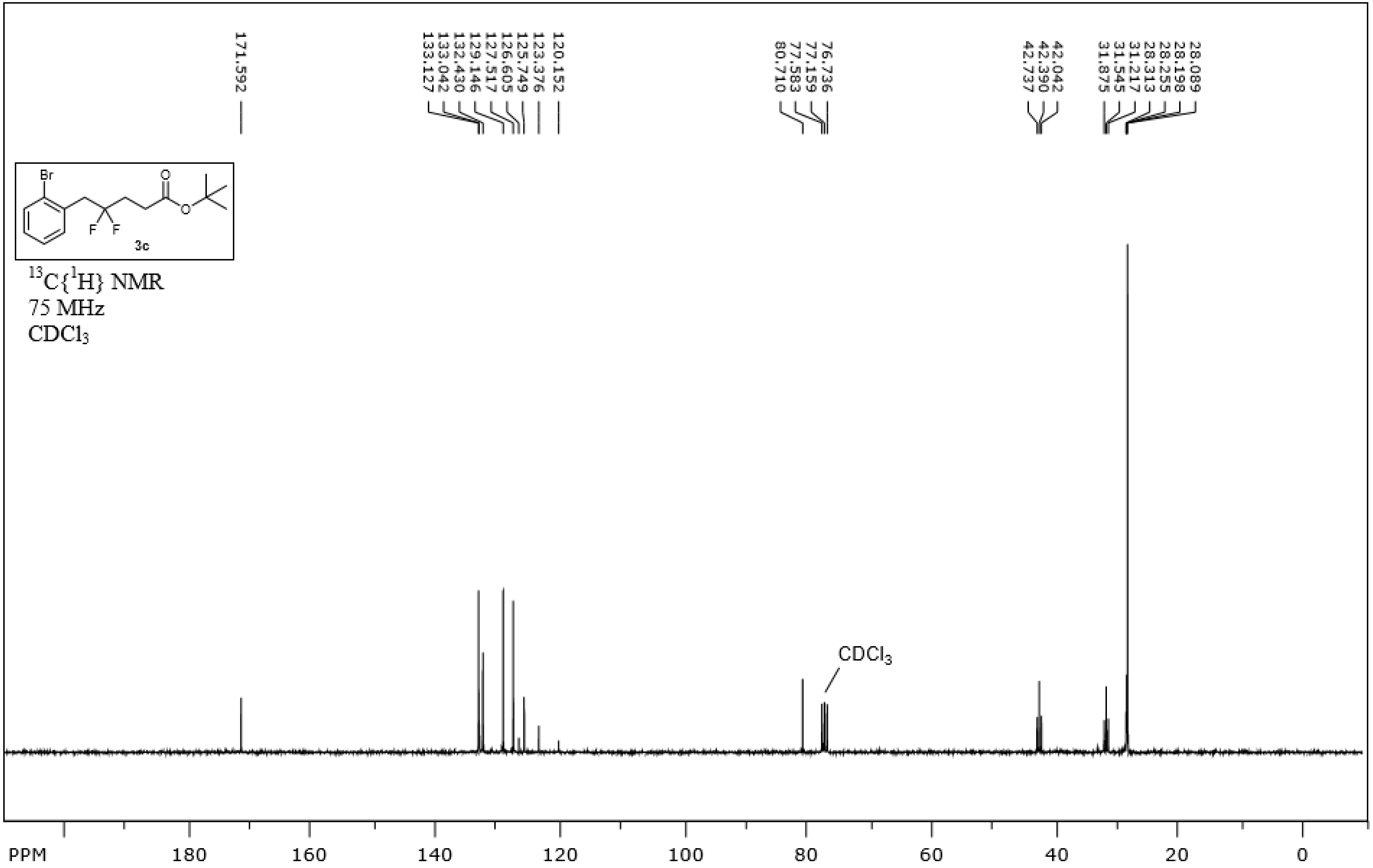

171.592
133.127
133.042
132.430
129.146
127.517
126.605
125.749
123.376
120.152
80.710
77.583
77.159
76.736
42.737
42.390
42.042
31.875
31.545
31.217
28.313
28.255
28.198
28.089
Br
O
O
F F
3c
$^{13}C\{^1H\}$ NMR
75 MHz
$CDCl_3$
$CDCl_3$
PPM
180
160
140
120
100
80
60
40
20
0

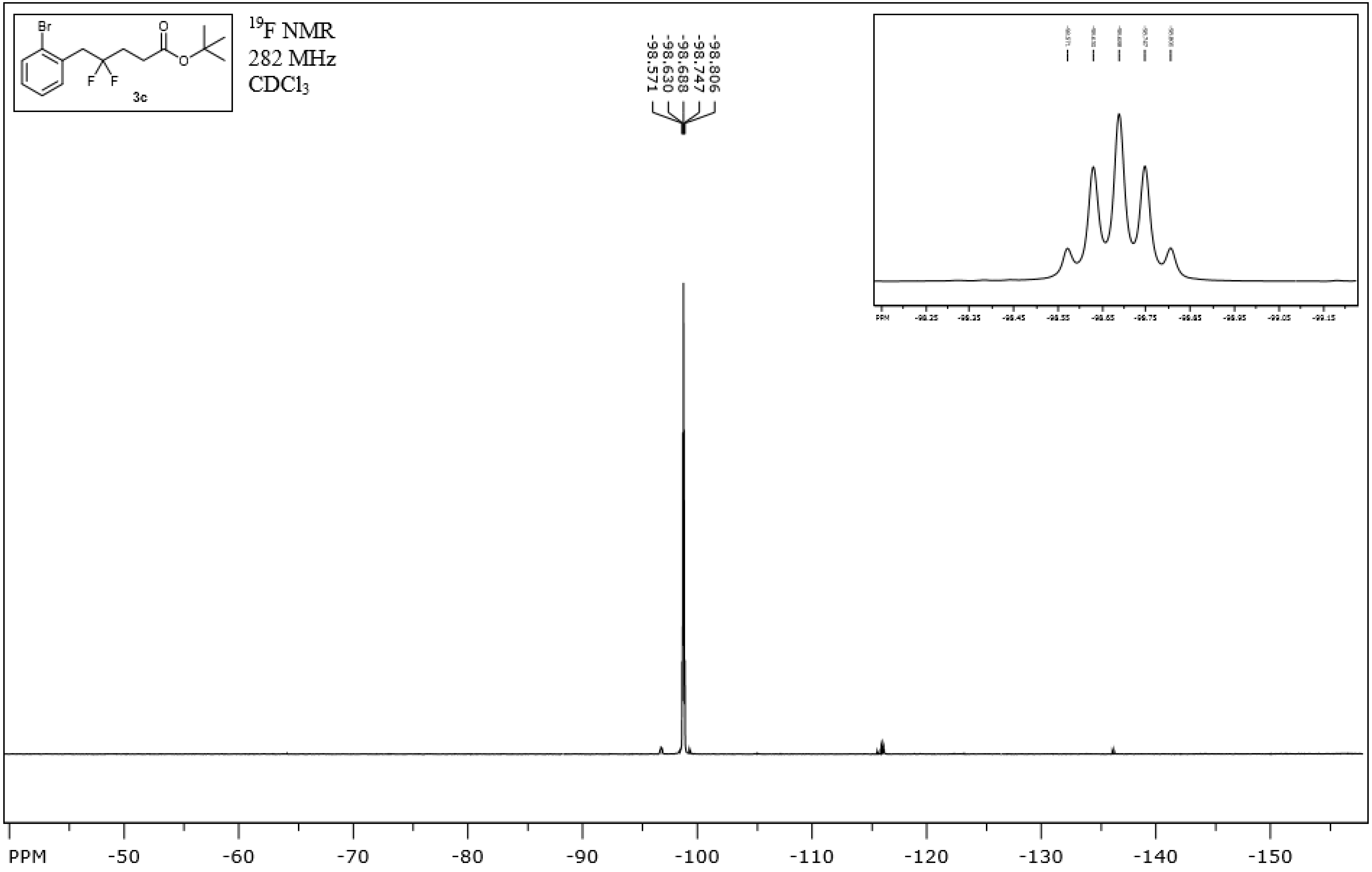
Br
O
O
F F
3c
19F NMR
282 MHz
CDCl3
-98.571
-98.630
-98.688
-98.747
-98.806
PPM
-50
-60
-70
-80
-90
-100
-110
-120
-130
-140
-150

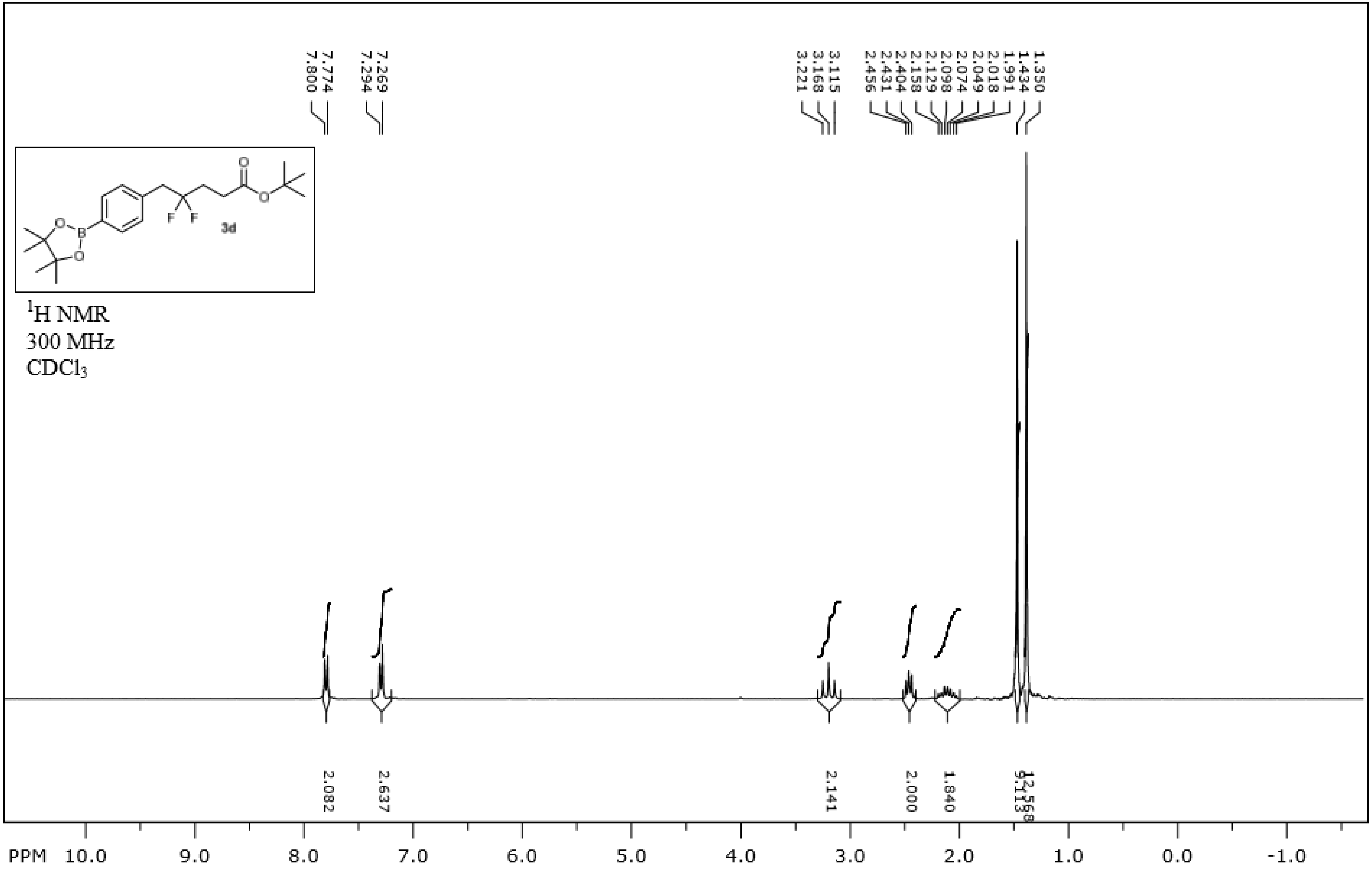
3d
^{1}H NMR
300 MHz
$CDCl_3$
7.800
7.774
7.294
7.269
3.221
3.168
3.115
2.456
2.431
2.404
2.158
2.129
2.098
2.074
2.049
2.018
1.991
1.434
1.350
2.082
2.637
2.141
2.000
1.840
9.113
12.568
PPM 10.0 9.0 8.0 7.0 6.0 5.0 4.0 3.0 2.0 1.0 0.0 -1.0

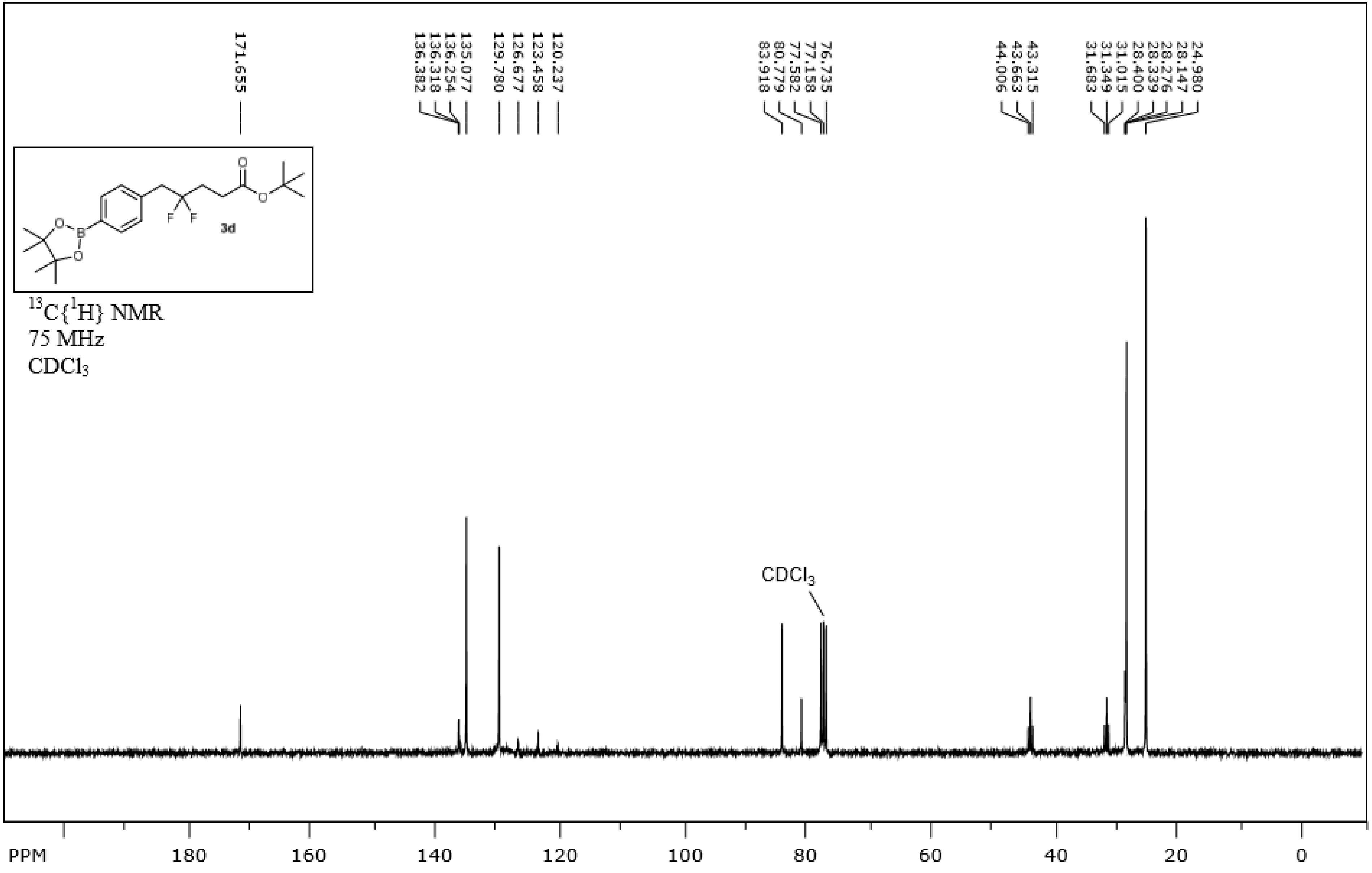
3d
$^{13}C\{^1H\}$ NMR
75 MHz
$CDCl_3$
171.655
136.382
136.318
136.254
135.077
129.780
126.677
123.458
120.237
83.918
80.779
77.582
77.158
76.735
44.006
43.663
43.315
31.683
31.349
31.015
28.400
28.339
28.276
28.147
24.980
$CDCl_3$
PPM
180
160
140
120
100
80
60
40
20
0

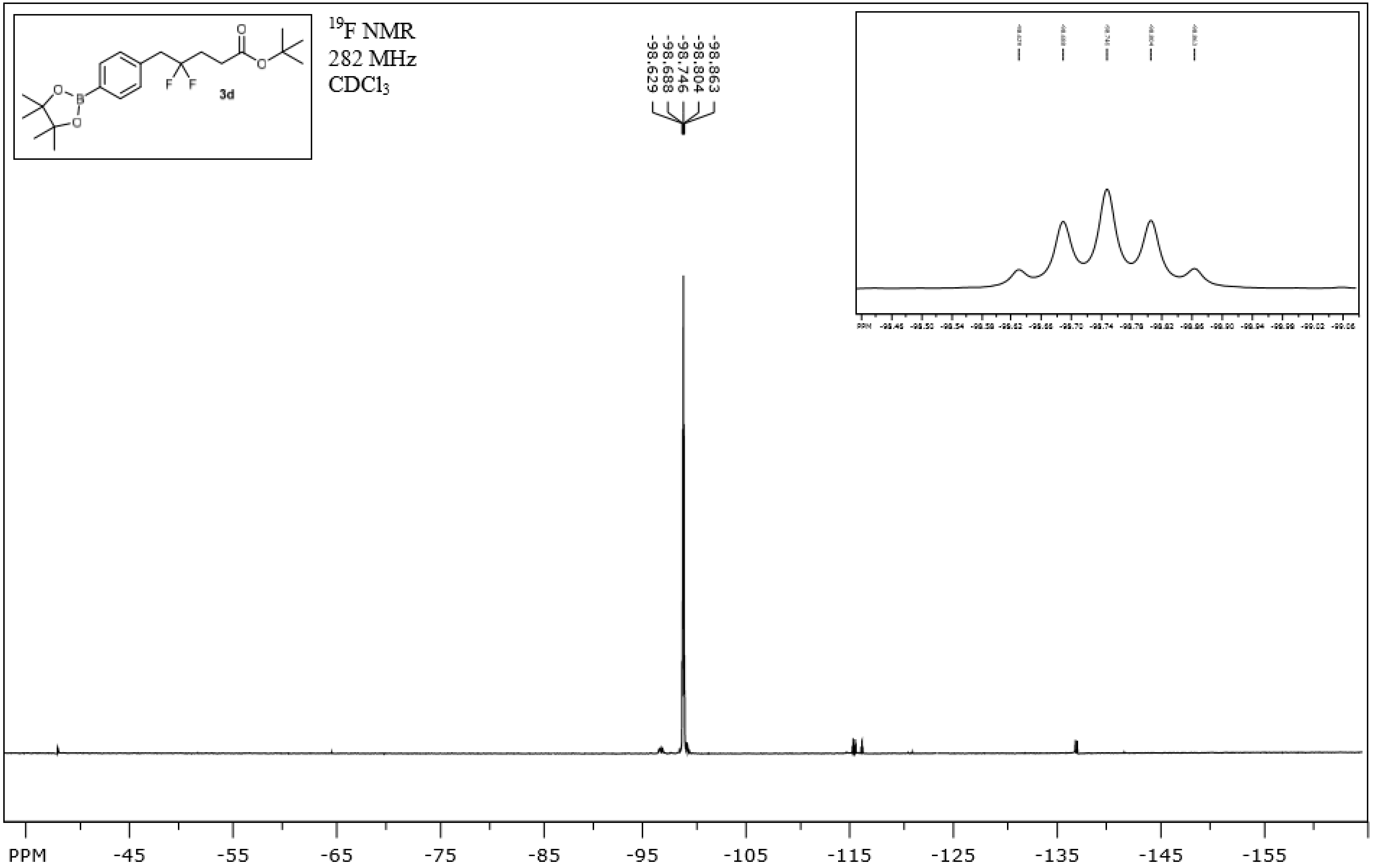
3d
^{19}F NMR
282 MHz
$CDCl_3$
-98.629
-98.688
-98.746
-98.804
-98.863
PPM -45 -55 -65 -75 -85 -95 -105 -115 -125 -135 -145 -155

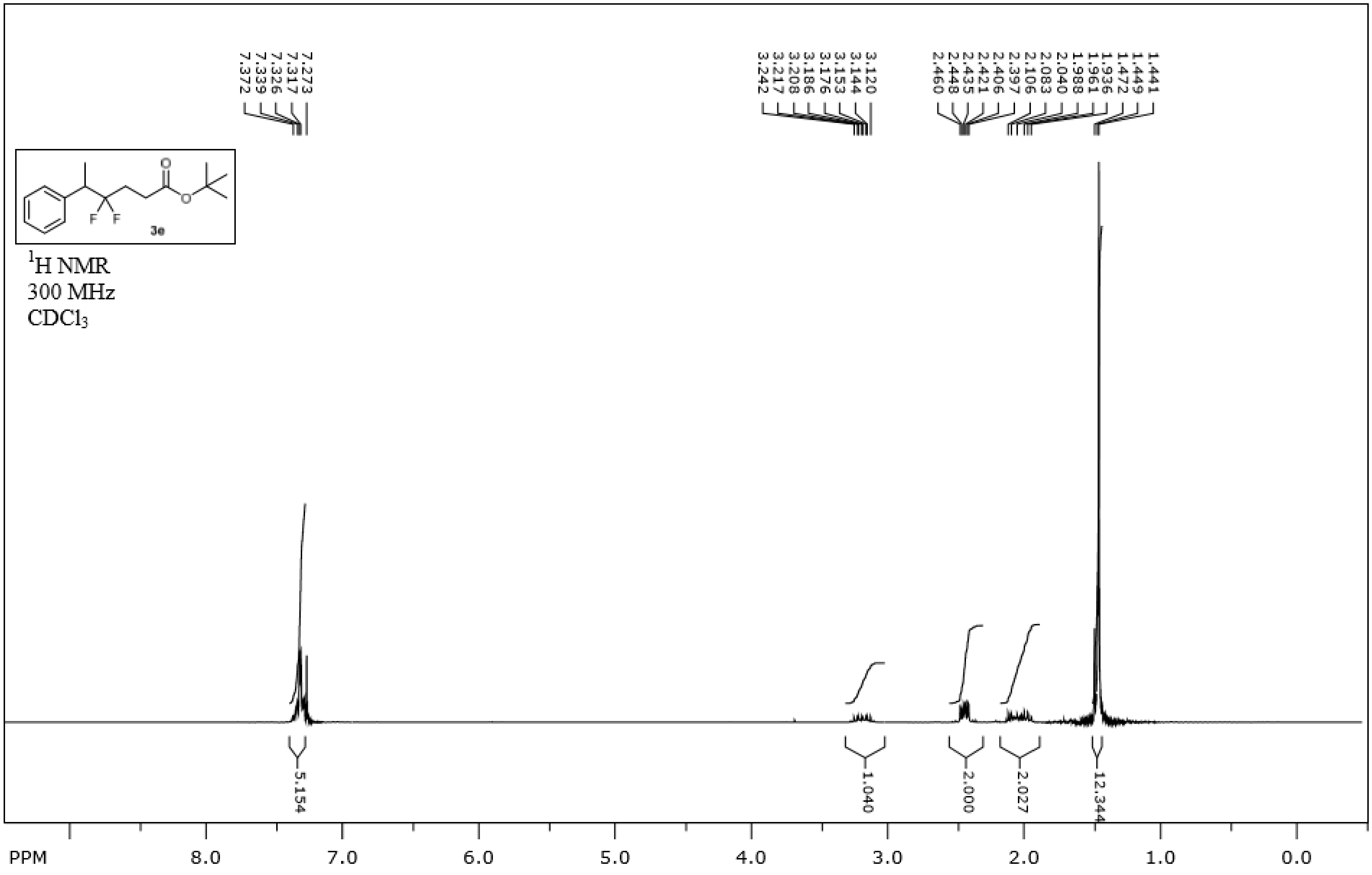
7.372
7.339
7.326
7.317
7.273
3.242
3.217
3.208
3.186
3.176
3.153
3.144
3.120
2.460
2.448
2.435
2.421
2.406
2.397
2.106
2.083
2.040
1.988
1.961
1.936
1.472
1.449
1.441
3e
^{1}H NMR
300 MHz
$CDCl_3$
5.154
1.040
2.000
2.027
12.344
PPM
8.0
7.0
6.0
5.0
4.0
3.0
2.0
1.0
0.0

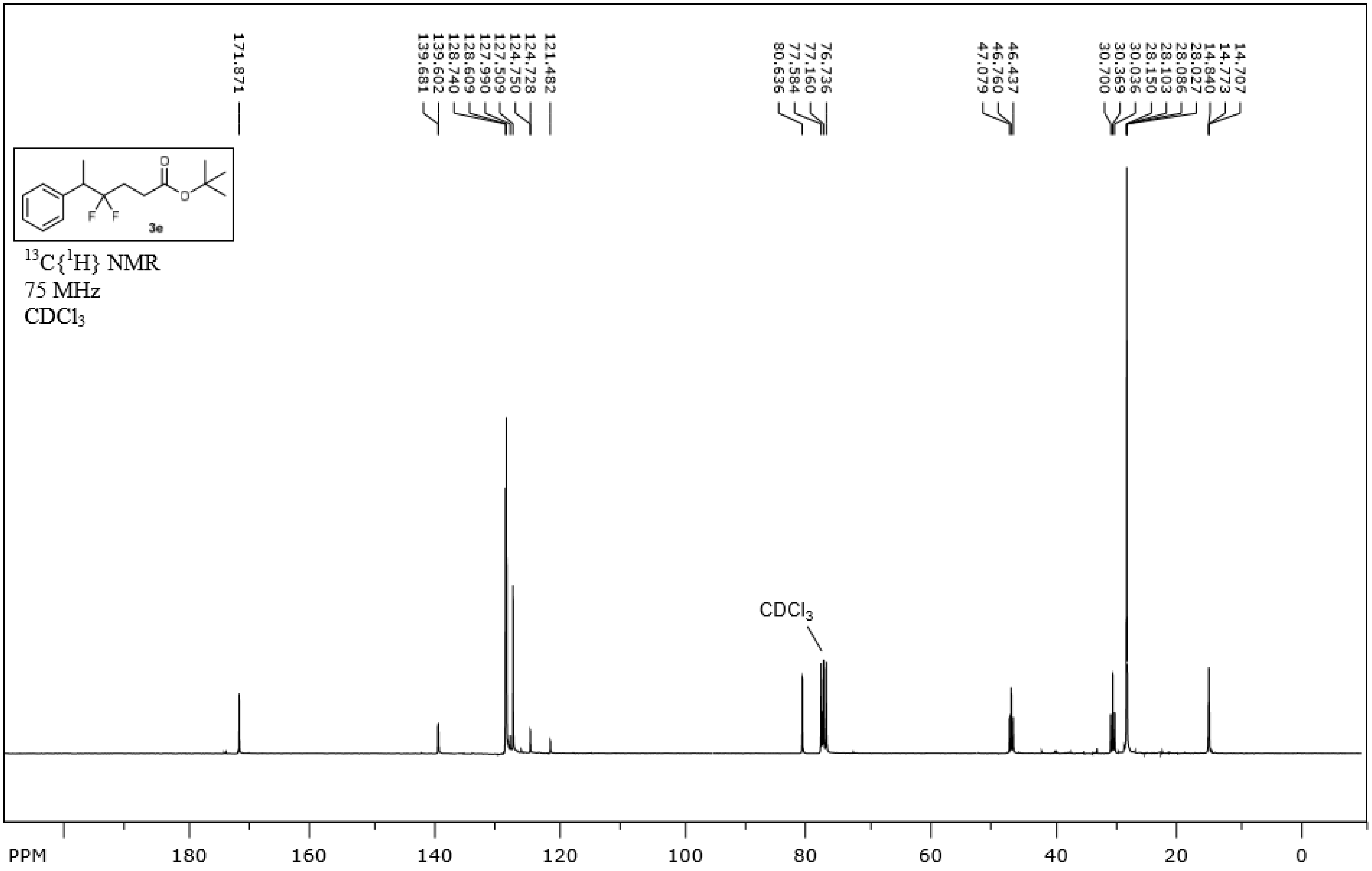
171.871
139.681
139.602
128.740
128.609
127.990
127.509
124.750
124.728
121.482
80.636
77.584
77.160
76.736
47.079
46.760
46.437
30.700
30.369
30.036
28.150
28.103
28.086
28.027
14.840
14.773
14.707
3e
$^{13}C\{^1H\}$ NMR
75 MHz
$CDCl_3$
$CDCl_3$
PPM
180
160
140
120
100
80
60
40
20
0

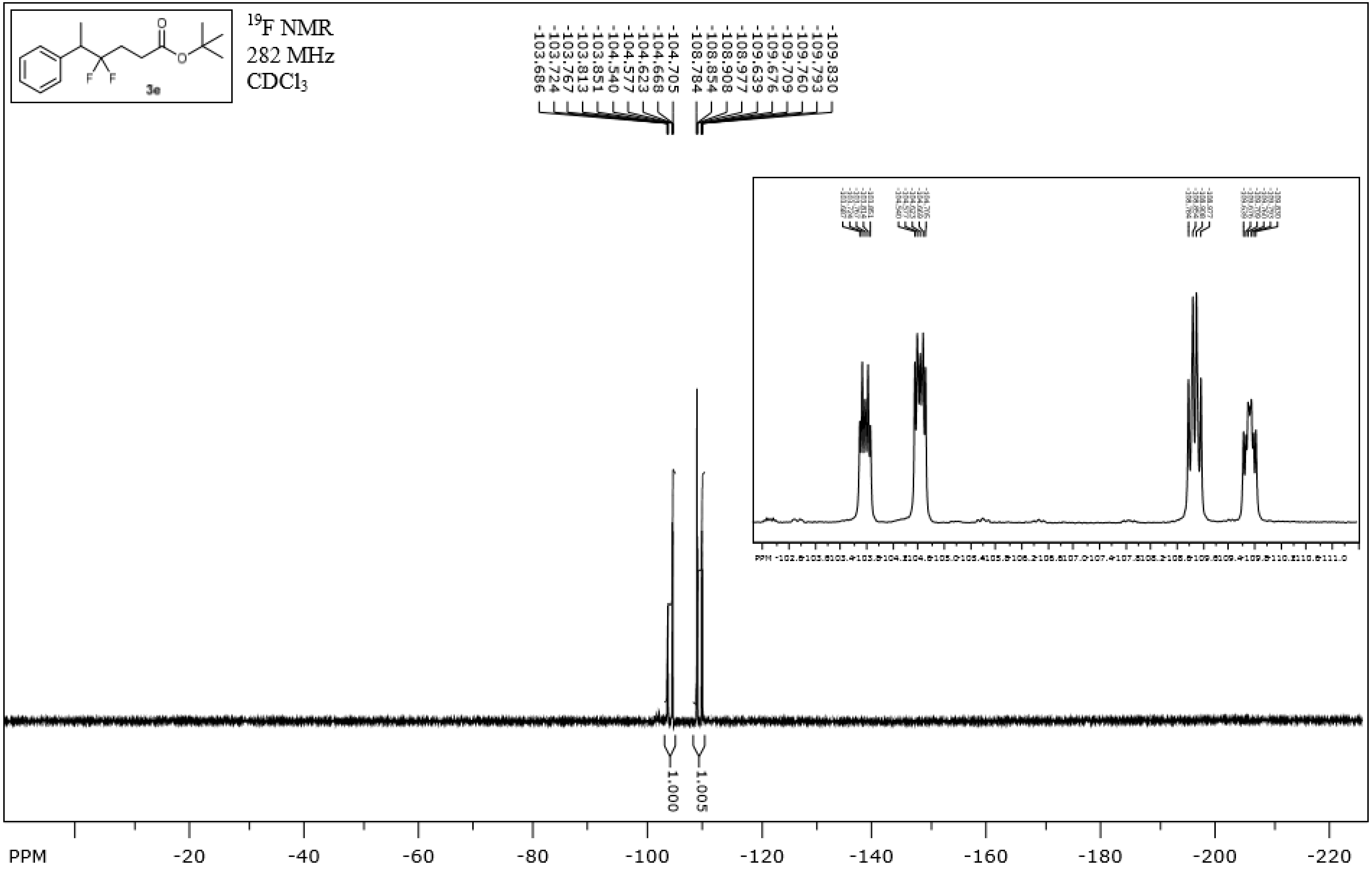

^{19}F NMR
282 MHz
CDCl$_3$
3e
-103.686
-103.724
-103.767
-103.813
-103.851
-104.540
-104.577
-104.623
-104.668
-104.705
-108.784
-108.854
-108.908
-108.977
-109.639
-109.676
-109.709
-109.760
-109.793
-109.830
1.000
1.005
PPM
-20
-40
-60
-80
-100
-120
-140
-160
-180
-200
-220

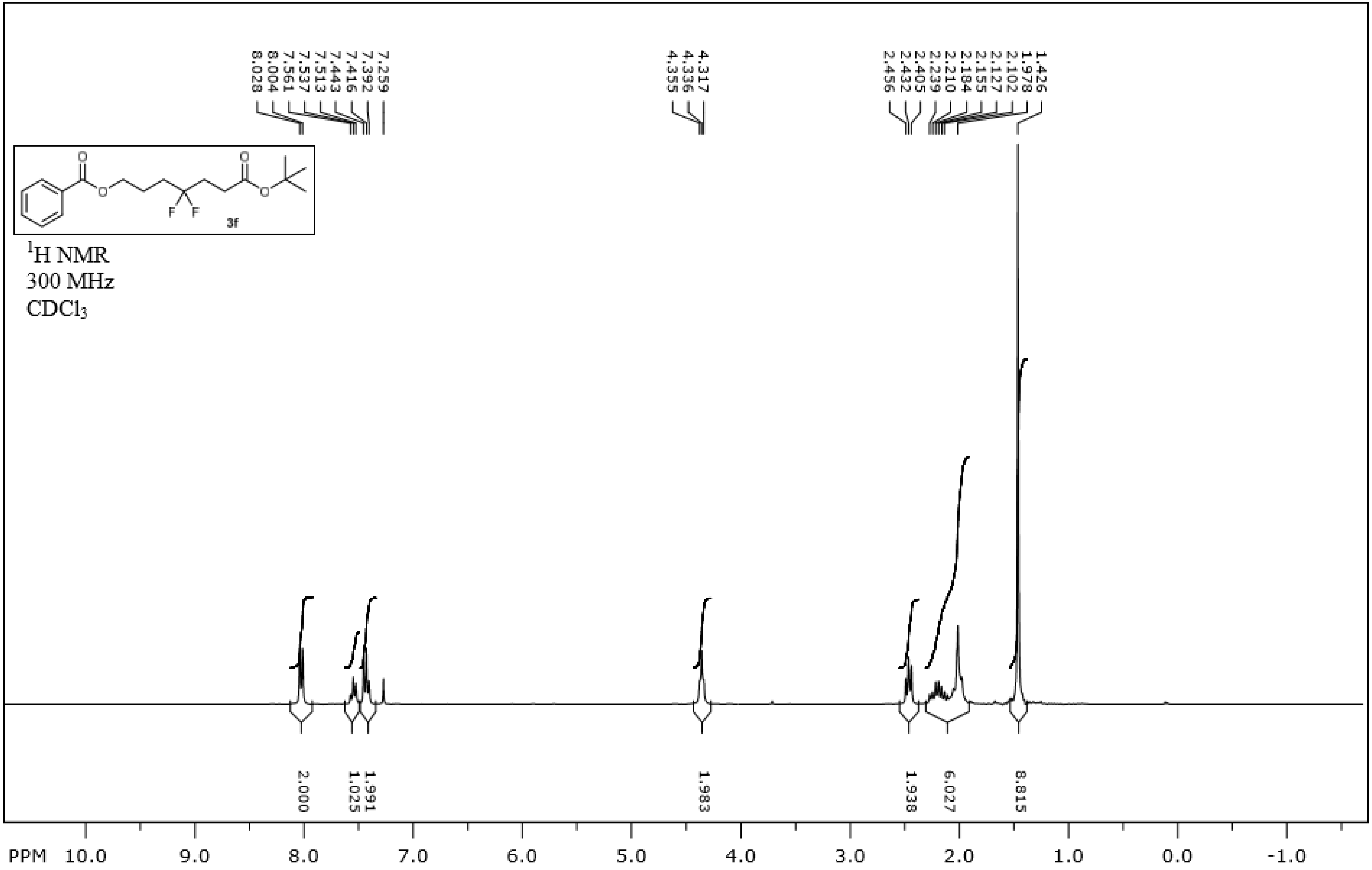
3f
1H NMR
300 MHz
CDCl3
8.028
8.004
7.561
7.537
7.513
7.443
7.416
7.392
7.259
4.355
4.336
4.317
2.456
2.432
2.405
2.239
2.210
2.184
2.155
2.127
2.102
1.978
1.426
2.000
1.025
1.991
1.983
1.938
6.027
8.815
PPM 10.0 9.0 8.0 7.0 6.0 5.0 4.0 3.0 2.0 1.0 0.0 -1.0

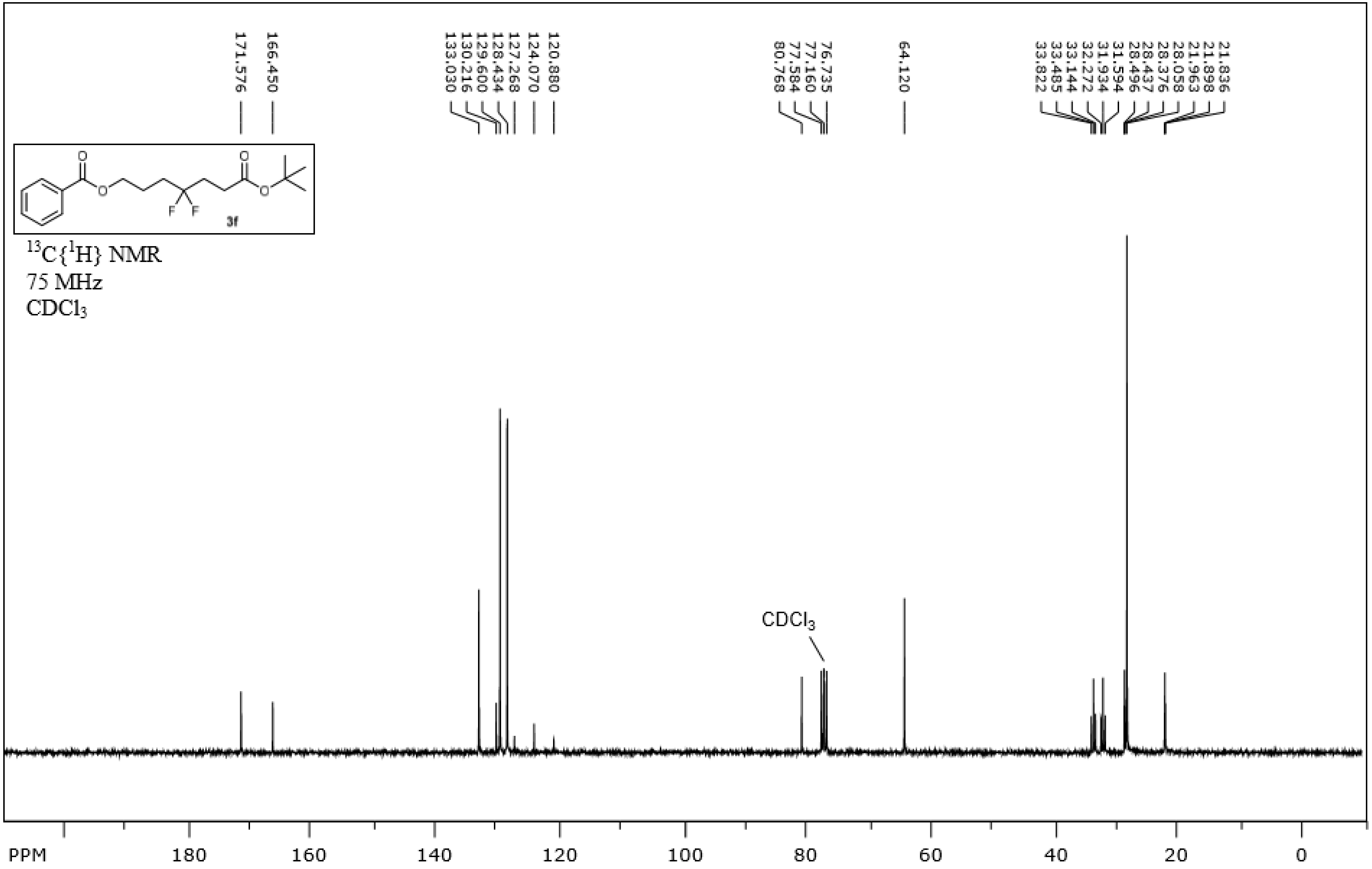
171.576
166.450
133.030
130.216
129.600
128.434
127.268
124.070
120.880
80.768
77.584
77.160
76.735
64.120
33.822
33.485
33.144
32.272
31.934
31.594
28.496
28.437
28.376
28.058
21.963
21.898
21.836
3f
$^{13}C\{^1H\}$ NMR
75 MHz
$CDCl_3$
$CDCl_3$
PPM
180
160
140
120
100
80
60
40
20
0

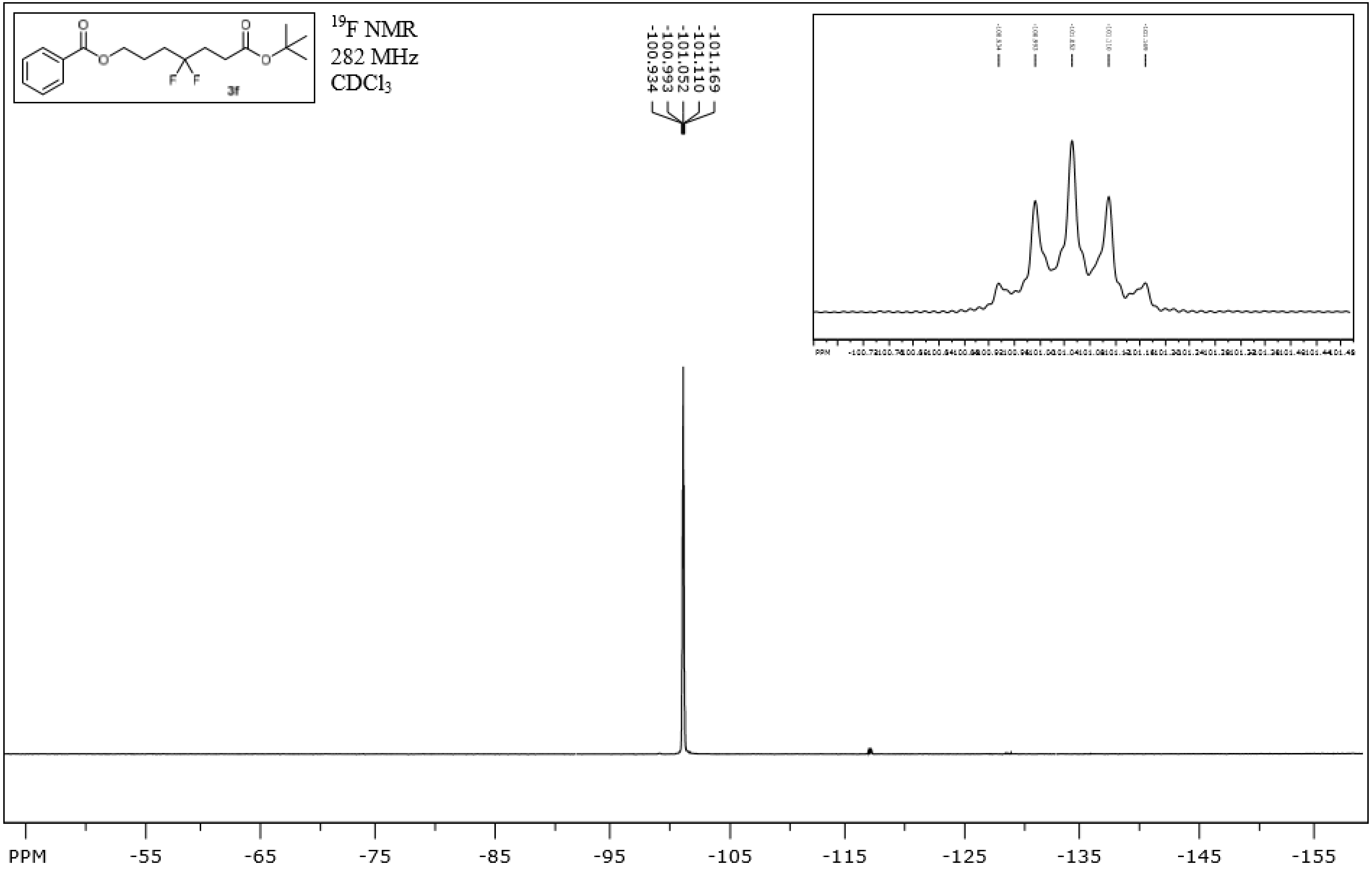
3f
19F NMR
282 MHz
CDCl3
-101.169
-101.110
-101.052
-100.993
-100.934
PPM
-55
-65
-75
-85
-95
-105
-115
-125
-135
-145
-155

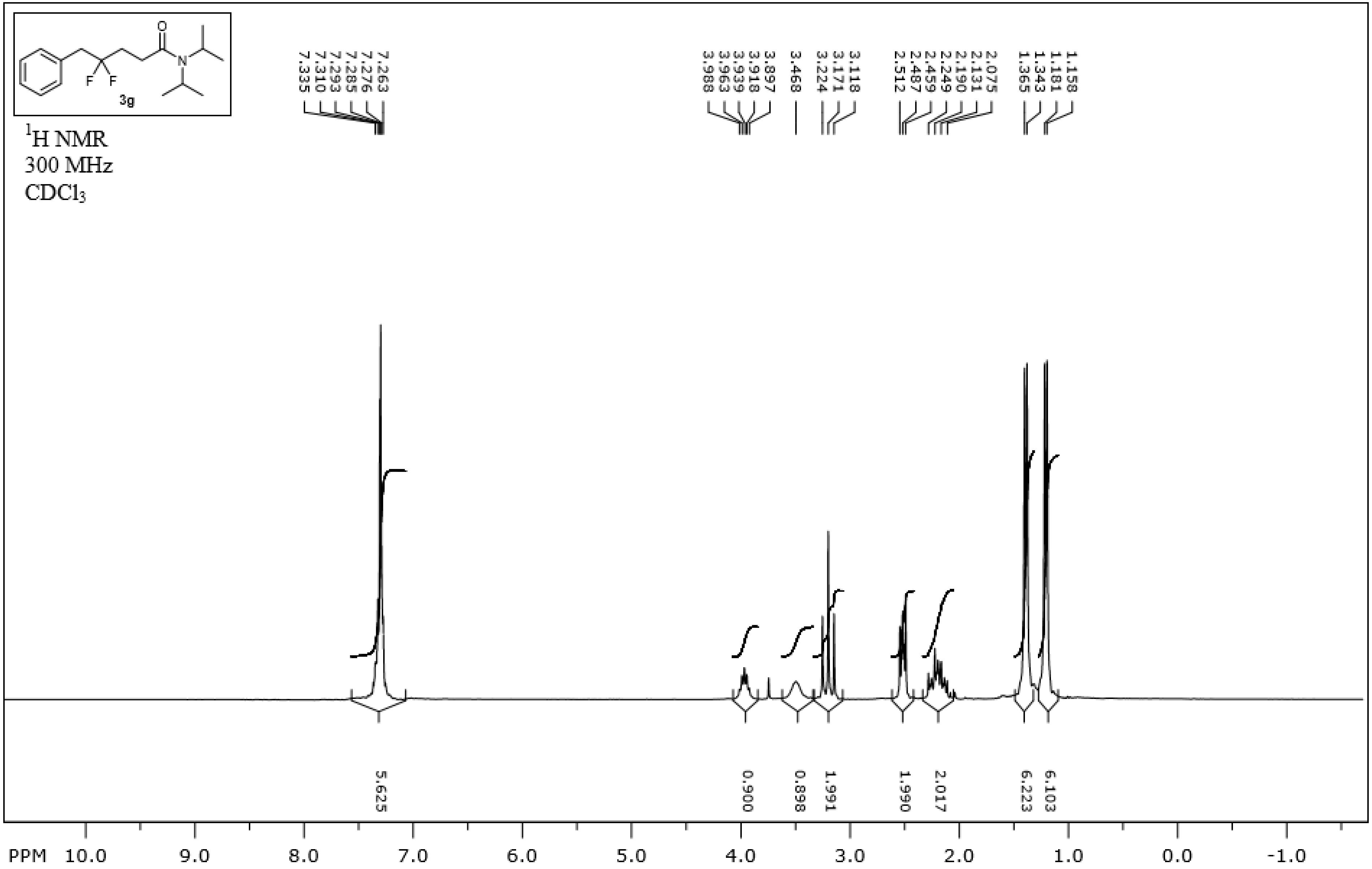

3g
1H NMR
300 MHz
CDCl3
7.335
7.310
7.293
7.285
7.276
7.263
3.988
3.963
3.939
3.918
3.897
3.468
3.224
3.171
3.118
2.512
2.487
2.459
2.249
2.190
2.131
2.075
1.365
1.343
1.181
1.158
5.625
0.900
0.898
1.991
1.990
2.017
6.223
6.103
PPM 10.0 9.0 8.0 7.0 6.0 5.0 4.0 3.0 2.0 1.0 0.0 -1.0

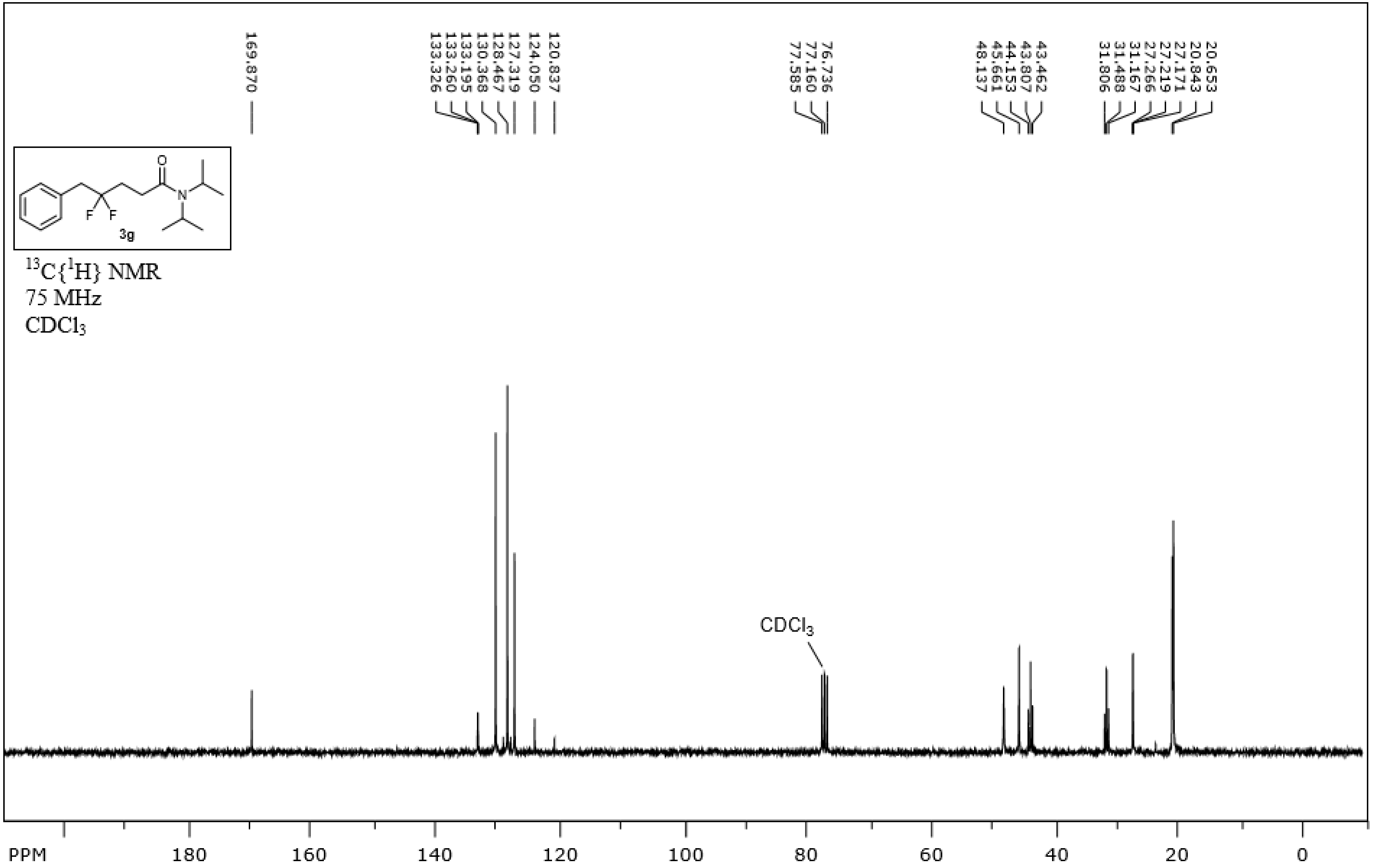
169.870
133.326
133.260
133.195
130.368
128.467
127.319
124.050
120.837
77.585
77.160
76.736
48.137
45.661
44.153
43.807
43.462
31.806
31.488
31.167
27.266
27.219
27.171
20.843
20.653
3g
$^{13}C\{^1H\}$ NMR
75 MHz
$CDCl_3$
$CDCl_3$
PPM
180
160
140
120
100
80
60
40
20
0

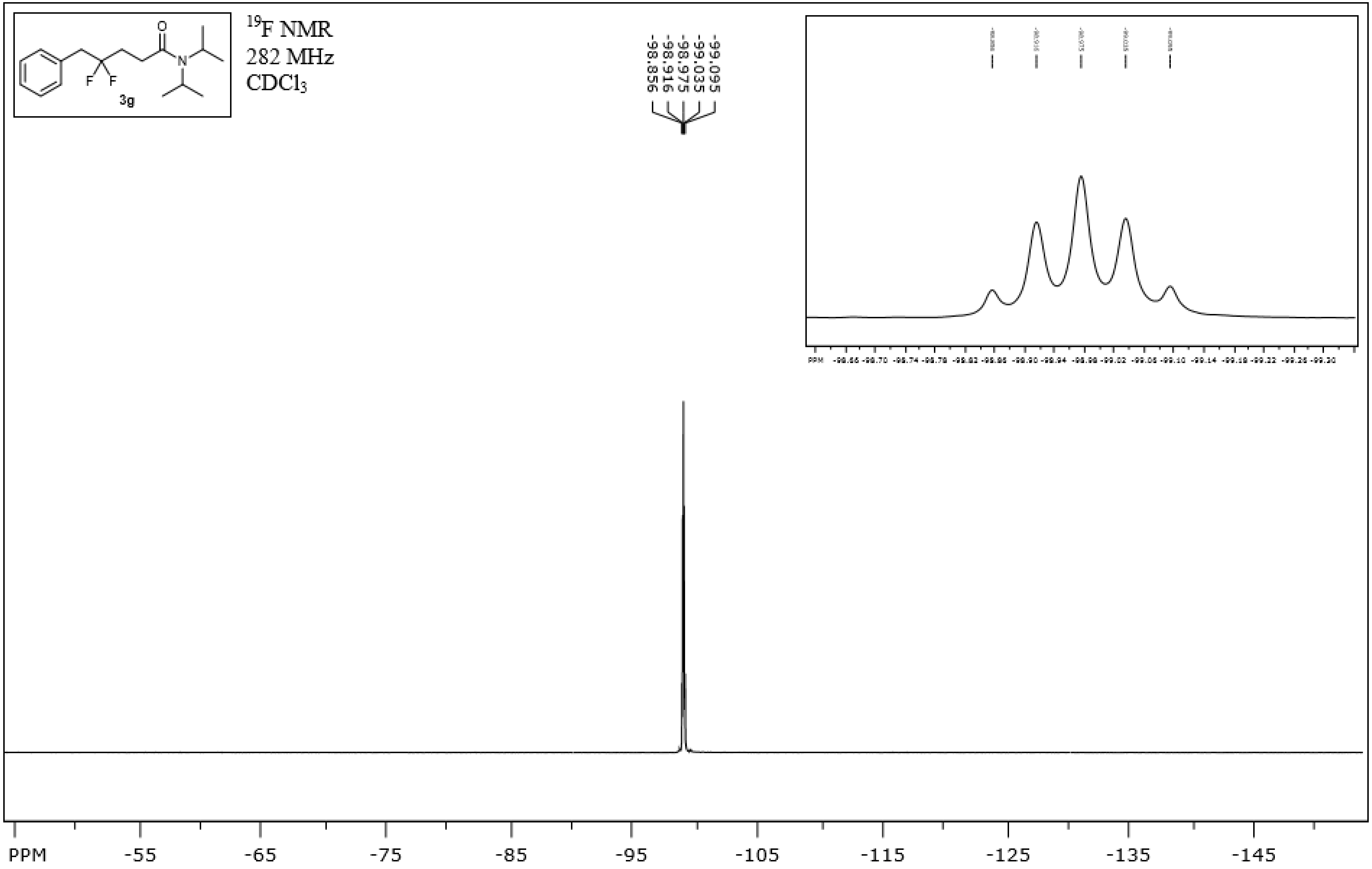

^{19}F NMR
282 MHz
$CDCl_3$
3g
F
F
O
N
-98.856
-98.916
-98.975
-99.035
-99.095
PPM
-55
-65
-75
-85
-95
-105
-115
-125
-135
-145

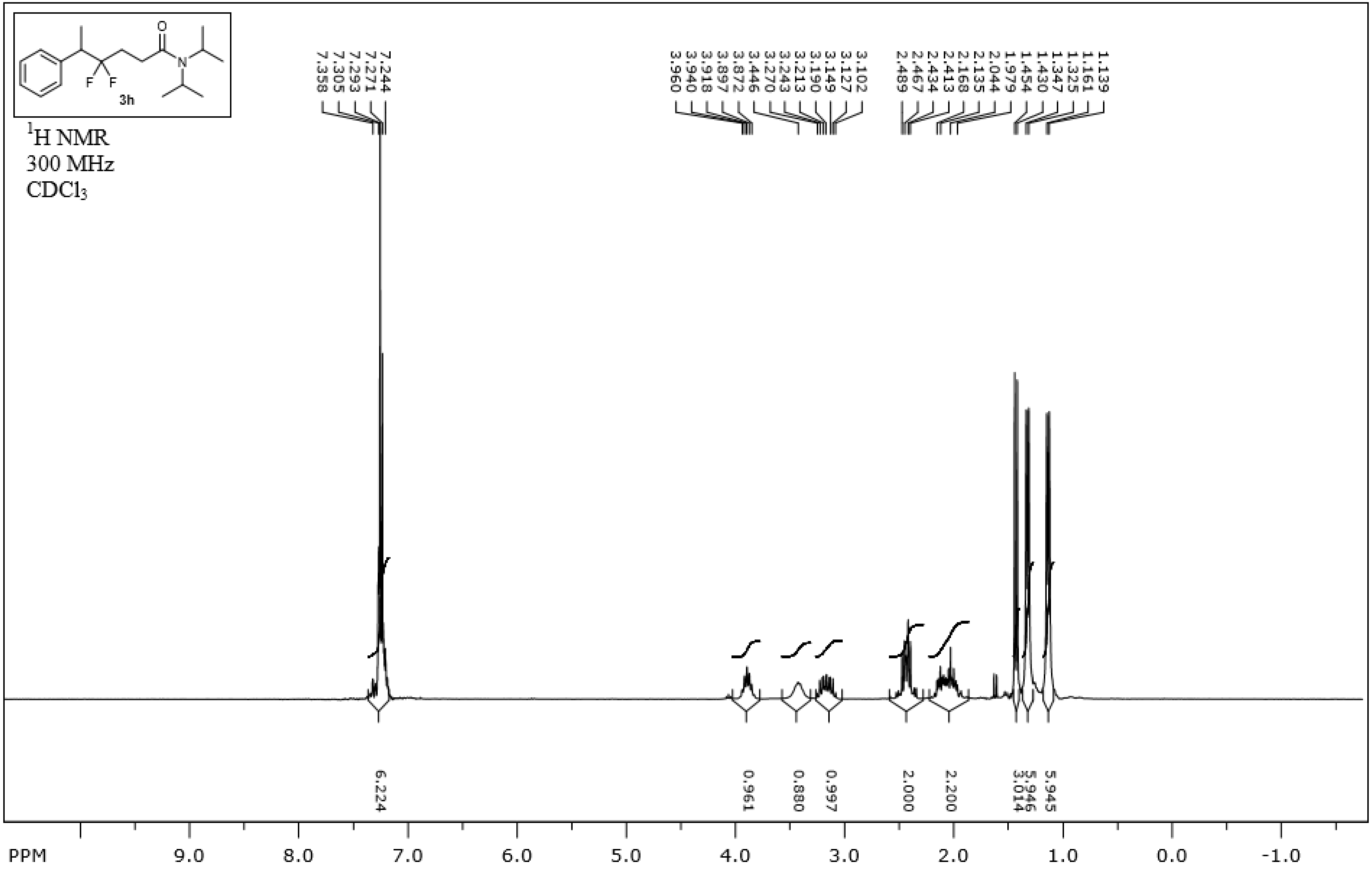
3h
1H NMR
300 MHz
$CDCl_3$
7.358
7.305
7.293
7.271
7.244
3.960
3.940
3.918
3.897
3.872
3.446
3.270
3.243
3.213
3.190
3.149
3.127
3.102
2.489
2.467
2.434
2.413
2.168
2.135
2.044
1.979
1.454
1.430
1.347
1.325
1.161
1.139
6.224
0.961
0.880
0.997
2.000
2.200
3.014
5.946
5.945
PPM
9.0
8.0
7.0
6.0
5.0
4.0
3.0
2.0
1.0
0.0
-1.0

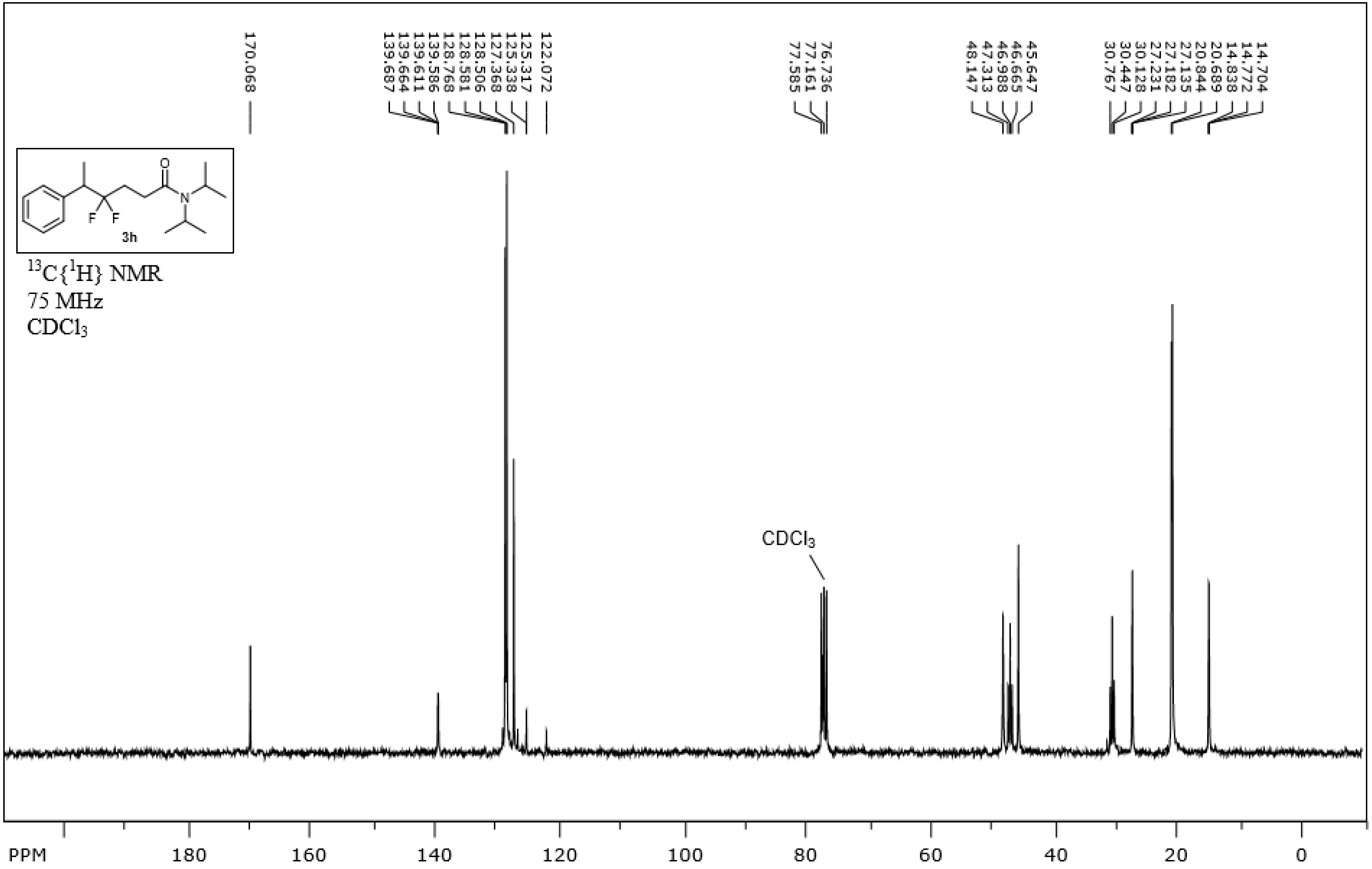
170.068
139.687
139.664
139.611
139.586
128.768
128.581
128.506
127.368
125.338
125.317
122.072
77.585
77.161
76.736
48.147
47.313
46.988
46.665
45.647
30.767
30.447
30.128
27.231
27.182
27.135
20.844
20.689
14.838
14.772
14.704
3h
13C{1H} NMR
75 MHz
CDCl3
CDCl3
PPM
180
160
140
120
100
80
60
40
20
0

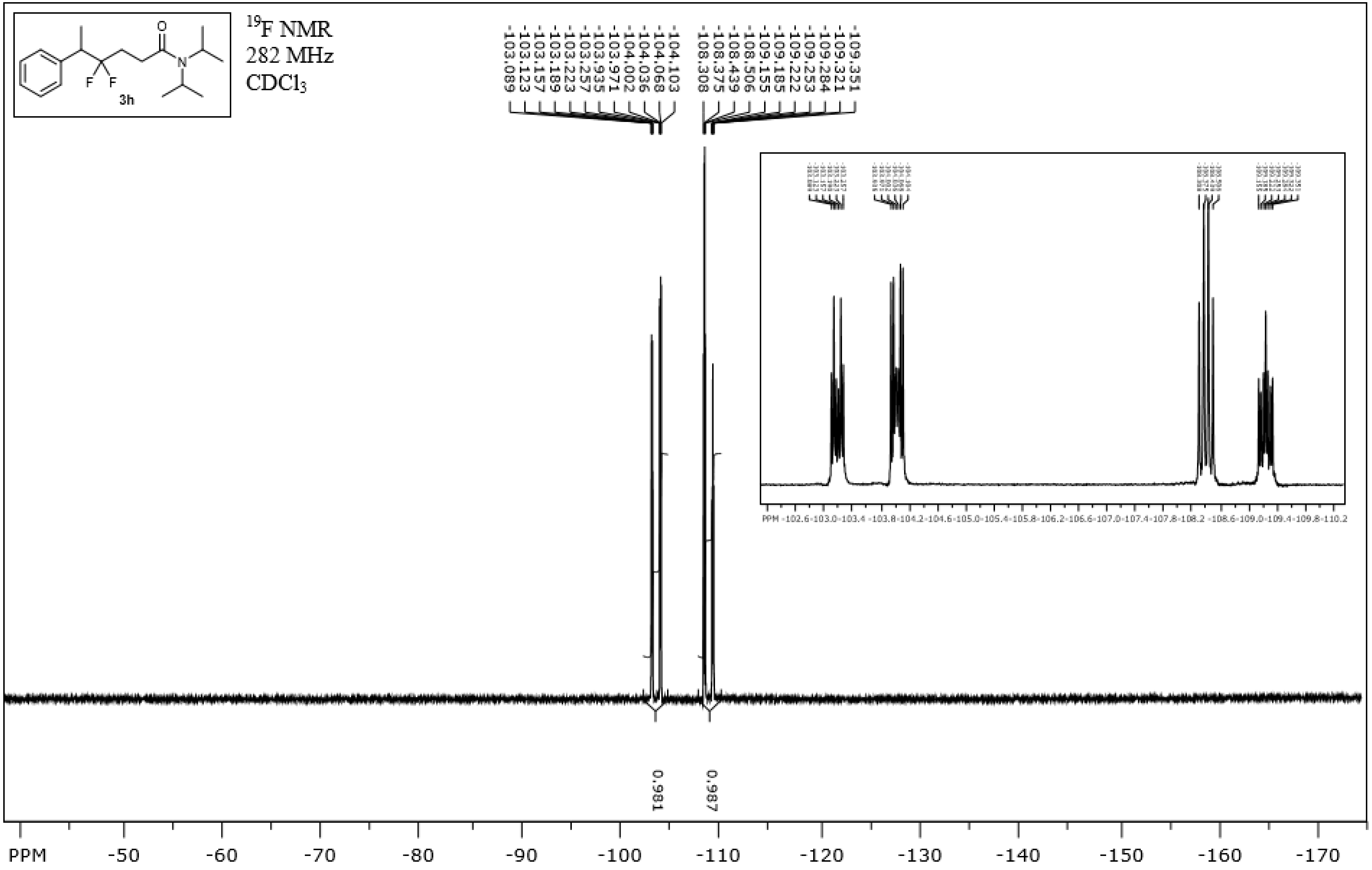
3h
19F NMR
282 MHz
CDCl3
-103.089
-103.123
-103.157
-103.189
-103.223
-103.257
-103.935
-103.971
-104.002
-104.036
-104.068
-104.103
-108.308
-108.375
-108.439
-108.506
-109.155
-109.185
-109.222
-109.253
-109.284
-109.321
-109.351
0.981
0.987
PPM -50 -60 -70 -80 -90 -100 -110 -120 -130 -140 -150 -160 -170

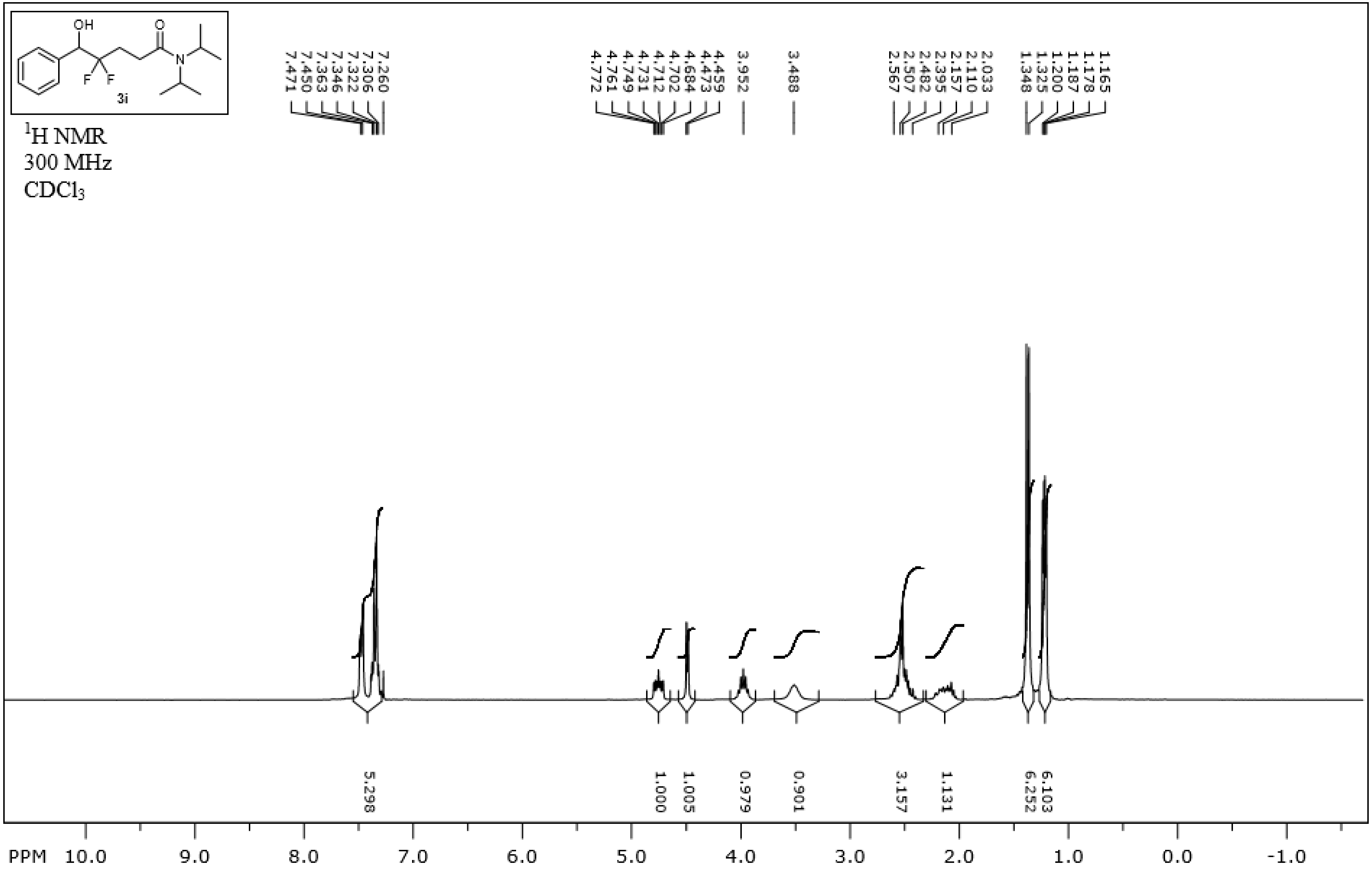
OH
O
N
F F
3i
1H NMR
300 MHz
CDCl3
7.471 7.450 7.363 7.346 7.322 7.306 7.260
4.772 4.761 4.749 4.731 4.712 4.702 4.684 4.473 4.459
3.952
3.488
2.567 2.507 2.482 2.395 2.157 2.110 2.033
1.348 1.325 1.200 1.187 1.178 1.165
5.298
1.000
1.005
0.979
0.901
3.157
1.131
6.252
6.103
PPM 10.0 9.0 8.0 7.0 6.0 5.0 4.0 3.0 2.0 1.0 0.0 -1.0

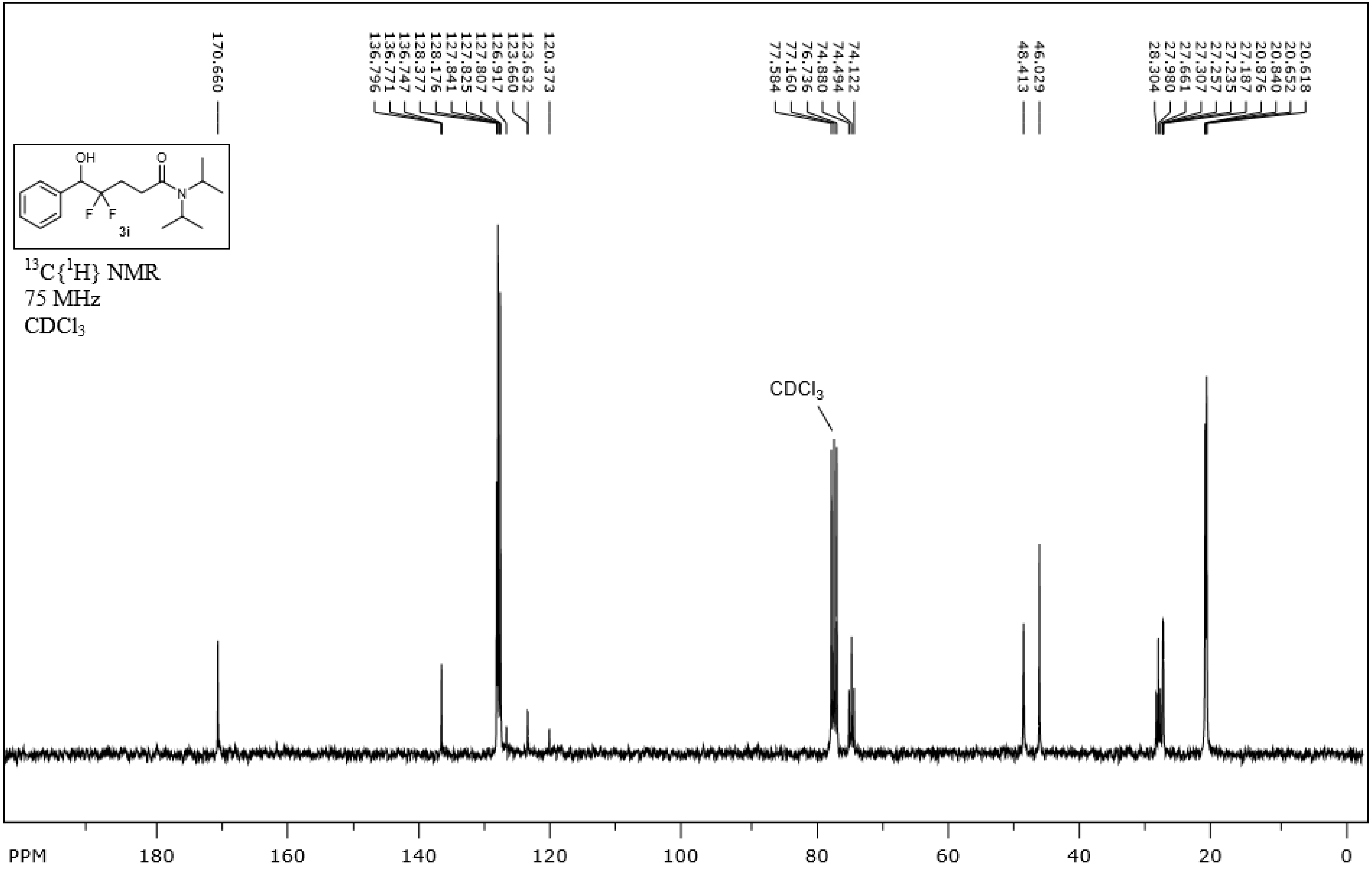
170.660
136.796
136.771
136.747
128.377
128.176
127.841
127.825
127.807
126.917
123.660
123.632
120.373
77.584
77.160
76.736
74.880
74.494
74.122
48.413
46.029
28.304
27.980
27.661
27.307
27.257
27.235
27.187
20.876
20.840
20.652
20.618
OH
O
N
F
F
3i
$^{13}C\{^1H\}$ NMR
75 MHz
$CDCl_3$
$CDCl_3$
PPM
180
160
140
120
100
80
60
40
20
0

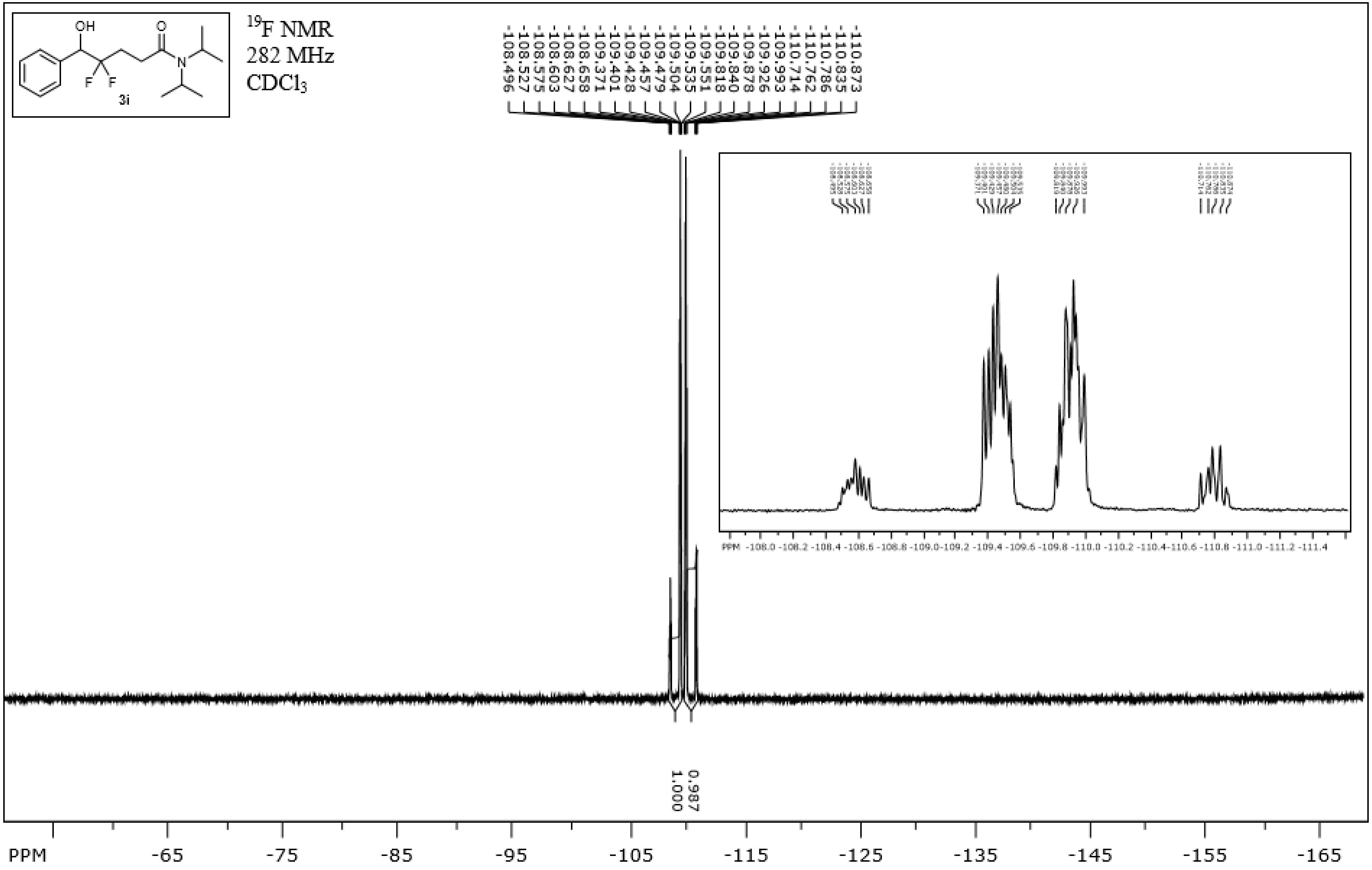
OH
O
N
F F
3i
^{19}F NMR
282 MHz
$CDCl_3$
-108.496
-108.527
-108.575
-108.603
-108.627
-108.658
-109.371
-109.401
-109.428
-109.457
-109.479
-109.504
-109.535
-109.551
-109.818
-109.840
-109.878
-109.926
-109.993
-110.714
-110.762
-110.786
-110.835
-110.873
1.000
0.987
PPM -65 -75 -85 -95 -105 -115 -125 -135 -145 -155 -165
PPM -108.0 -108.2 -108.4 -108.6 -108.8 -109.0 -109.2 -109.4 -109.6 -109.8 -110.0 -110.2 -110.4 -110.6 -110.8 -111.0 -111.2 -111.4

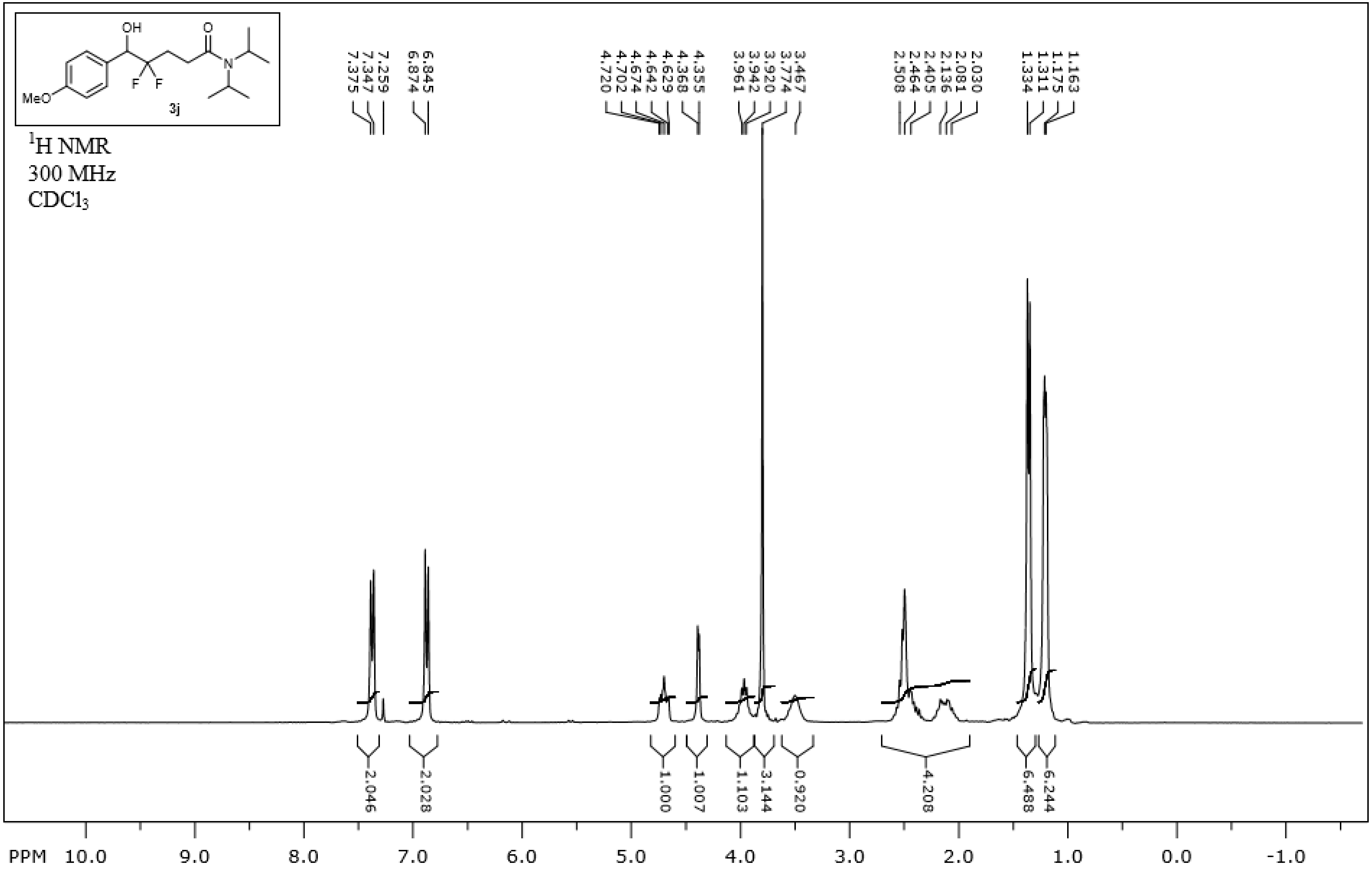
OH
O
N
F F
MeO
3j
^{1}H NMR
300 MHz
$CDCl_3$
7.375 7.347 7.259 6.874 6.845
4.720 4.702 4.674 4.642 4.629 4.368 4.355 3.961 3.942 3.920 3.774 3.467
2.508 2.464 2.405 2.136 2.081 2.030 1.334 1.311 1.175 1.163
2.046 2.028 1.000 1.007 1.103 3.144 0.920 4.208 6.488 6.244
PPM 10.0 9.0 8.0 7.0 6.0 5.0 4.0 3.0 2.0 1.0 0.0 -1.0

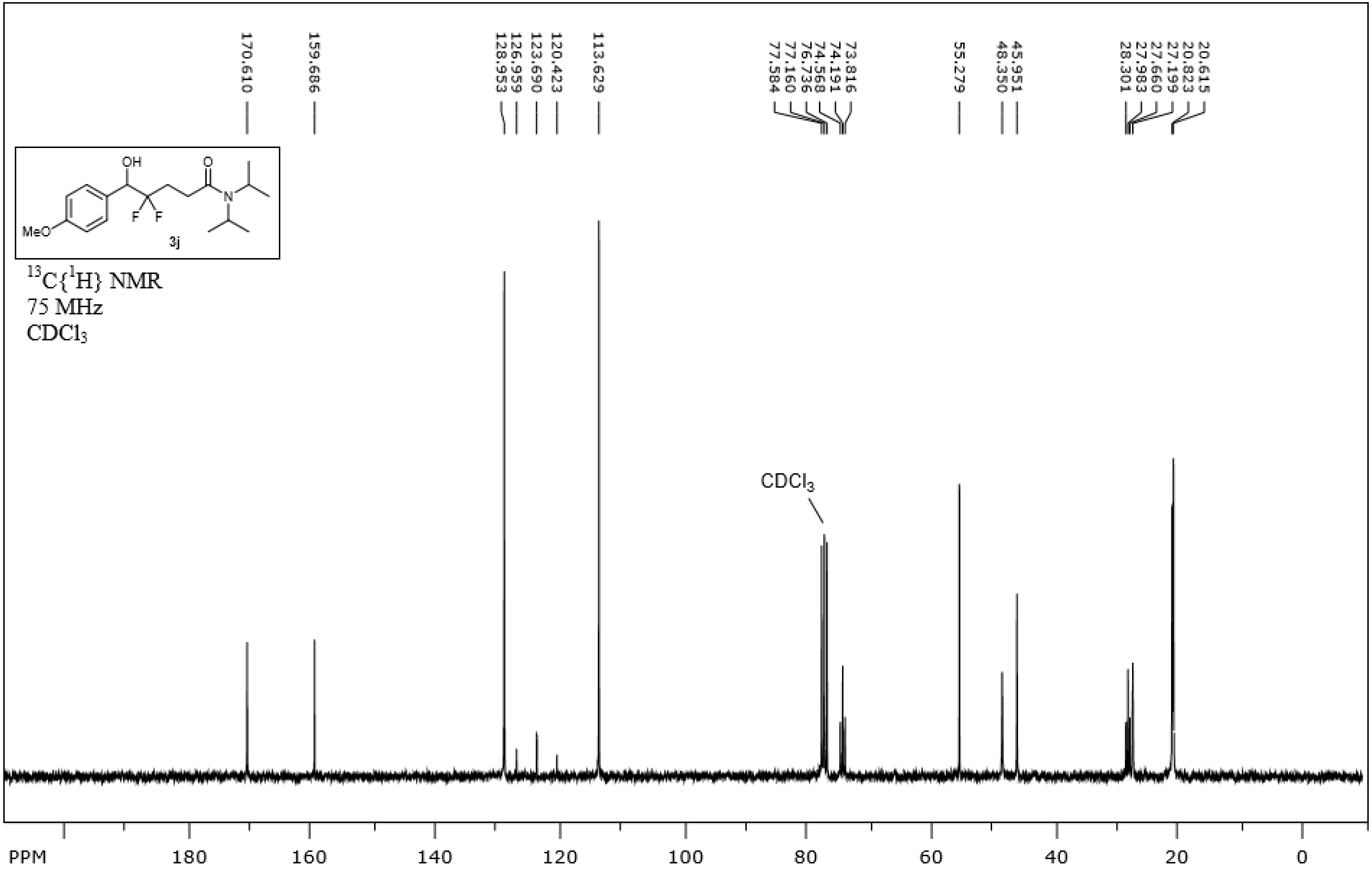
170.610
159.686
128.953
126.959
123.690
120.423
113.629
77.584
77.160
76.736
74.568
74.191
73.816
55.279
48.350
45.951
28.301
27.983
27.660
27.199
20.823
20.615
OH
O
N
F
F
MeO
3j
$^{13}C\{^1H\}$ NMR
75 MHz
$CDCl_3$
$CDCl_3$
PPM
180
160
140
120
100
80
60
40
20
0

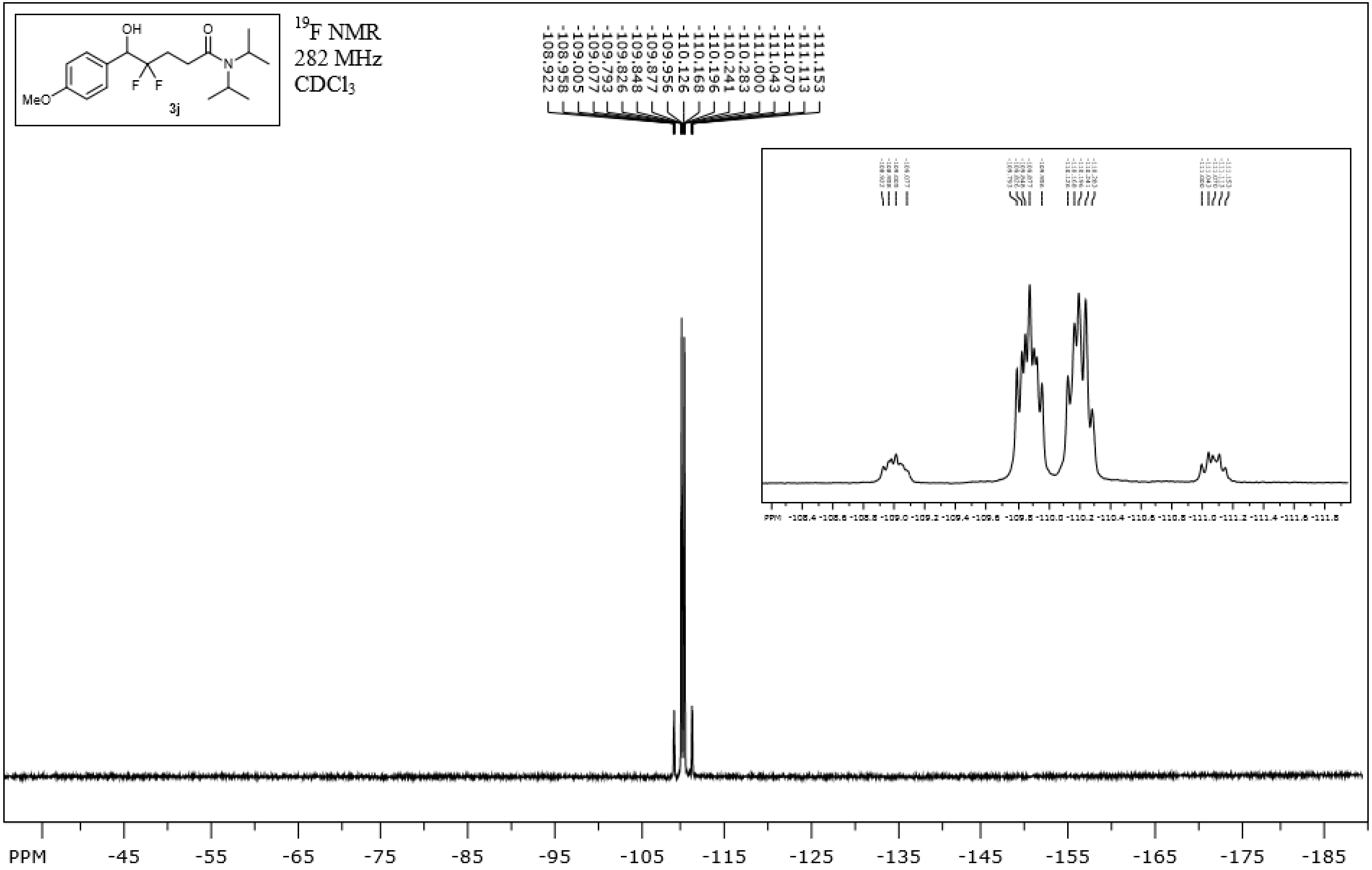
OH
O
N
F F
MeO
3j
19F NMR
282 MHz
CDCl3
-108.922
-108.958
-109.005
-109.077
-109.793
-109.826
-109.848
-109.877
-109.956
-110.126
-110.168
-110.196
-110.241
-110.283
-111.000
-111.043
-111.070
-111.113
-111.153
PPM -45 -55 -65 -75 -85 -95 -105 -115 -125 -135 -145 -155 -165 -175 -185

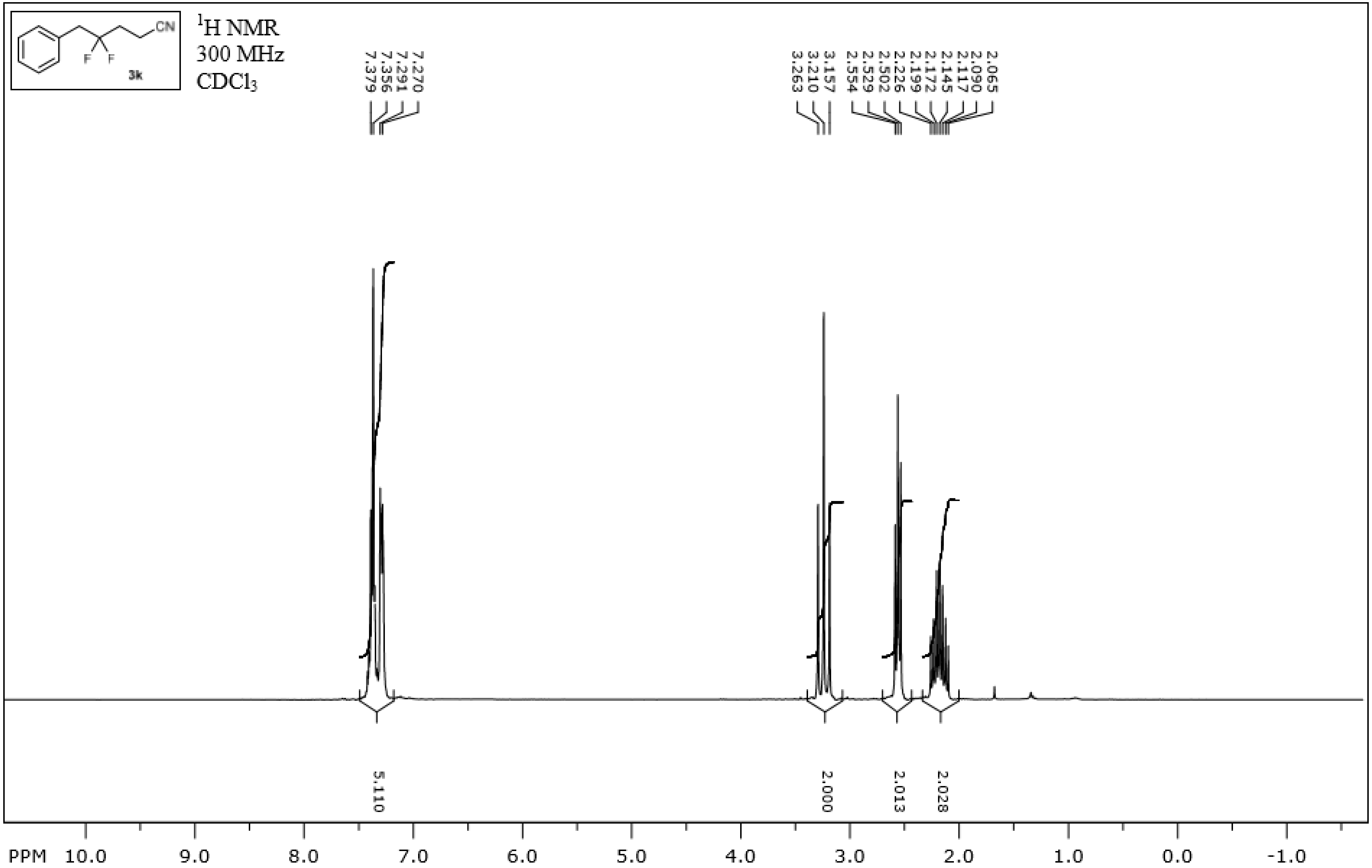

CN
F F
3k
^{1}H NMR
300 MHz
$CDCl_3$
7.379 7.356 7.291 7.270
3.263 3.210 3.157 2.554 2.529 2.502 2.226 2.199 2.172 2.145 2.117 2.090 2.065
5.110
2.000
2.013
2.028
PPM 10.0 9.0 8.0 7.0 6.0 5.0 4.0 3.0 2.0 1.0 0.0 -1.0

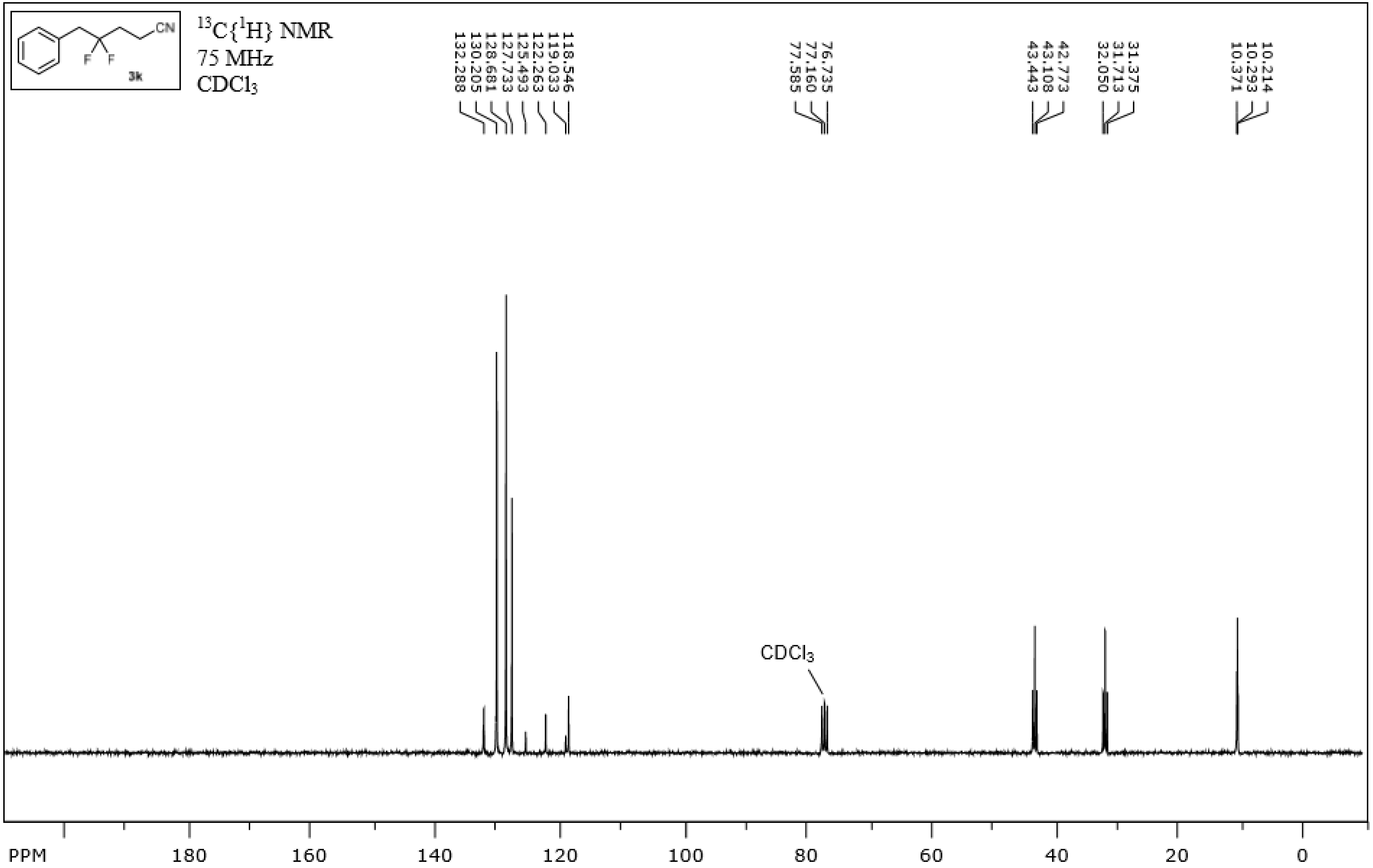

3k
F F
CN
$^{13}C\{^{1}H\}$ NMR
75 MHz
$CDCl_3$
132.288
130.205
128.681
127.733
125.493
122.263
119.033
118.546
77.585
77.160
76.735
43.443
43.108
42.773
32.050
31.713
31.375
10.371
10.293
10.214
$CDCl_3$
PPM
180
160
140
120
100
80
60
40
20
0

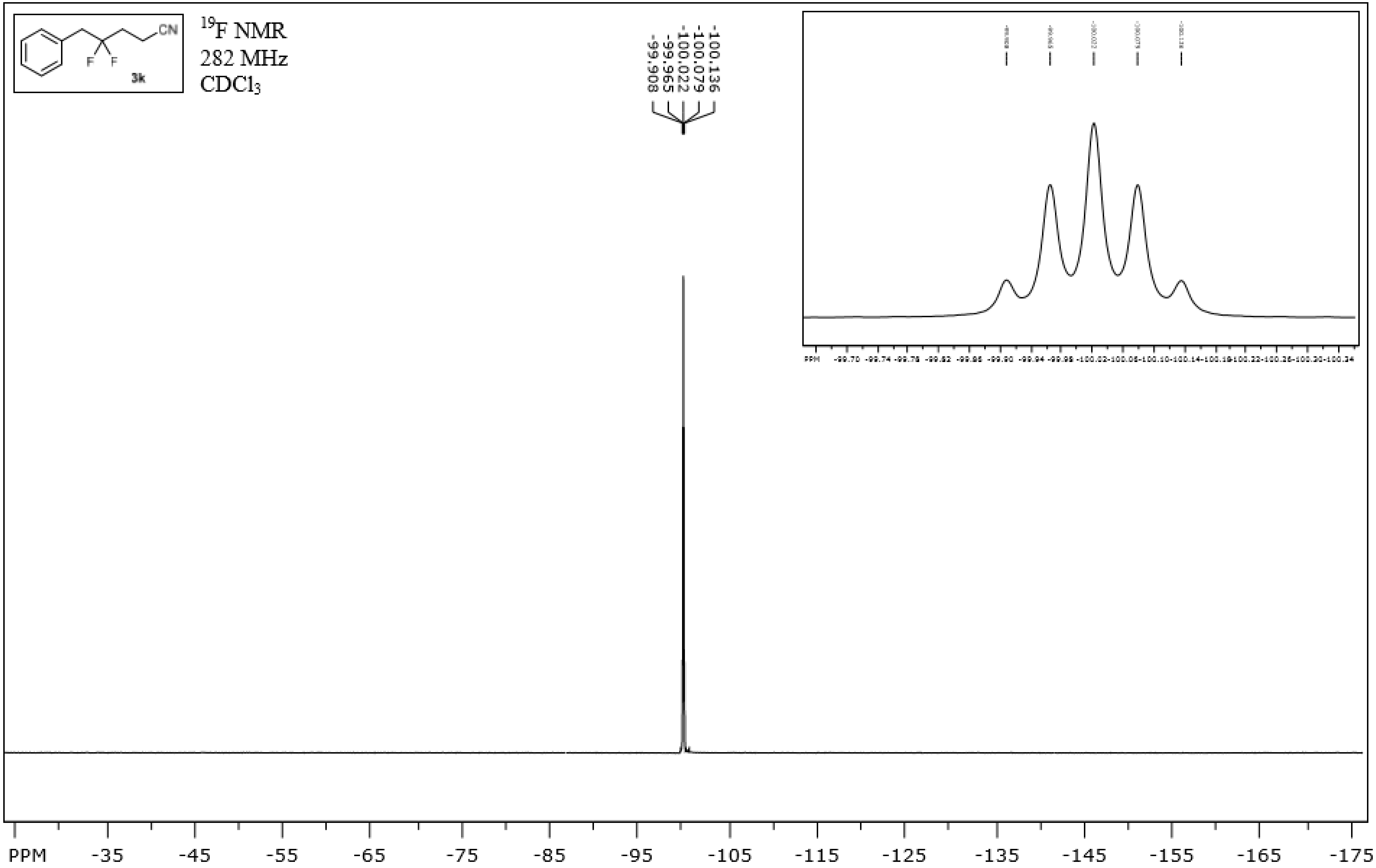
CN
F F
3k
19F NMR
282 MHz
CDCl3
-99.908
-99.965
-100.022
-100.079
-100.136
PPM -35 -45 -55 -65 -75 -85 -95 -105 -115 -125 -135 -145 -155 -165 -175

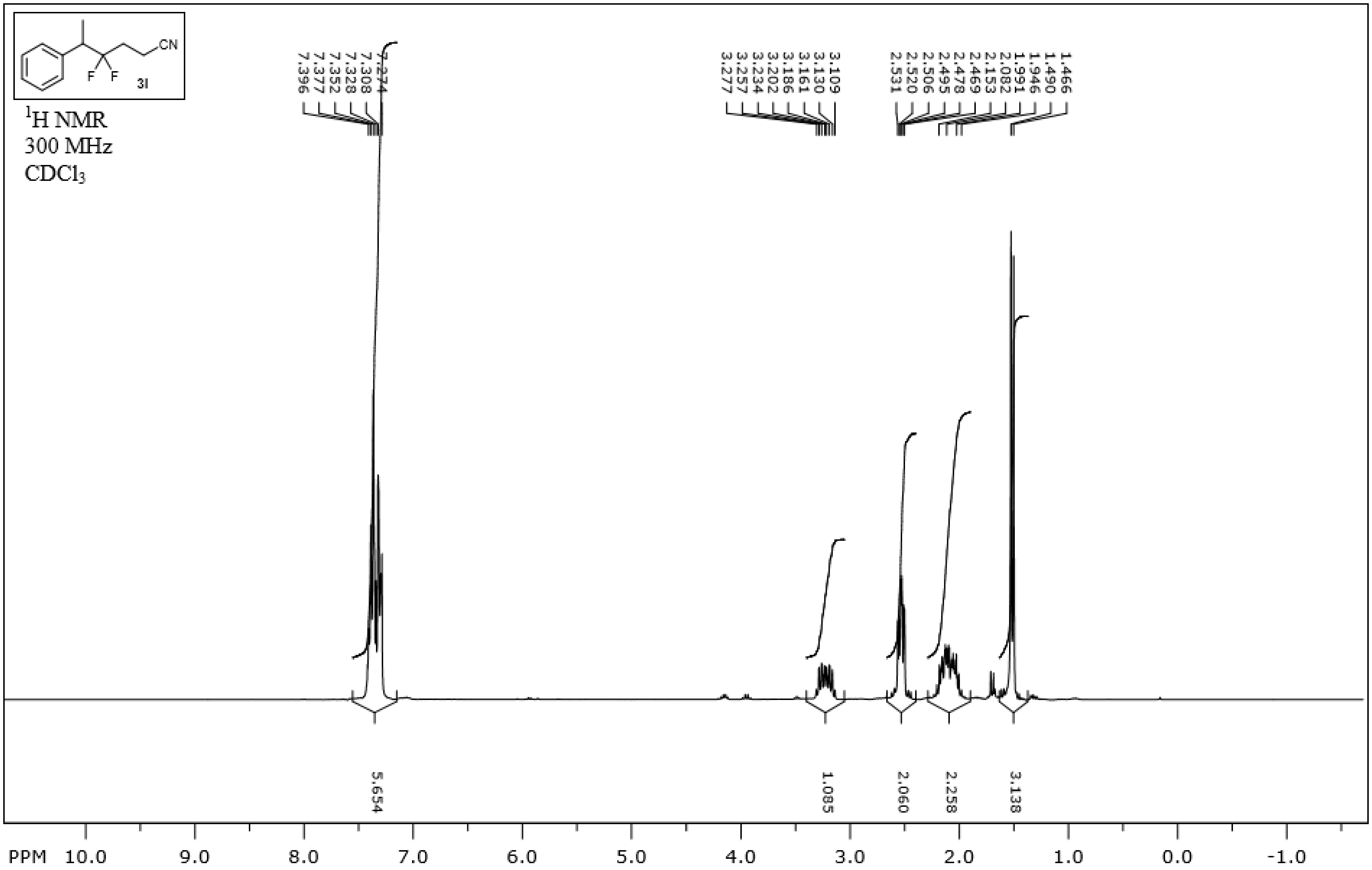
CN
F F
3l
1H NMR
300 MHz
CDCl3
7.396 7.377 7.352 7.328 7.308 7.274
3.277 3.257 3.234 3.202 3.186 3.161 3.130 3.109
2.531 2.520 2.506 2.495 2.478 2.469 2.153 2.082 1.991 1.946 1.490 1.466
5.654
1.085
2.060
2.258
3.138
PPM 10.0 9.0 8.0 7.0 6.0 5.0 4.0 3.0 2.0 1.0 0.0 -1.0

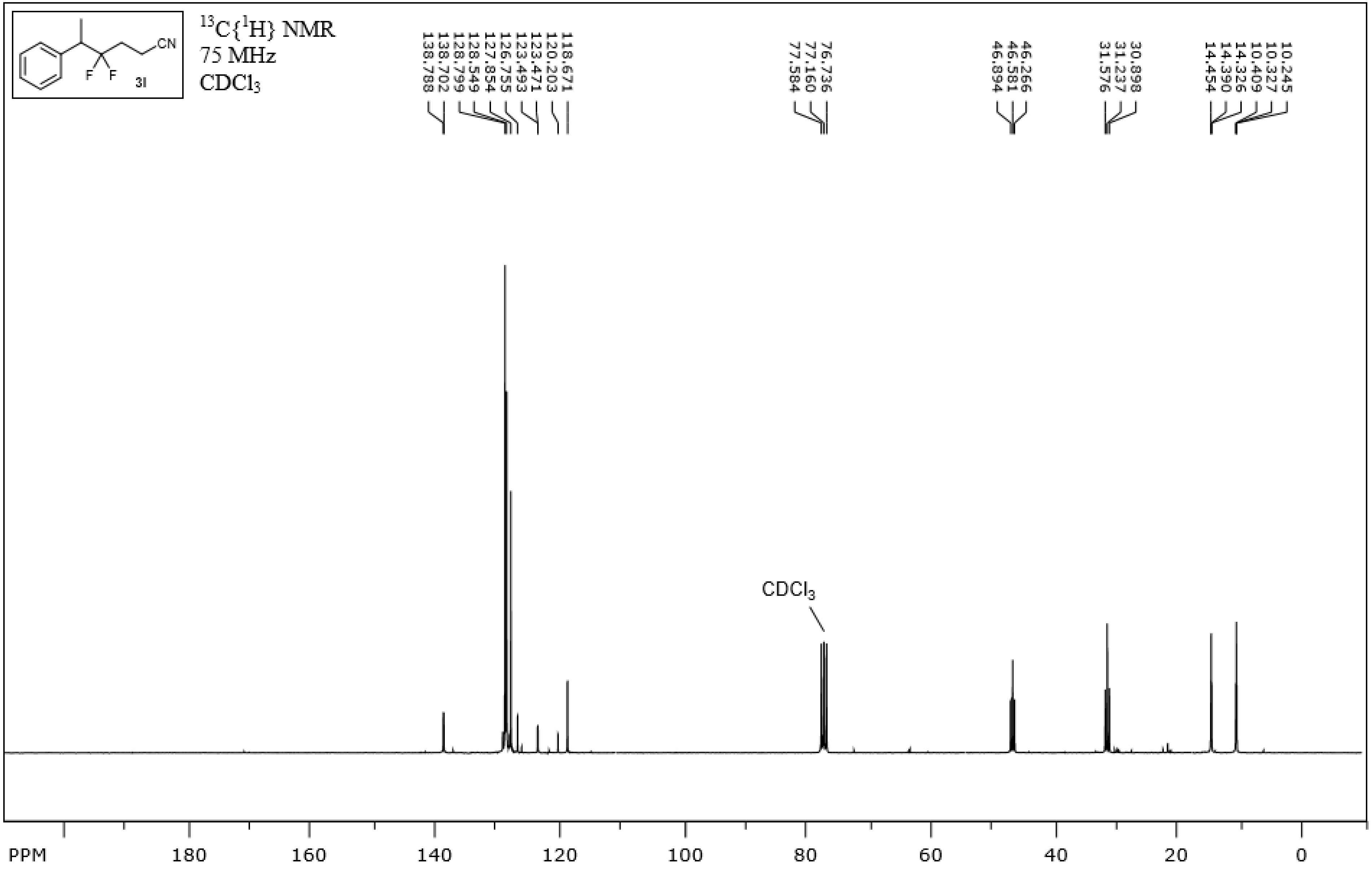
CN
F F
3l
$^{13}C\{^{1}H\}$ NMR
75 MHz
$CDCl_3$
138.788
138.702
128.799
128.549
127.854
126.755
123.493
123.471
120.203
118.671
77.584
77.160
76.736
46.894
46.581
46.266
31.576
31.237
30.898
14.454
14.390
14.326
10.409
10.327
10.245
$CDCl_3$
PPM
180
160
140
120
100
80
60
40
20
0

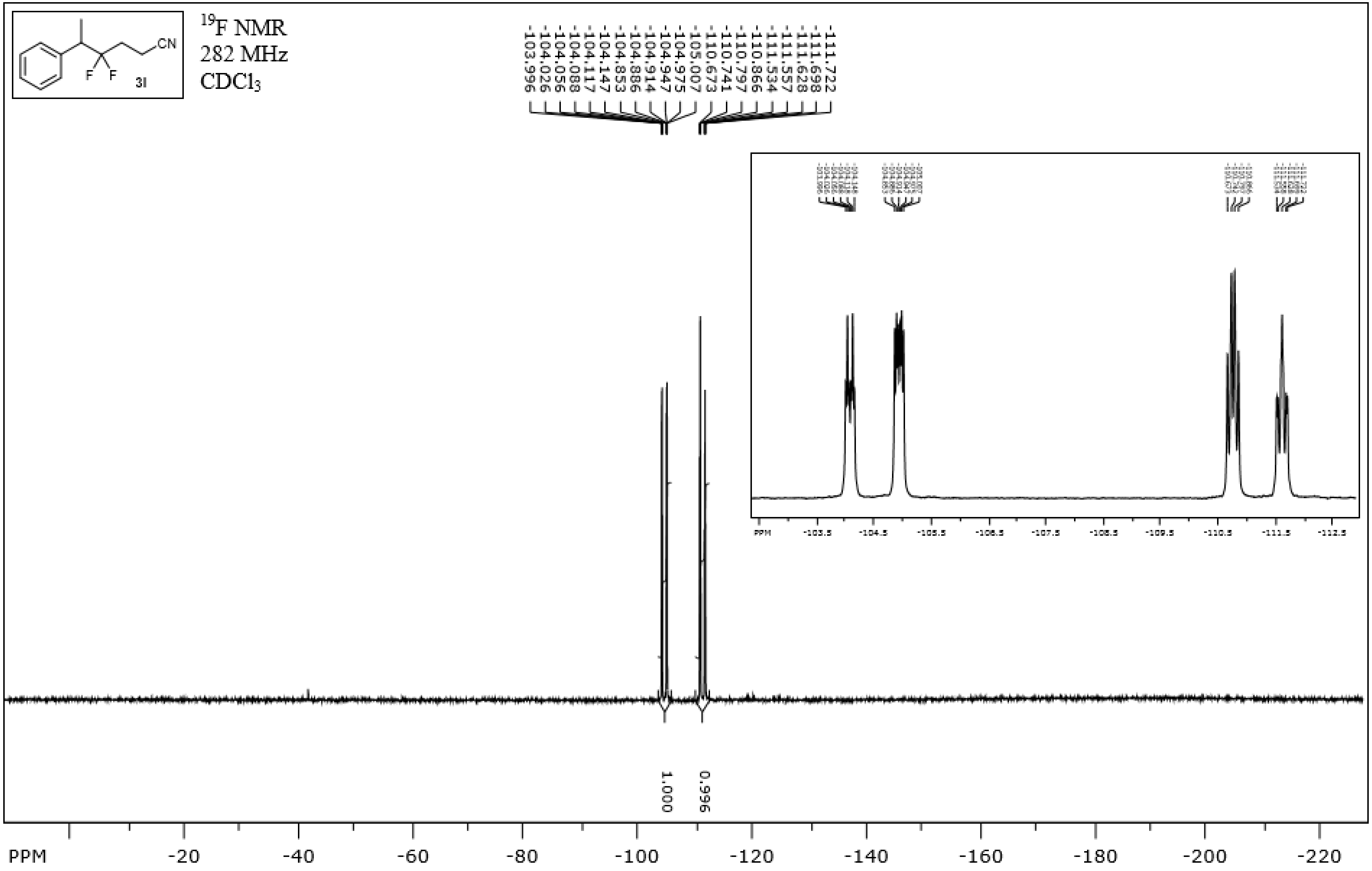
CN
F F
3l
^{19}F NMR
282 MHz
$CDCl_3$
-103.996
-104.026
-104.056
-104.088
-104.117
-104.147
-104.853
-104.886
-104.914
-104.947
-104.975
-105.007
-110.673
-110.741
-110.797
-110.866
-111.534
-111.557
-111.628
-111.698
-111.722
1.000
0.996
PPM
-20
-40
-60
-80
-100
-120
-140
-160
-180
-200
-220

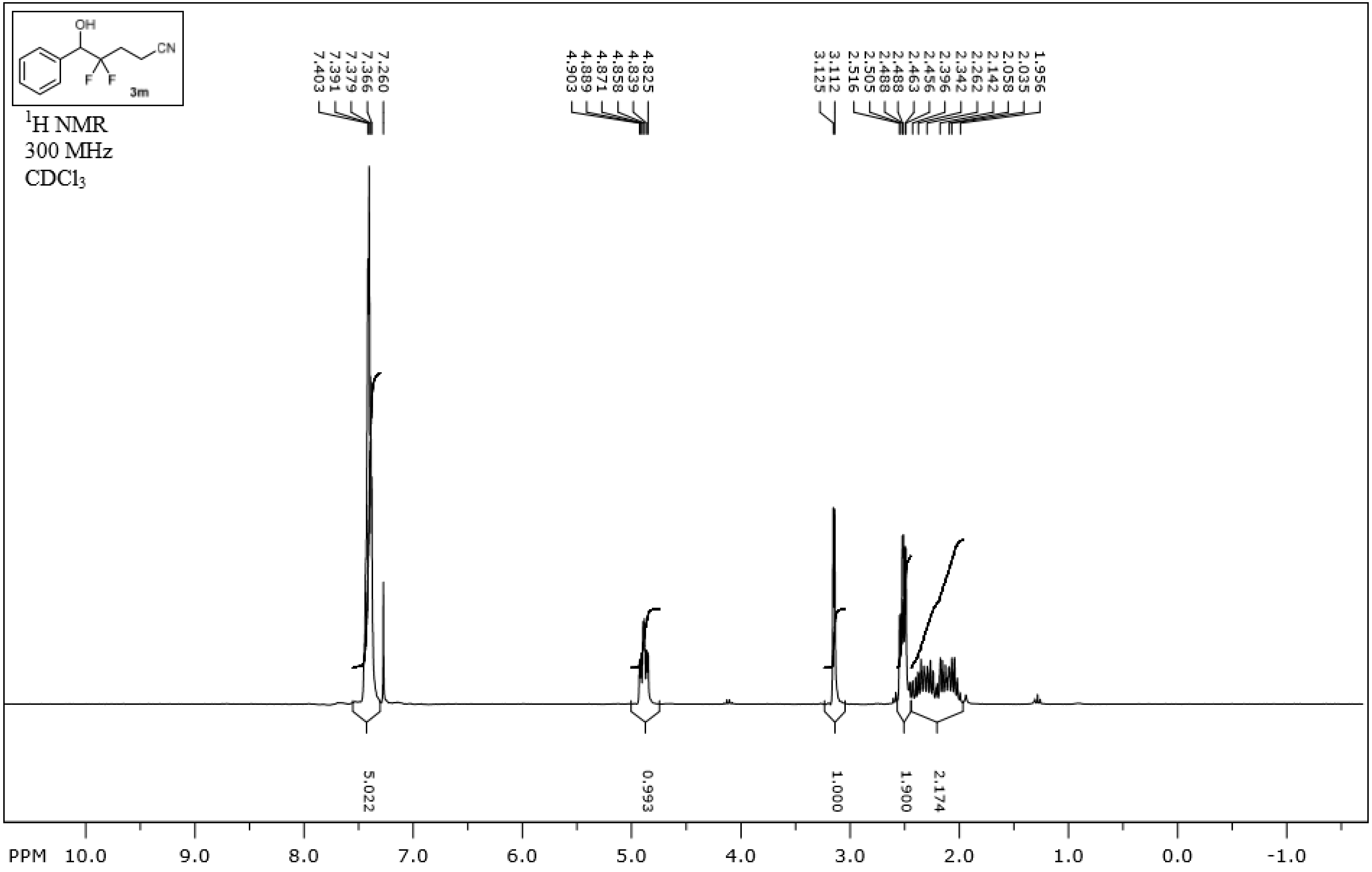

OH
CN
F F
3m
^{1}H NMR
300 MHz
$CDCl_3$
7.403
7.391
7.379
7.366
7.260
4.903
4.889
4.871
4.858
4.839
4.825
3.125
3.112
2.516
2.505
2.488
2.463
2.456
2.396
2.342
2.262
2.142
2.058
2.035
1.956
5.022
0.993
1.000
1.900
2.174
PPM 10.0 9.0 8.0 7.0 6.0 5.0 4.0 3.0 2.0 1.0 0.0 -1.0

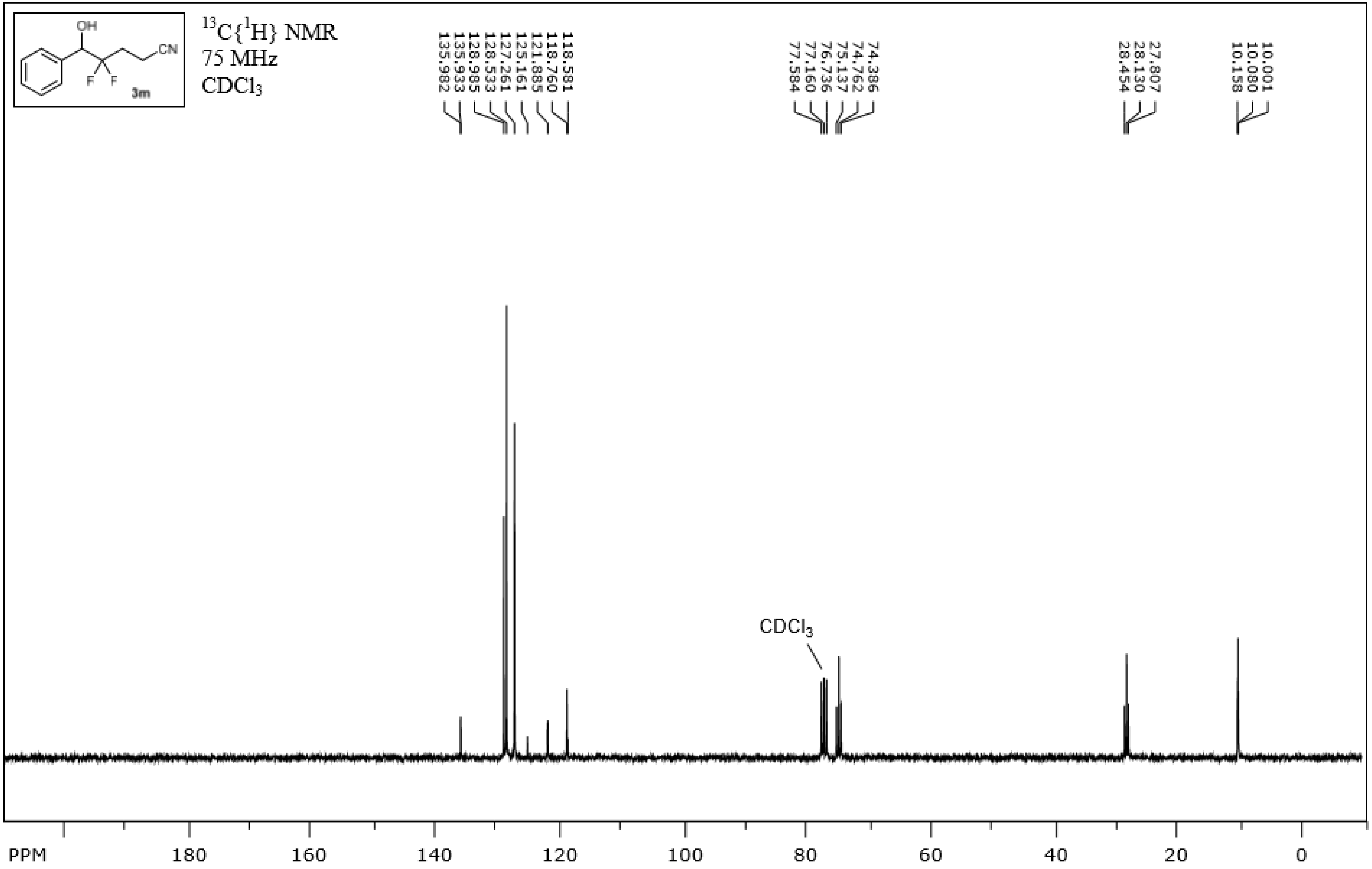
OH
CN
F F
3m
$^{13}C\{^1H\}$ NMR
75 MHz
$CDCl_3$
135.982
135.933
128.985
128.533
127.261
125.161
121.885
118.760
118.581
77.584
77.160
76.736
75.137
74.762
74.386
28.454
28.130
27.807
10.158
10.080
10.001
$CDCl_3$
PPM
180
160
140
120
100
80
60
40
20
0

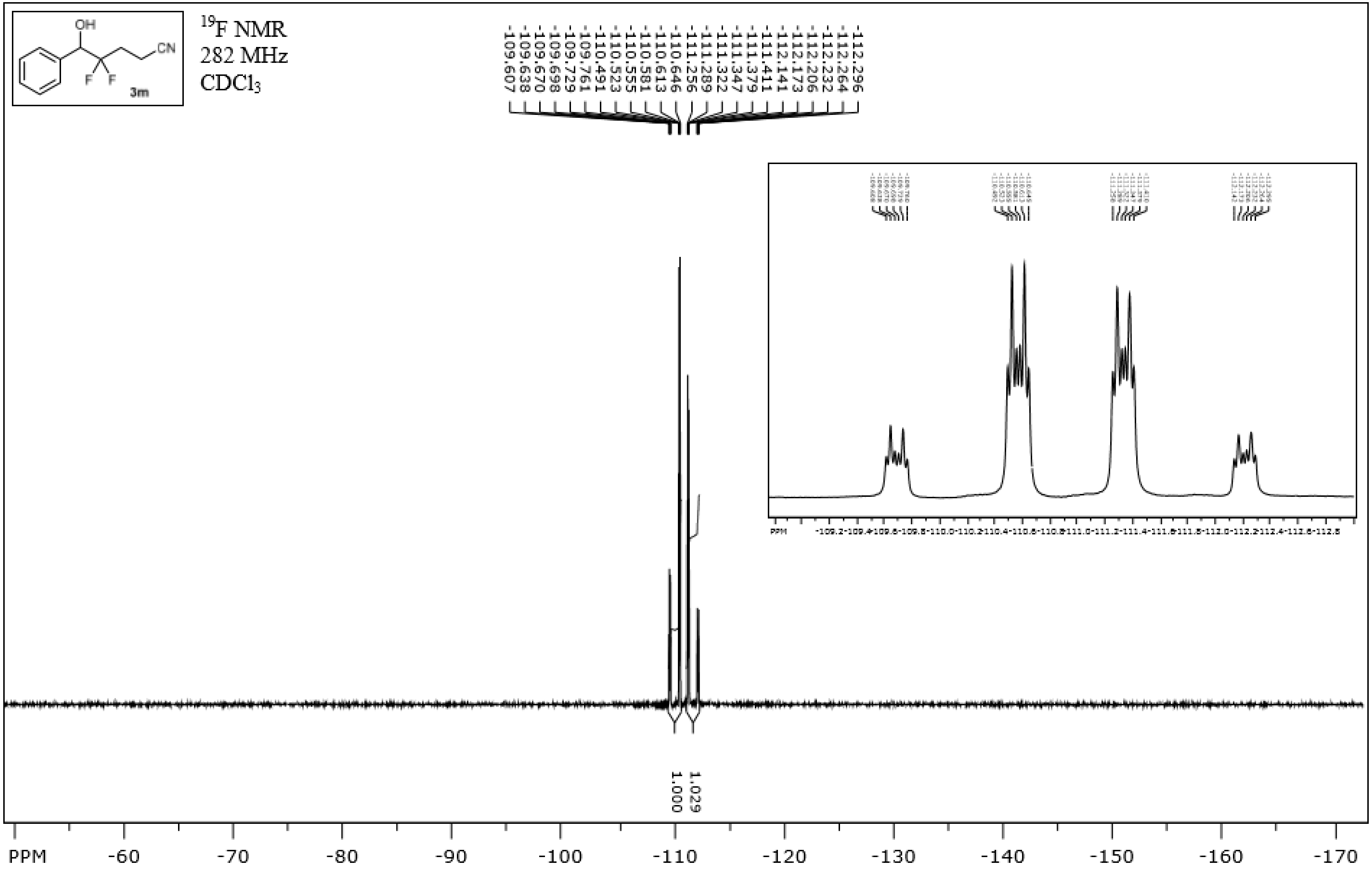
19F NMR
282 MHz
CDCl3
3m
-109.607
-109.638
-109.670
-109.698
-109.729
-109.761
-110.491
-110.523
-110.555
-110.581
-110.613
-110.646
-111.256
-111.289
-111.322
-111.347
-111.379
-111.411
-112.141
-112.173
-112.206
-112.232
-112.264
-112.296
1.000
1.029
PPM
-60
-70
-80
-90
-100
-110
-120
-130
-140
-150
-160
-170

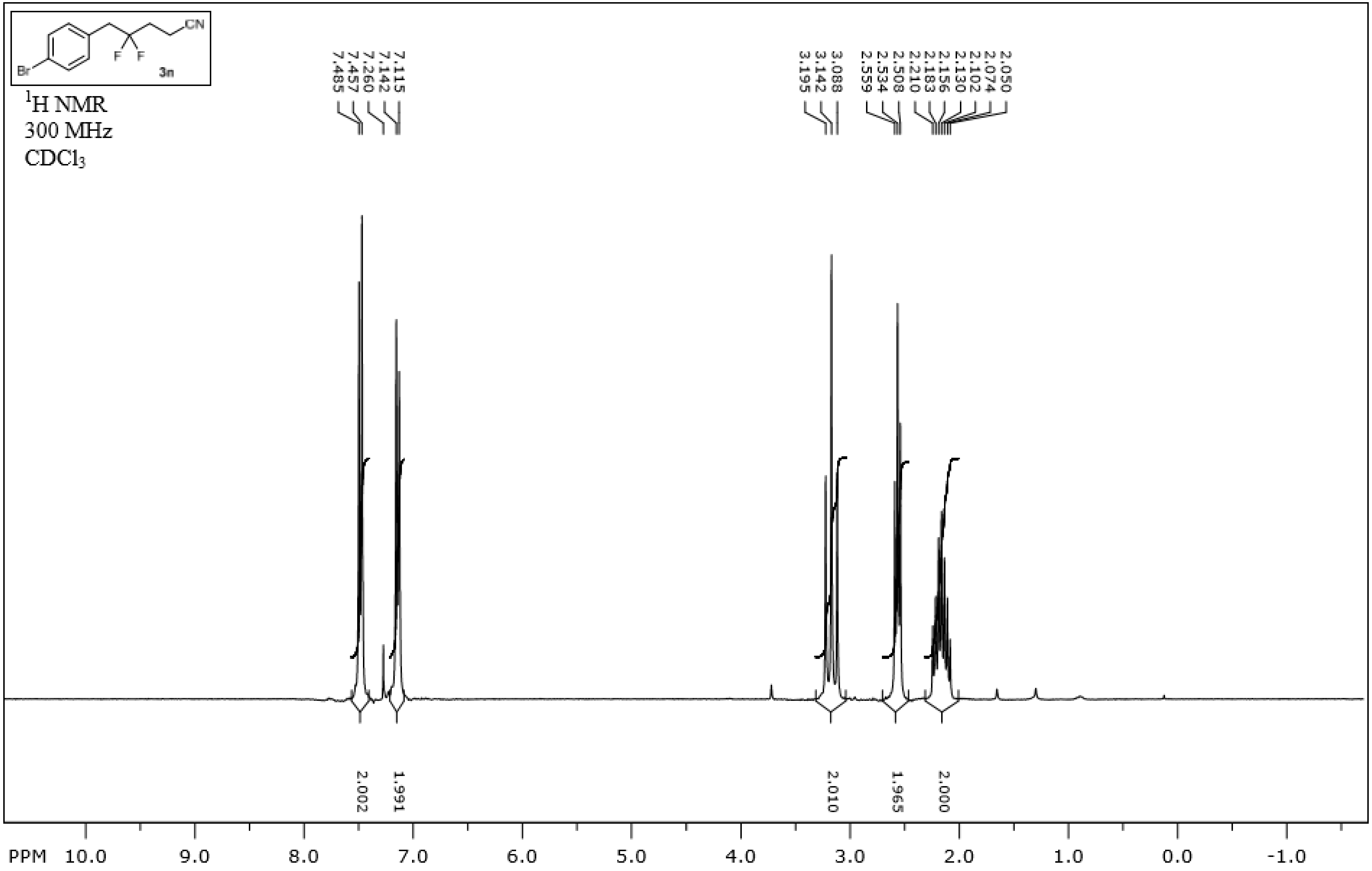

Br
F F
CN
3n
^{1}H NMR
300 MHz
$CDCl_3$
7.485
7.457
7.260
7.142
7.115
3.195
3.142
3.088
2.559
2.534
2.508
2.210
2.183
2.156
2.130
2.102
2.074
2.050
2.002
1.991
2.010
1.965
2.000
PPM 10.0 9.0 8.0 7.0 6.0 5.0 4.0 3.0 2.0 1.0 0.0 -1.0

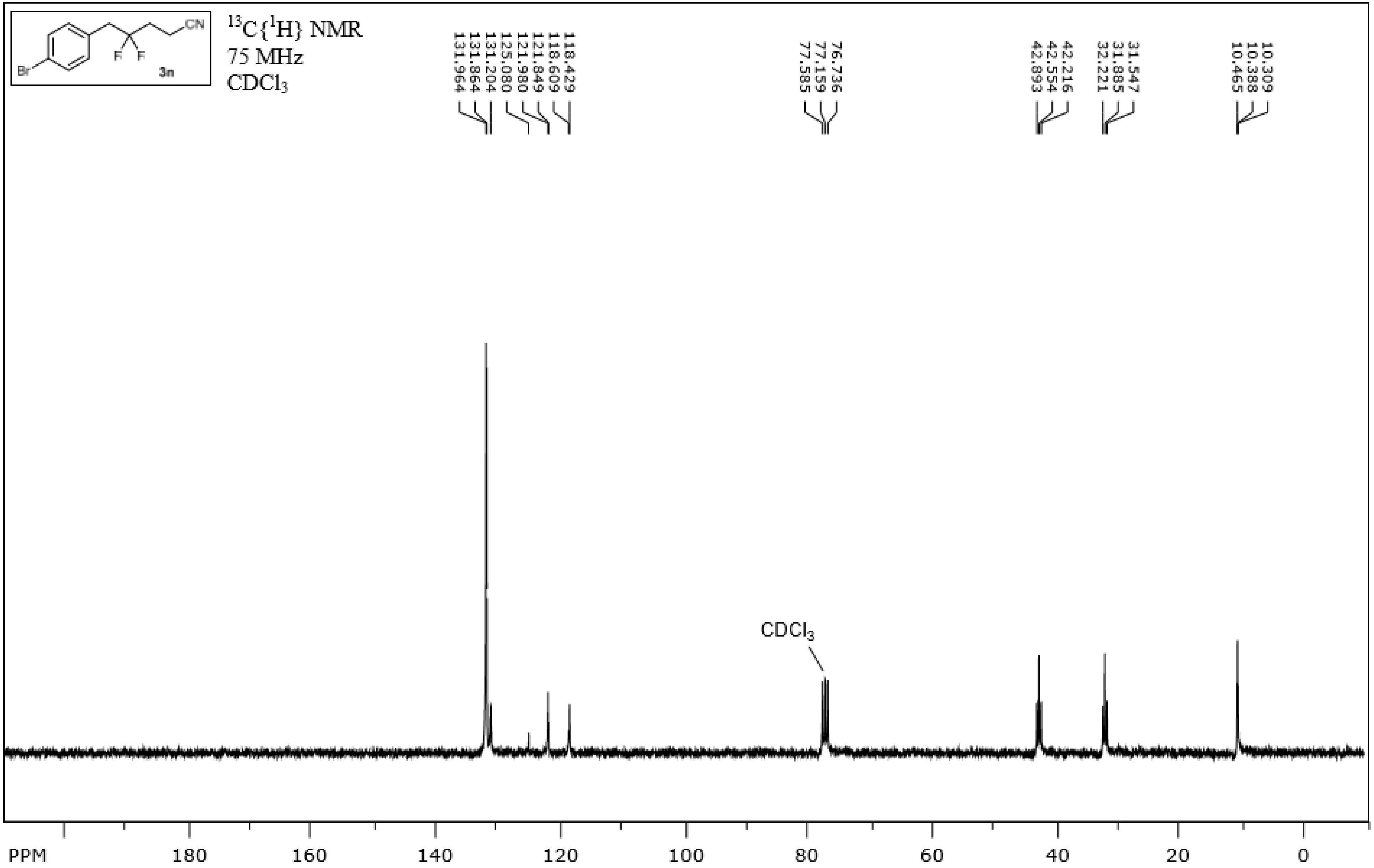

3n
$^{13}C\{^{1}H\}$ NMR
75 MHz
$CDCl_3$
131.964
131.864
131.204
125.080
121.980
121.849
118.609
118.429
77.585
77.159
76.736
42.893
42.554
42.216
32.221
31.885
31.547
10.465
10.388
10.309
CDCl3
PPM
180
160
140
120
100
80
60
40
20
0

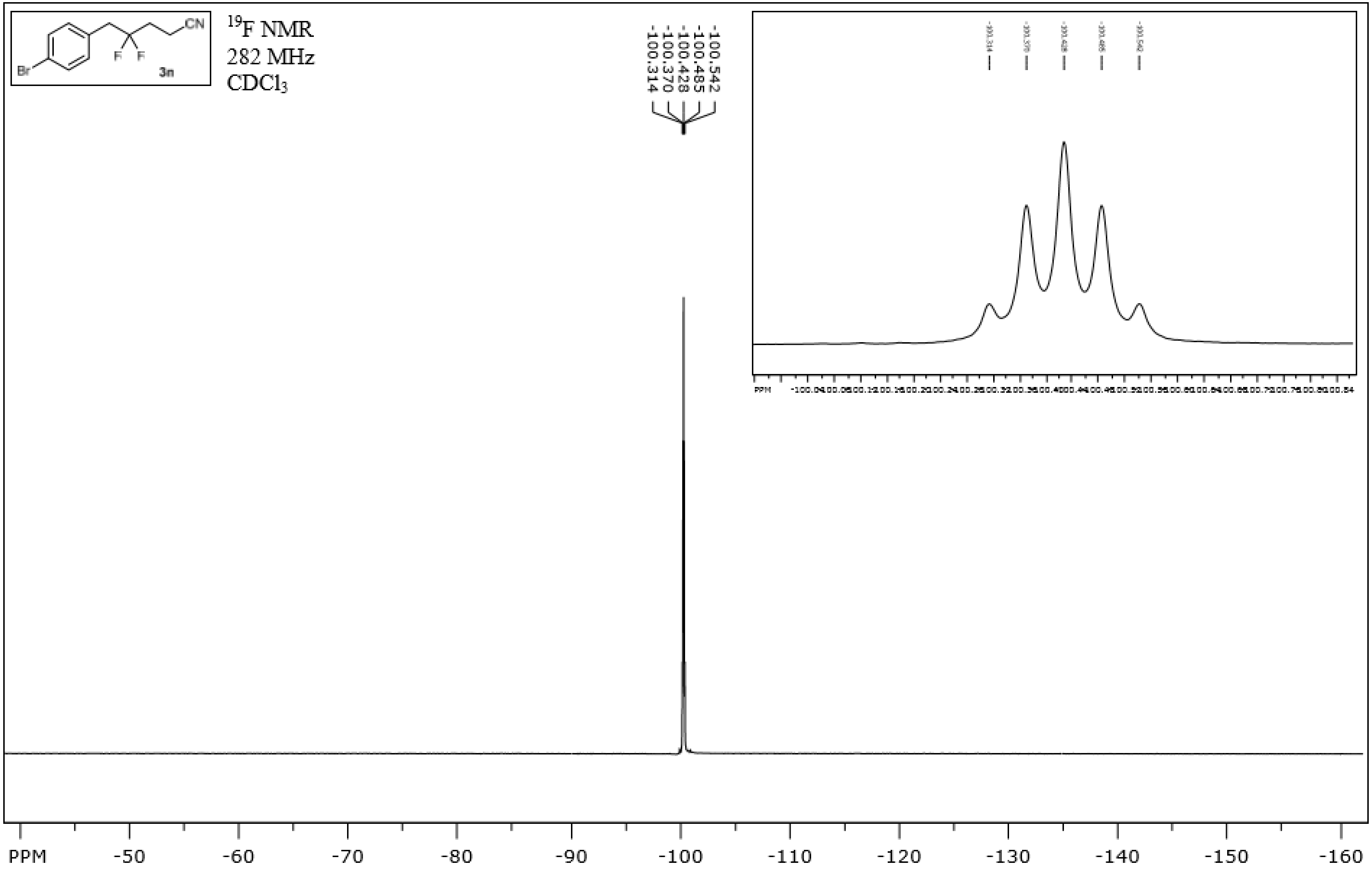

Br
CN
F F
3n
^{19}F NMR
282 MHz
$CDCl_3$
-100.314
-100.370
-100.428
-100.485
-100.542
PPM
-50
-60
-70
-80
-90
-100
-110
-120
-130
-140
-150
-160

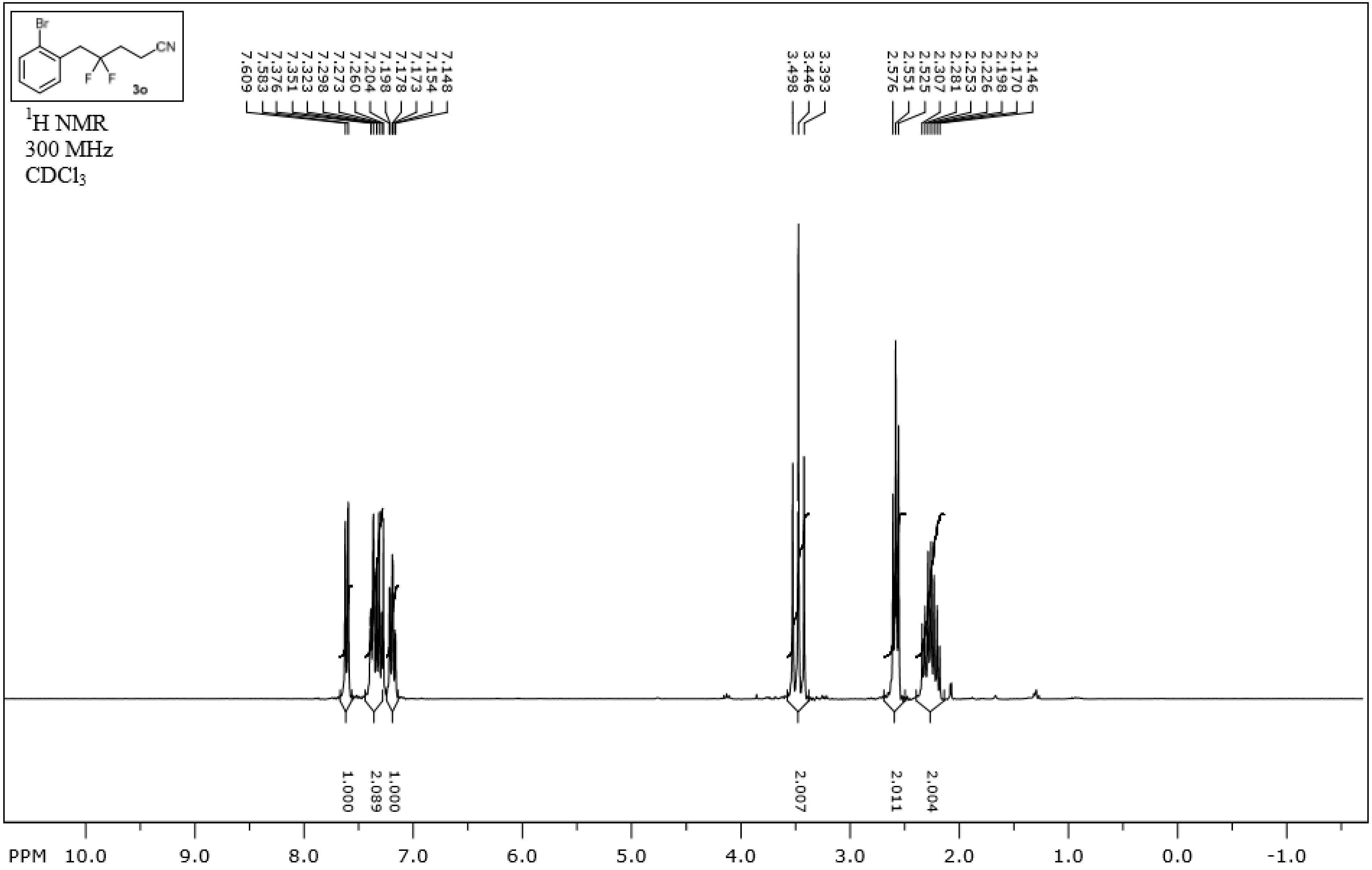

Br
CN
F F
3o
^{1}H NMR
300 MHz
$CDCl_3$
7.609 7.583 7.376 7.351 7.323 7.298 7.273 7.260 7.204 7.198 7.178 7.173 7.154 7.148
3.498 3.446 3.393
2.576 2.551 2.525 2.307 2.281 2.253 2.226 2.198 2.170 2.146
1.000 2.089 1.000 2.007 2.011 2.004
PPM 10.0 9.0 8.0 7.0 6.0 5.0 4.0 3.0 2.0 1.0 0.0 -1.0

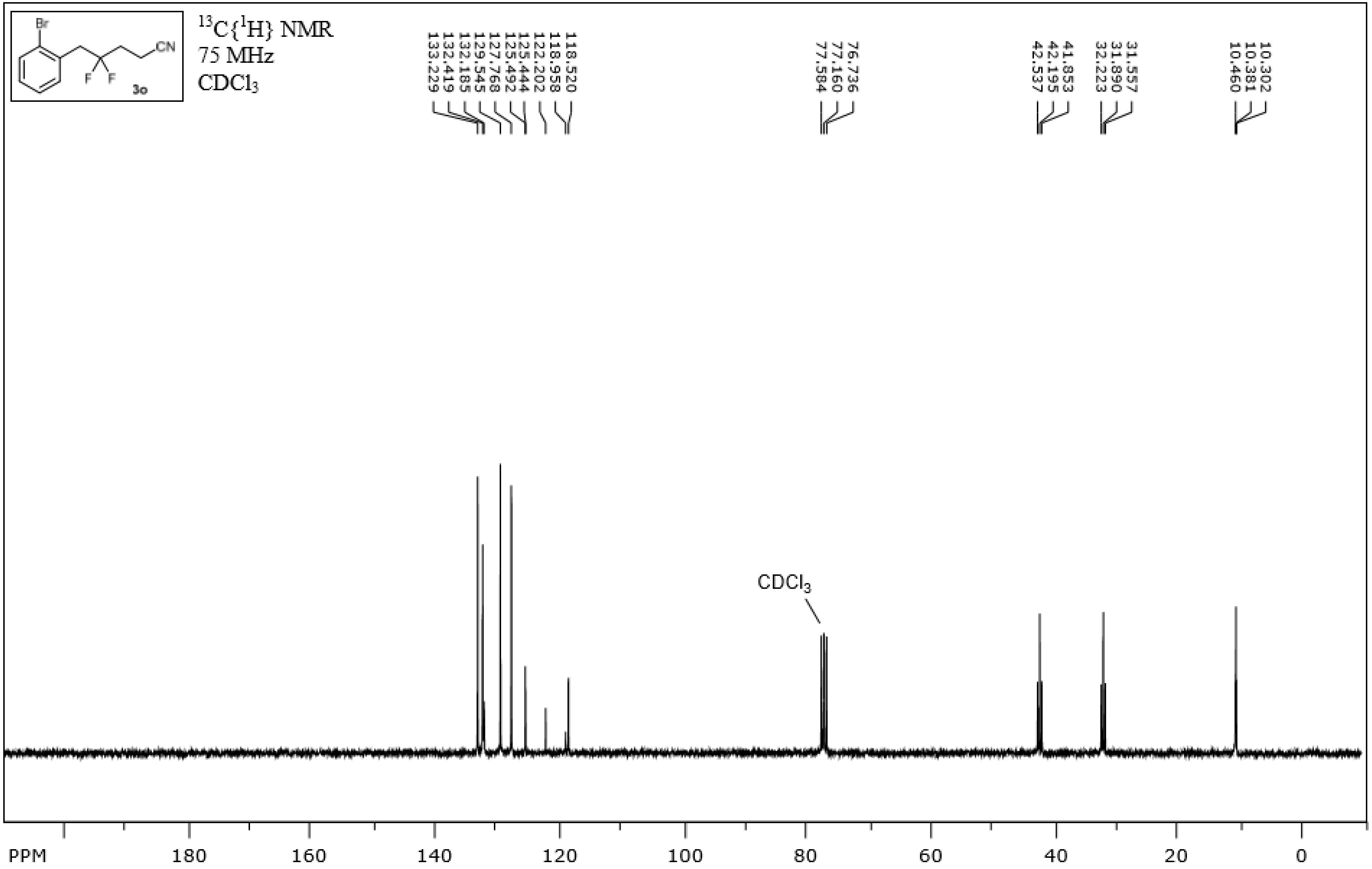

3o
$^{13}C\{^{1}H\}$ NMR
75 MHz
$CDCl_3$
133.229
132.419
132.185
129.545
127.768
125.492
125.444
122.202
118.958
118.520
77.584
77.160
76.736
42.537
42.195
41.853
32.223
31.890
31.557
10.460
10.381
10.302
CDCl3
PPM
180
160
140
120
100
80
60
40
20
0

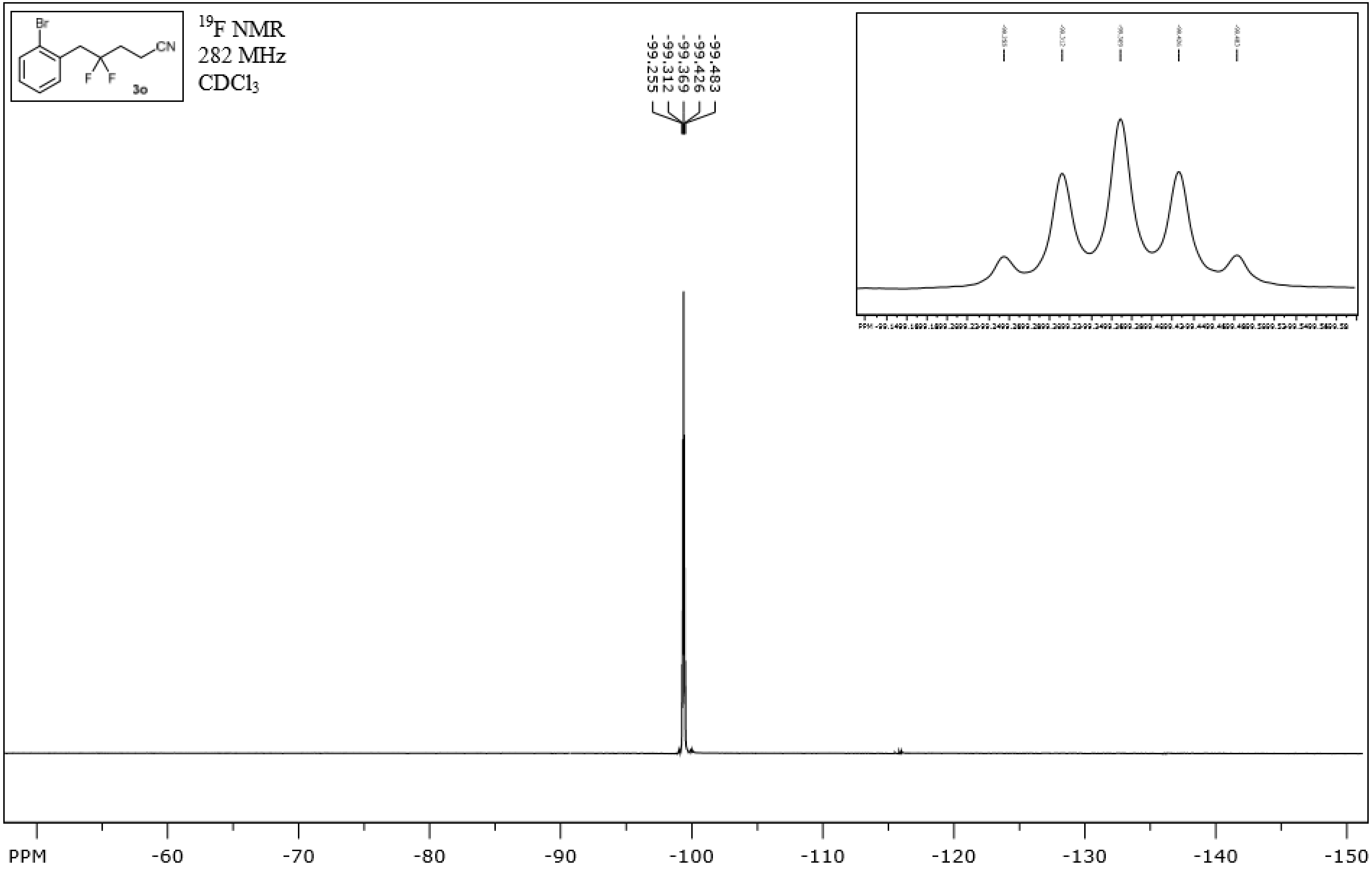

3o
19F NMR
282 MHz
CDCl3
-99.255
-99.312
-99.369
-99.426
-99.483
PPM
-60
-70
-80
-90
-100
-110
-120
-130
-140
-150

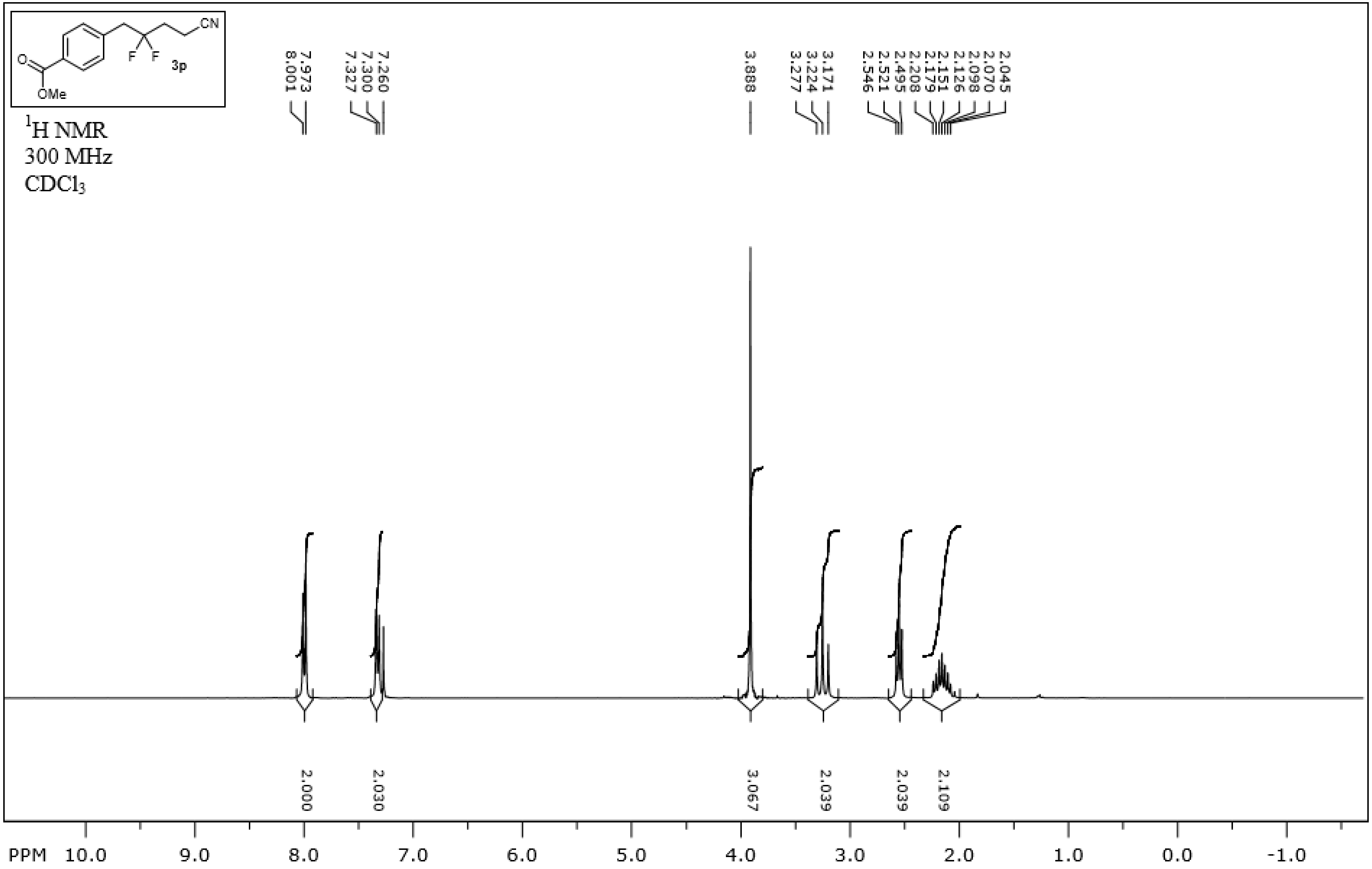
CN
O
F F
3p
OMe
1H NMR
300 MHz
CDCl3
8.001
7.973
7.327
7.300
7.260
3.888
3.277
3.224
3.171
2.546
2.521
2.495
2.208
2.179
2.151
2.126
2.098
2.070
2.045
2.000
2.030
3.067
2.039
2.039
2.109
PPM 10.0 9.0 8.0 7.0 6.0 5.0 4.0 3.0 2.0 1.0 0.0 -1.0

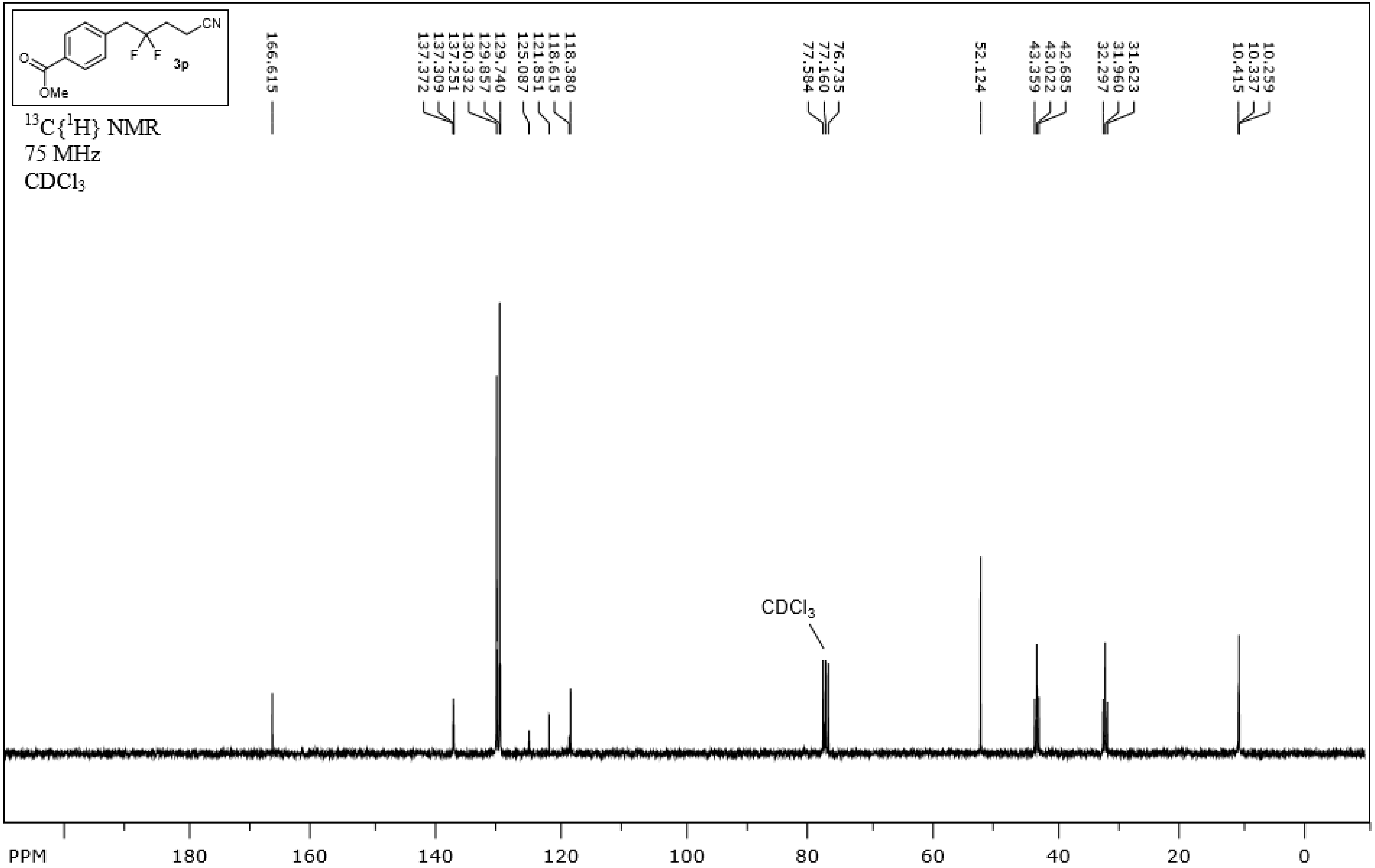
CN
F F
3p
O
OMe
13C{1H} NMR
75 MHz
CDCl3
166.615
137.372
137.309
137.251
130.332
129.857
129.740
125.087
121.851
118.615
118.380
77.584
77.160
76.735
52.124
43.359
43.022
42.685
32.297
31.960
31.623
10.415
10.337
10.259
CDCl3
PPM
180
160
140
120
100
80
60
40
20
0

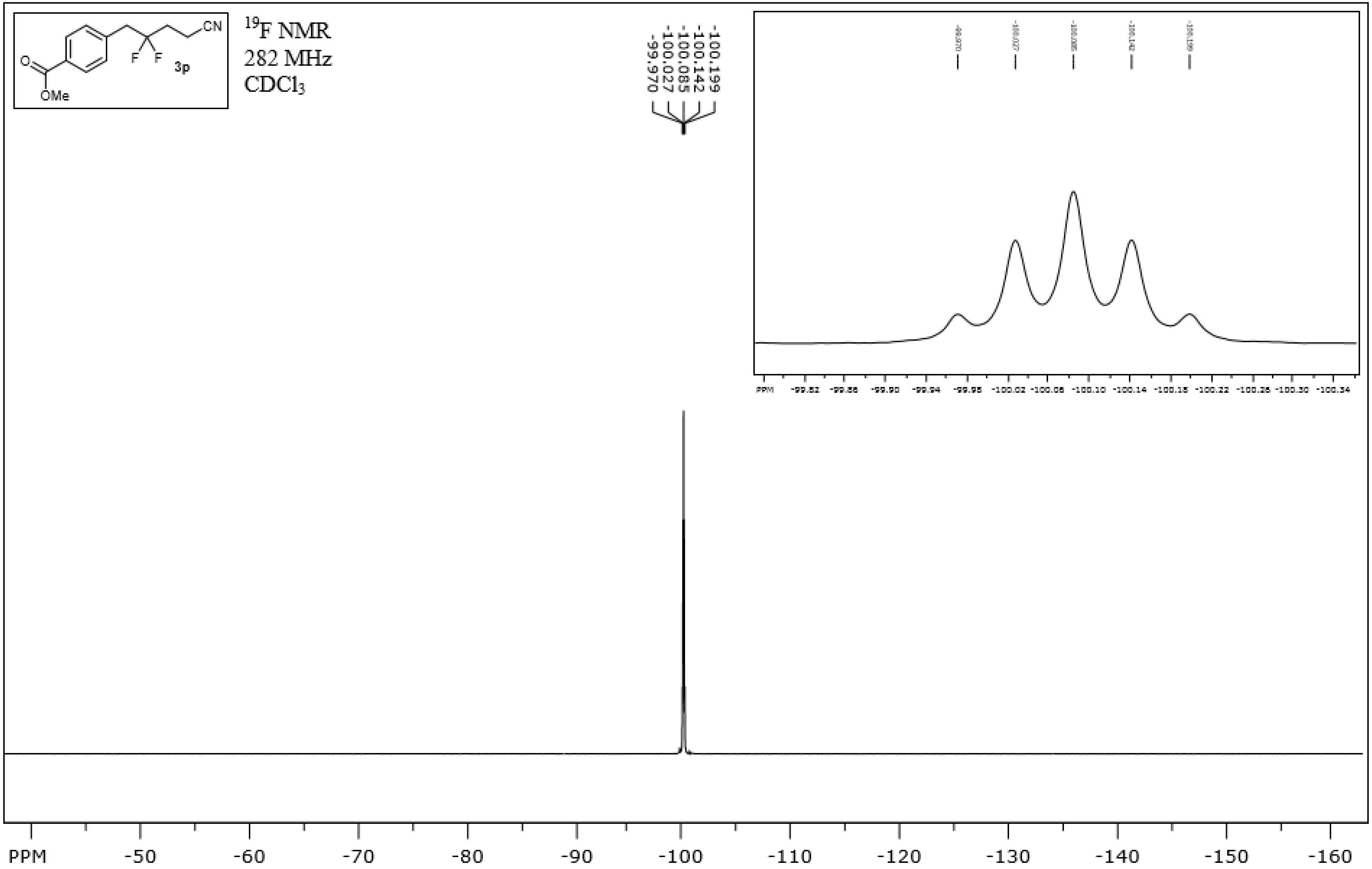
OMe
CN
F F
3p
^{19}F NMR
282 MHz
$CDCl_3$
-99.970
-100.027
-100.085
-100.142
-100.199
PPM
-50
-60
-70
-80
-90
-100
-110
-120
-130
-140
-150
-160

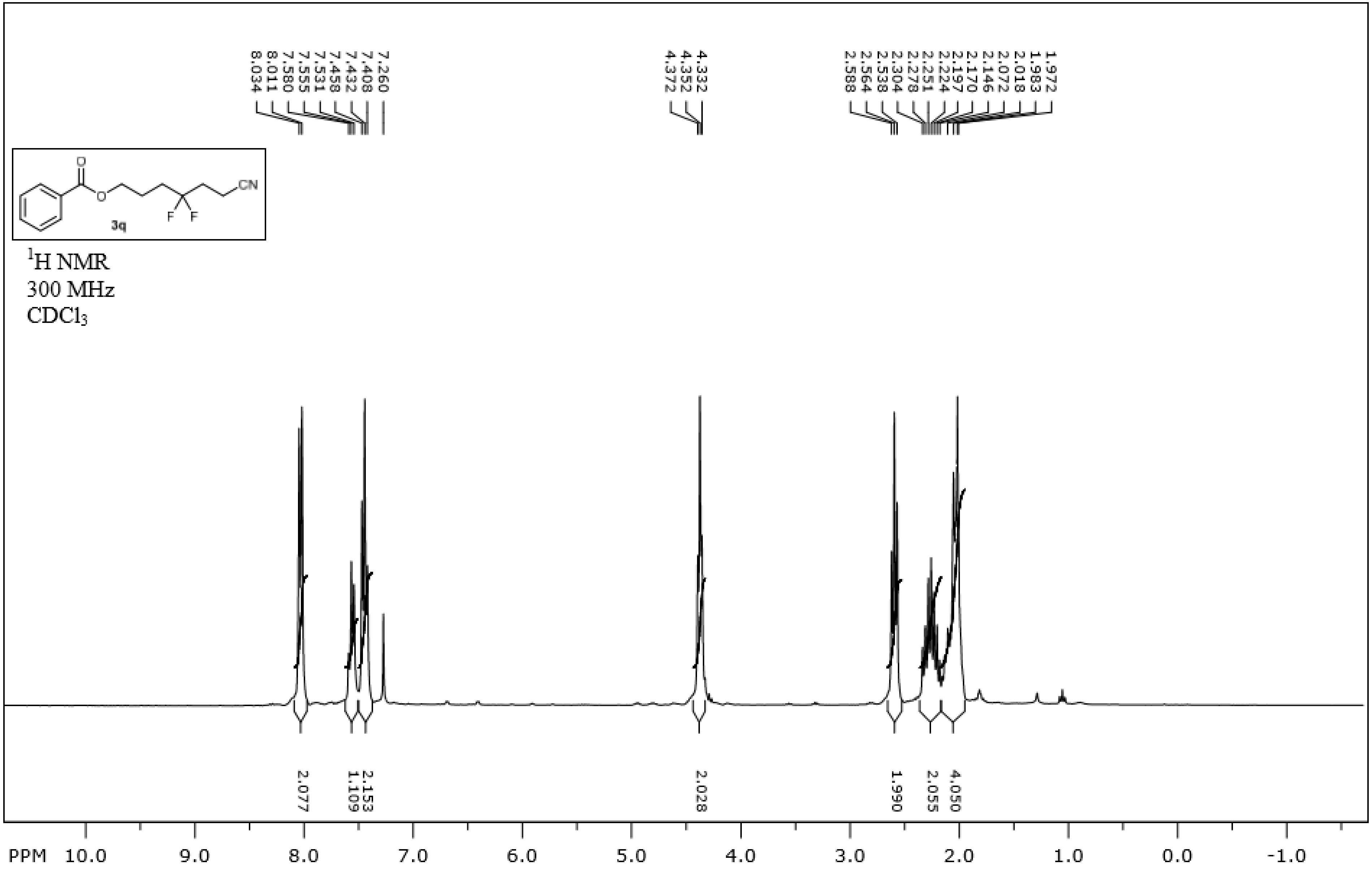

3q
CN
O
F F
¹H NMR
300 MHz
CDCl3
8.034
8.011
7.580
7.555
7.531
7.458
7.432
7.408
7.260
4.372
4.352
4.332
2.588
2.564
2.538
2.304
2.278
2.251
2.224
2.197
2.170
2.146
2.072
2.018
1.983
1.972
2.077
1.109
2.153
2.028
1.990
2.055
4.050
PPM 10.0 9.0 8.0 7.0 6.0 5.0 4.0 3.0 2.0 1.0 0.0 -1.0

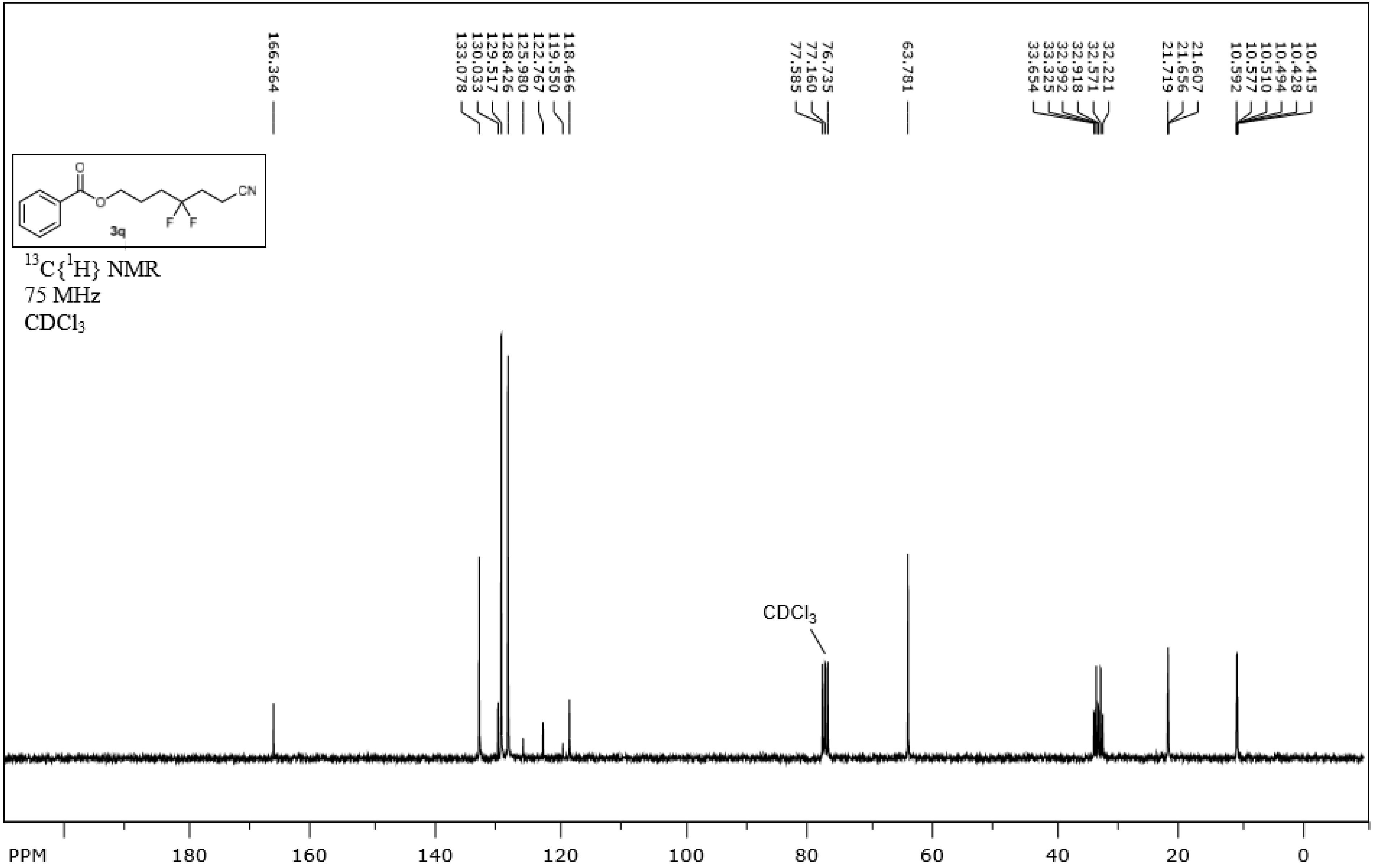

3q
CN
F F
O
$^{13}C\{^{1}H\}$ NMR
75 MHz
$CDCl_3$
$CDCl_3$
166.364
133.078
130.033
129.517
128.426
125.980
122.767
119.550
118.466
77.585
77.160
76.735
63.781
33.654
33.325
32.992
32.918
32.571
32.221
21.719
21.656
21.607
10.592
10.577
10.510
10.494
10.428
10.415
PPM
180
160
140
120
100
80
60
40
20
0

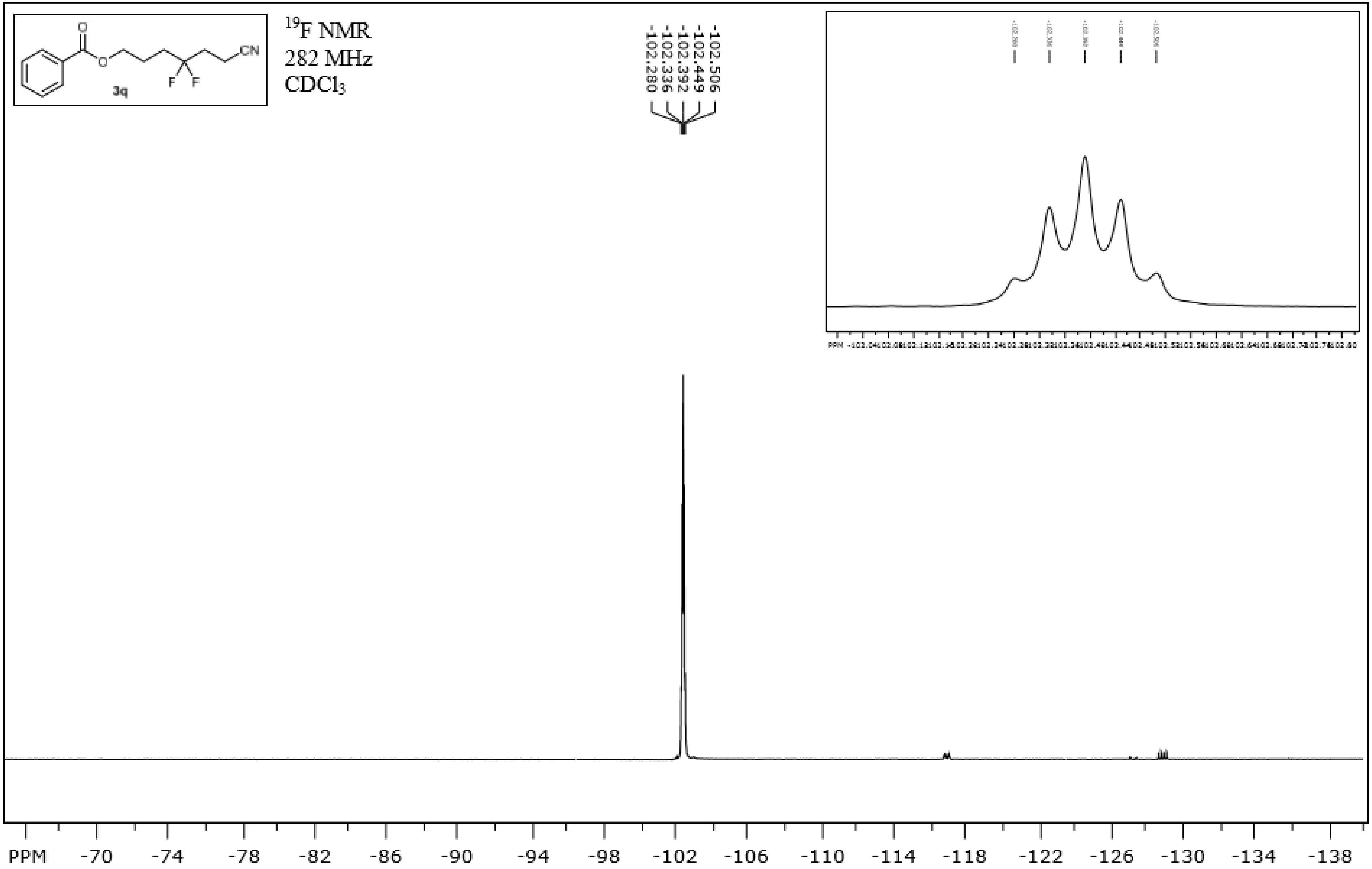
3q
^{19}F NMR
282 MHz
$CDCl_3$
-102.506
-102.449
-102.392
-102.336
-102.280
PPM -70 -74 -78 -82 -86 -90 -94 -98 -102 -106 -110 -114 -118 -122 -126 -130 -134 -138

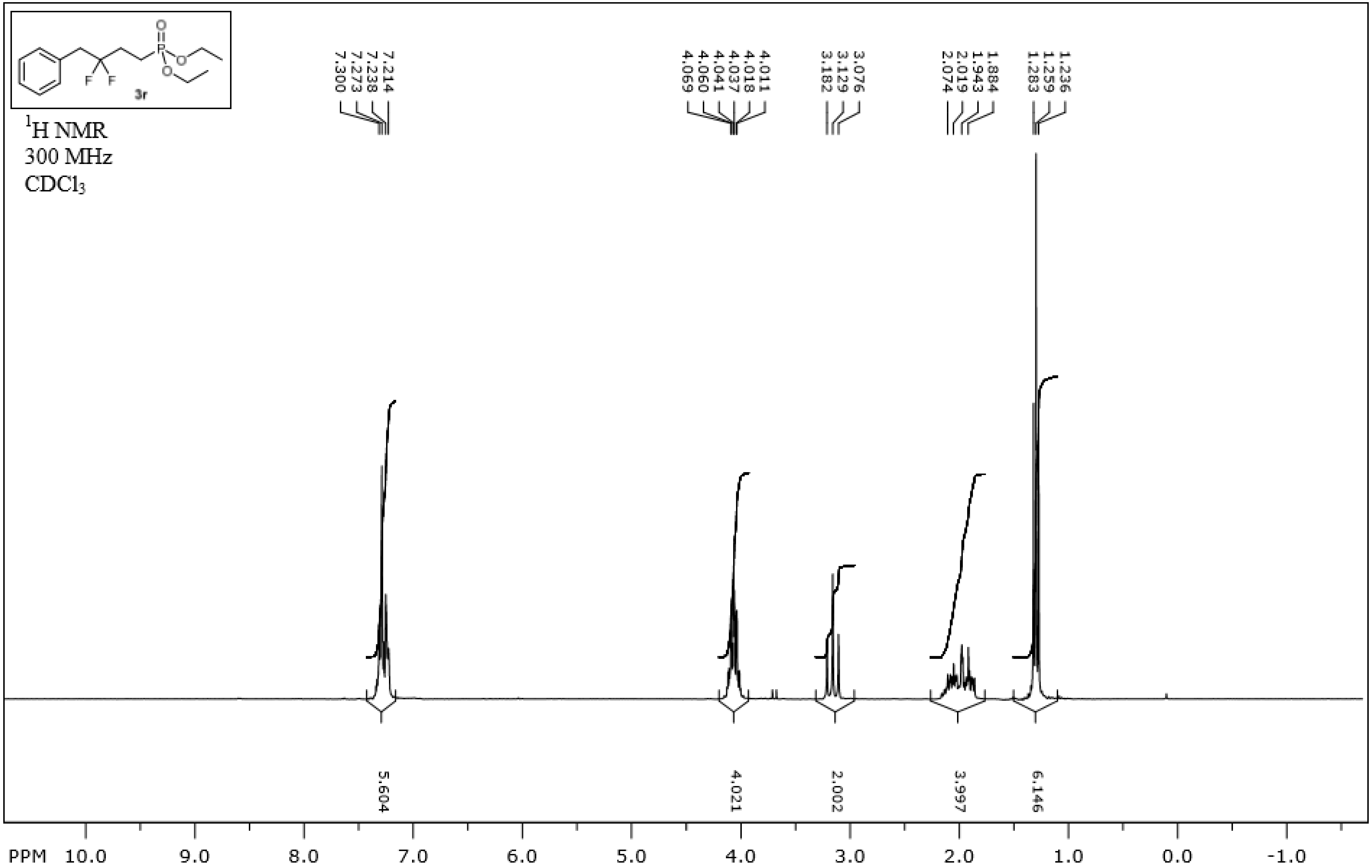
3r
1H NMR
300 MHz
CDCl3
7.300
7.273
7.238
7.214
4.069
4.060
4.041
4.037
4.018
4.011
3.182
3.129
3.076
2.074
2.019
1.943
1.884
1.283
1.259
1.236
5.604
4.021
2.002
3.997
6.146
PPM 10.0 9.0 8.0 7.0 6.0 5.0 4.0 3.0 2.0 1.0 0.0 -1.0

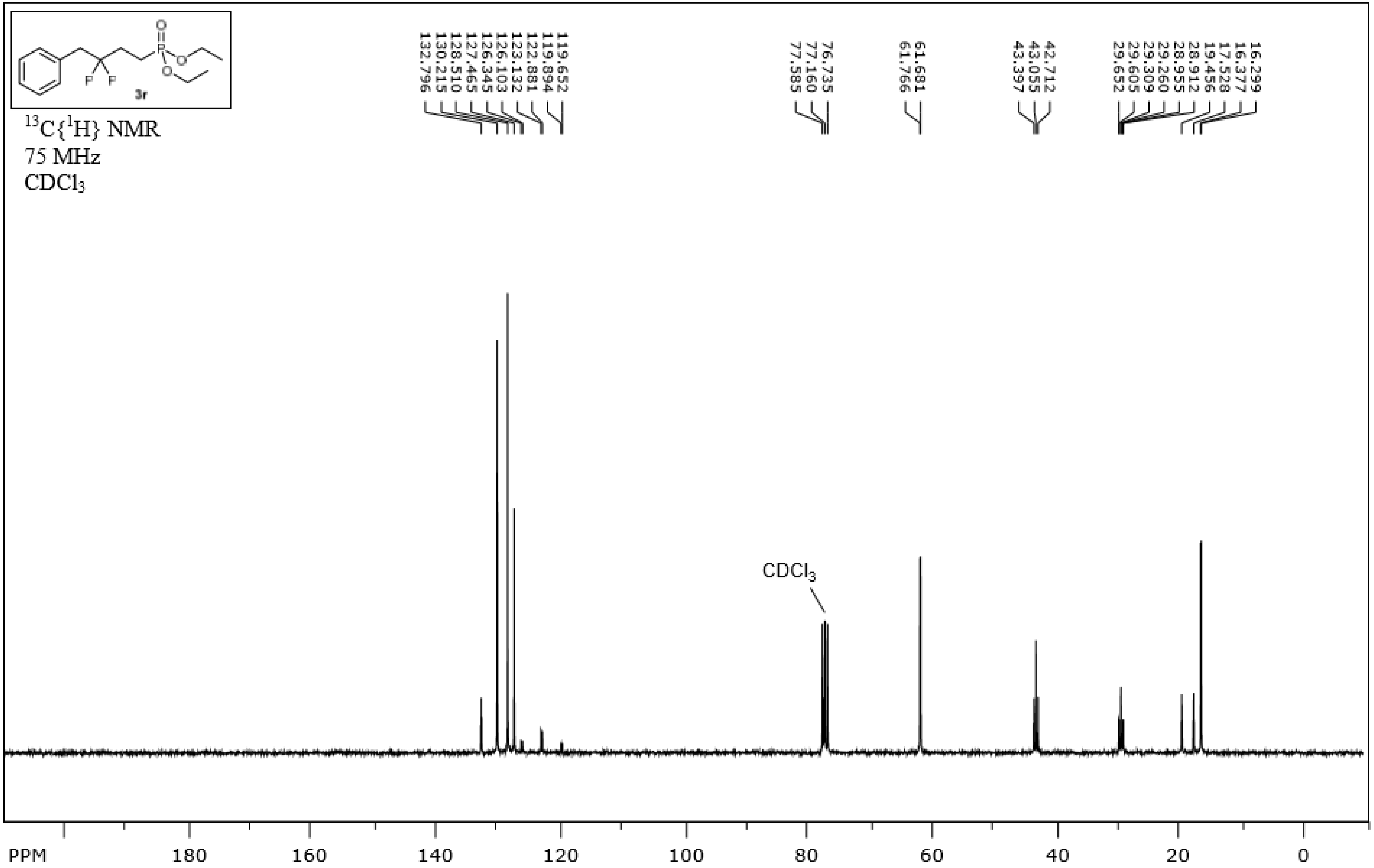
3r
$^{13}C\{^1H\}$ NMR
75 MHz
$CDCl_3$
132.796
130.215
128.510
127.465
126.345
126.103
123.132
122.881
119.894
119.652
77.585
77.160
76.735
61.766
61.681
43.397
43.055
42.712
29.652
29.605
29.309
29.260
28.955
28.912
19.456
17.528
16.377
16.299
CDCl3
PPM
180
160
140
120
100
80
60
40
20
0

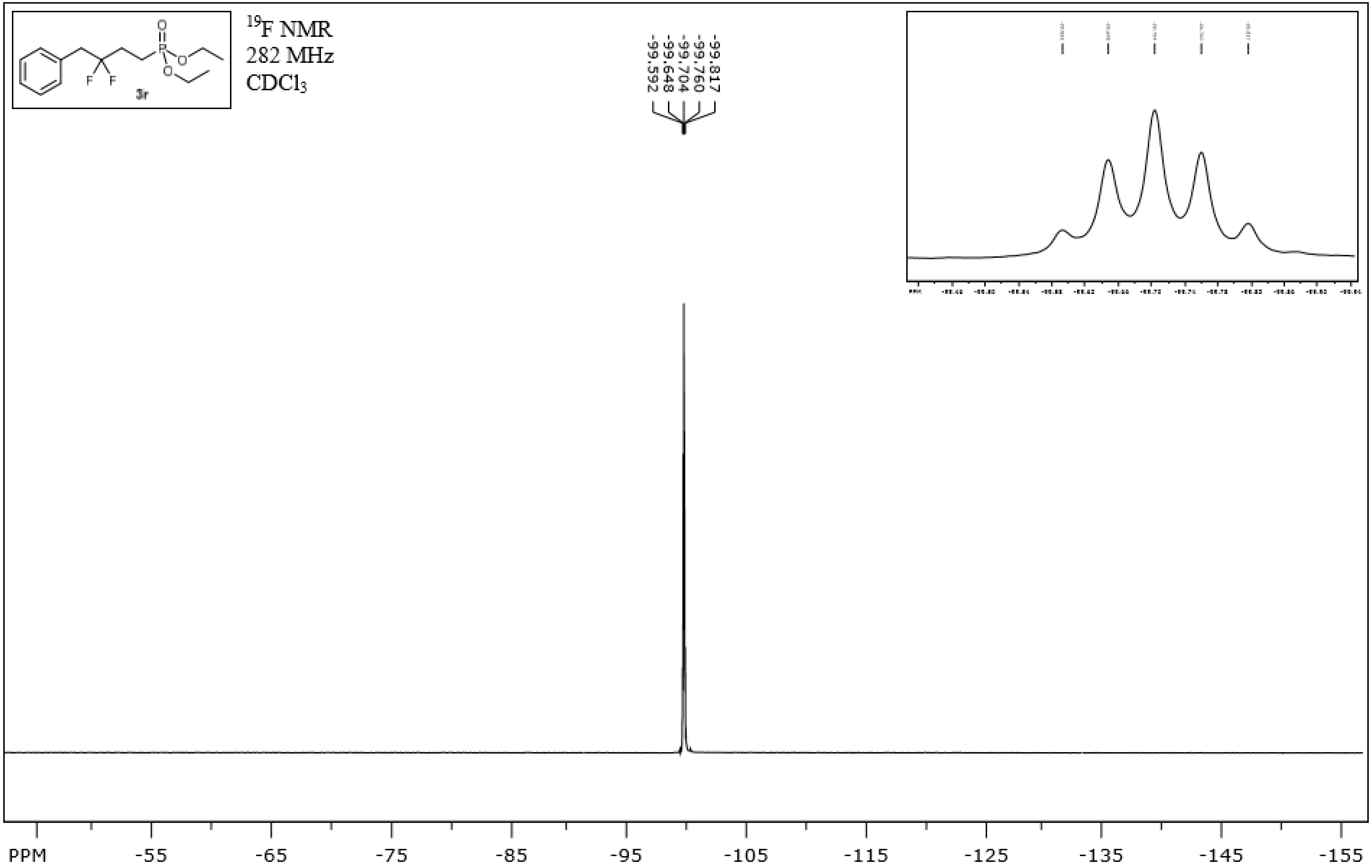

3r
^{19}F NMR
282 MHz
$CDCl_3$
-99.592
-99.648
-99.704
-99.760
-99.817
PPM
-55
-65
-75
-85
-95
-105
-115
-125
-135
-145
-155

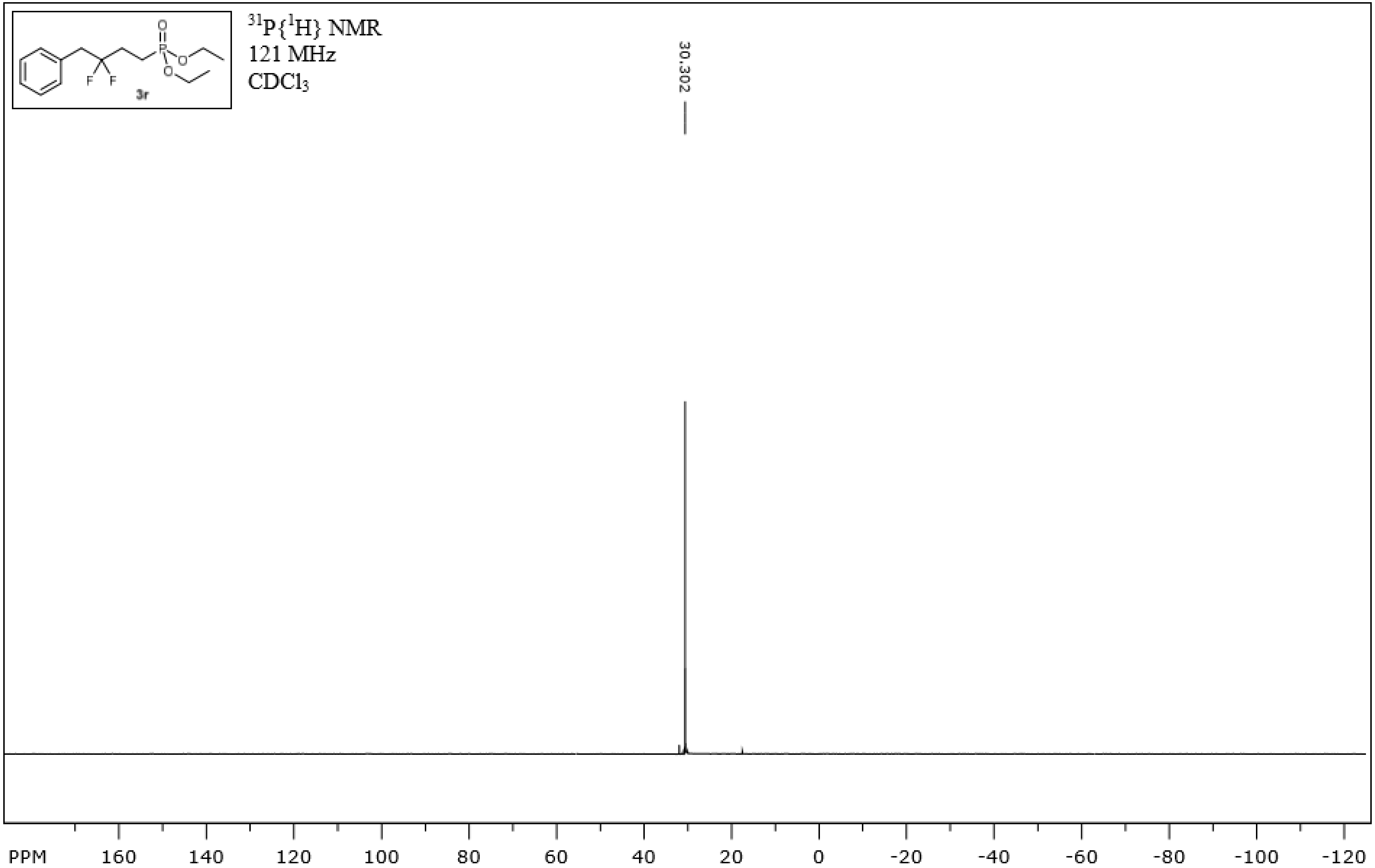
3r
$^{31}P\{^{1}H\}$ NMR
121 MHz
$CDCl_3$
30.302
PPM
160
140
120
100
80
60
40
20
0
-20
-40
-60
-80
-100
-120

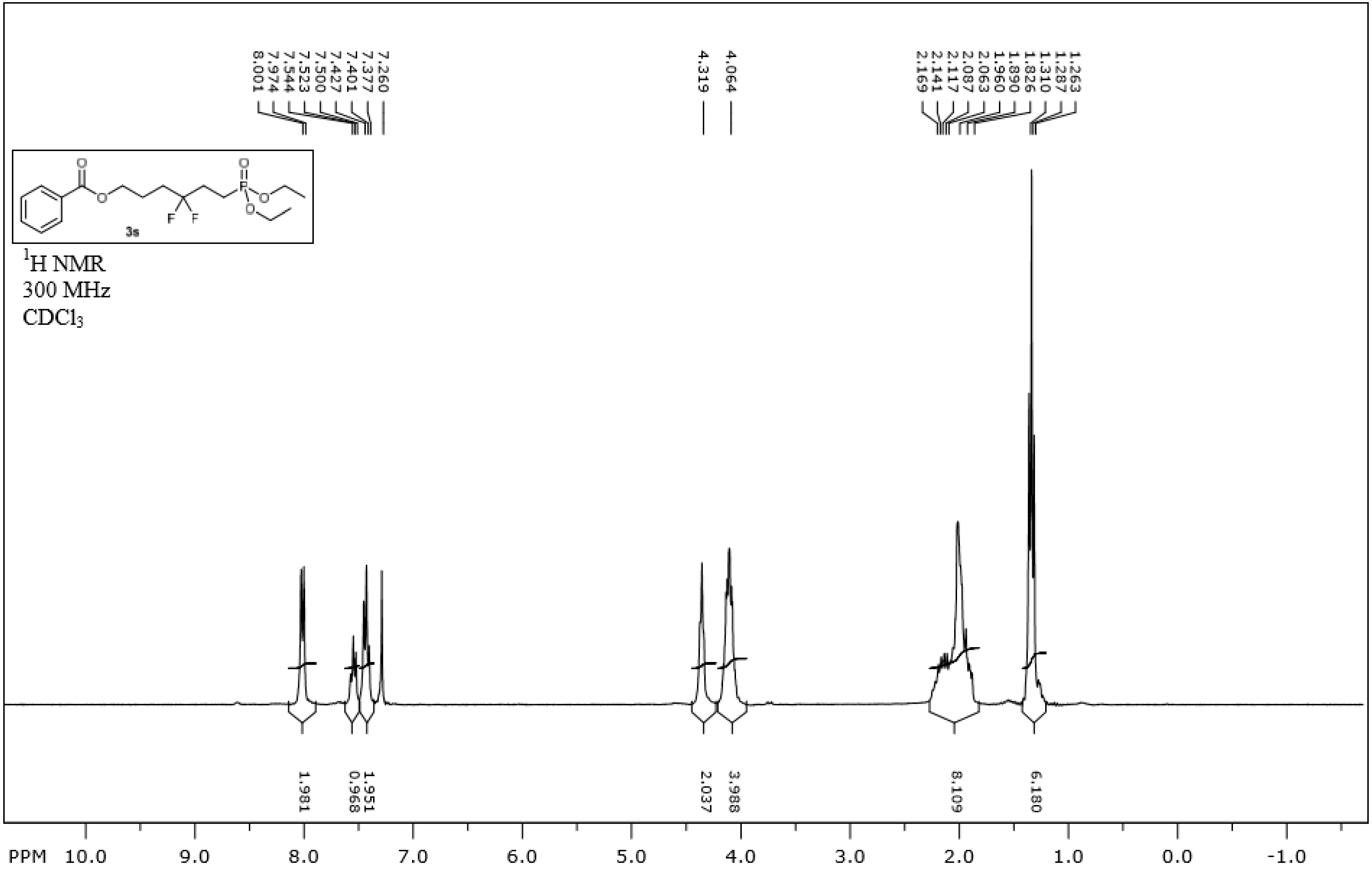
3s
^{1}H NMR
300 MHz
$CDCl_3$
8.001
7.974
7.544
7.523
7.500
7.427
7.401
7.377
7.260
4.319
4.064
2.169
2.141
2.117
2.087
2.063
1.960
1.890
1.826
1.310
1.287
1.263
1.981
0.968
1.951
2.037
3.988
8.109
6.180
PPM 10.0 9.0 8.0 7.0 6.0 5.0 4.0 3.0 2.0 1.0 0.0 -1.0

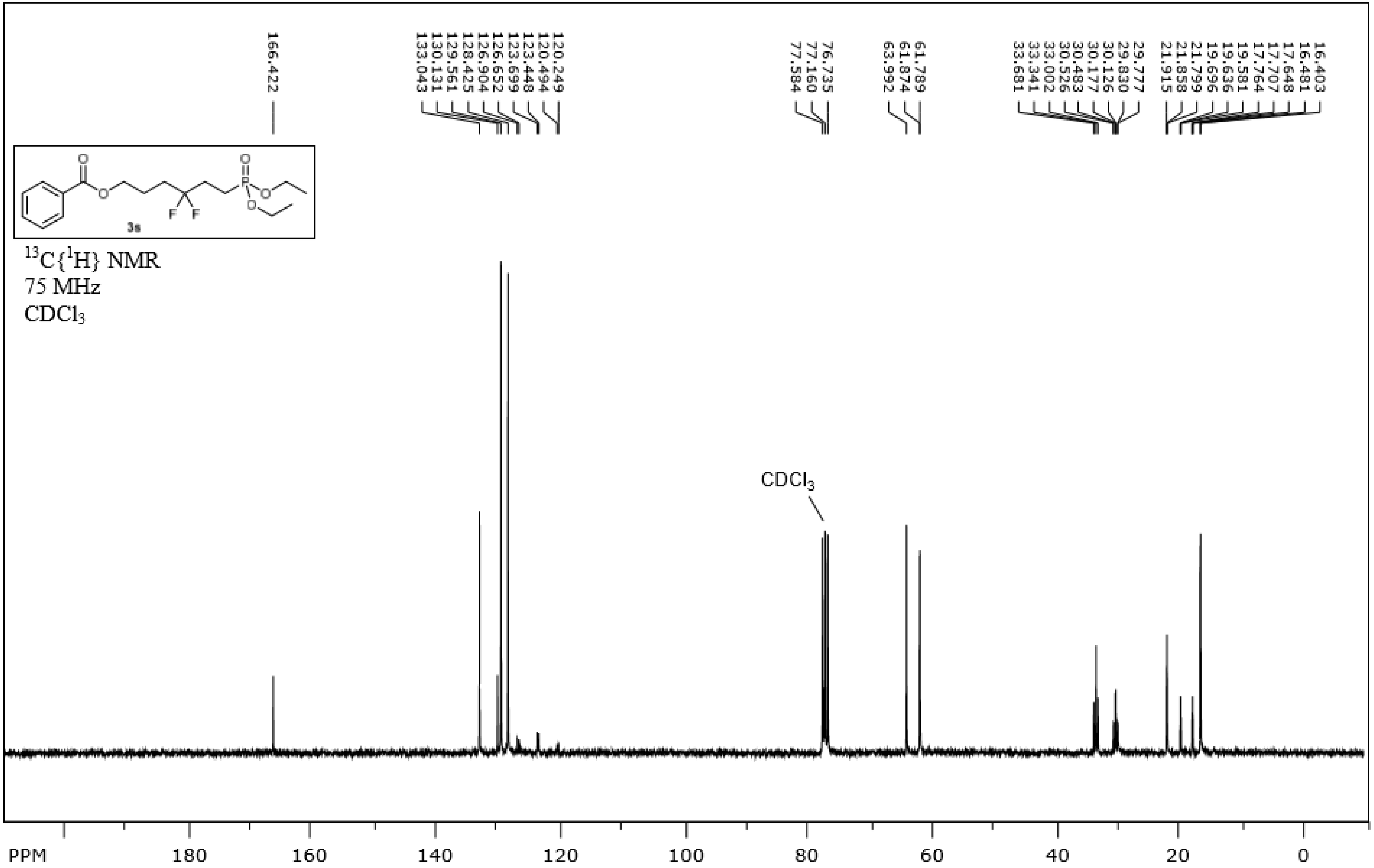
166.422
133.043
130.131
129.561
128.425
126.904
126.652
123.699
123.448
120.494
120.249
77.584
77.160
76.735
63.992
61.874
61.789
33.681
33.341
33.002
30.526
30.483
30.177
30.126
29.830
29.777
21.915
21.858
21.799
19.696
19.636
19.581
17.764
17.707
17.648
16.481
16.403
3s
$^{13}C\{^1H\}$ NMR
75 MHz
$CDCl_3$
$CDCl_3$
PPM
180
160
140
120
100
80
60
40
20
0

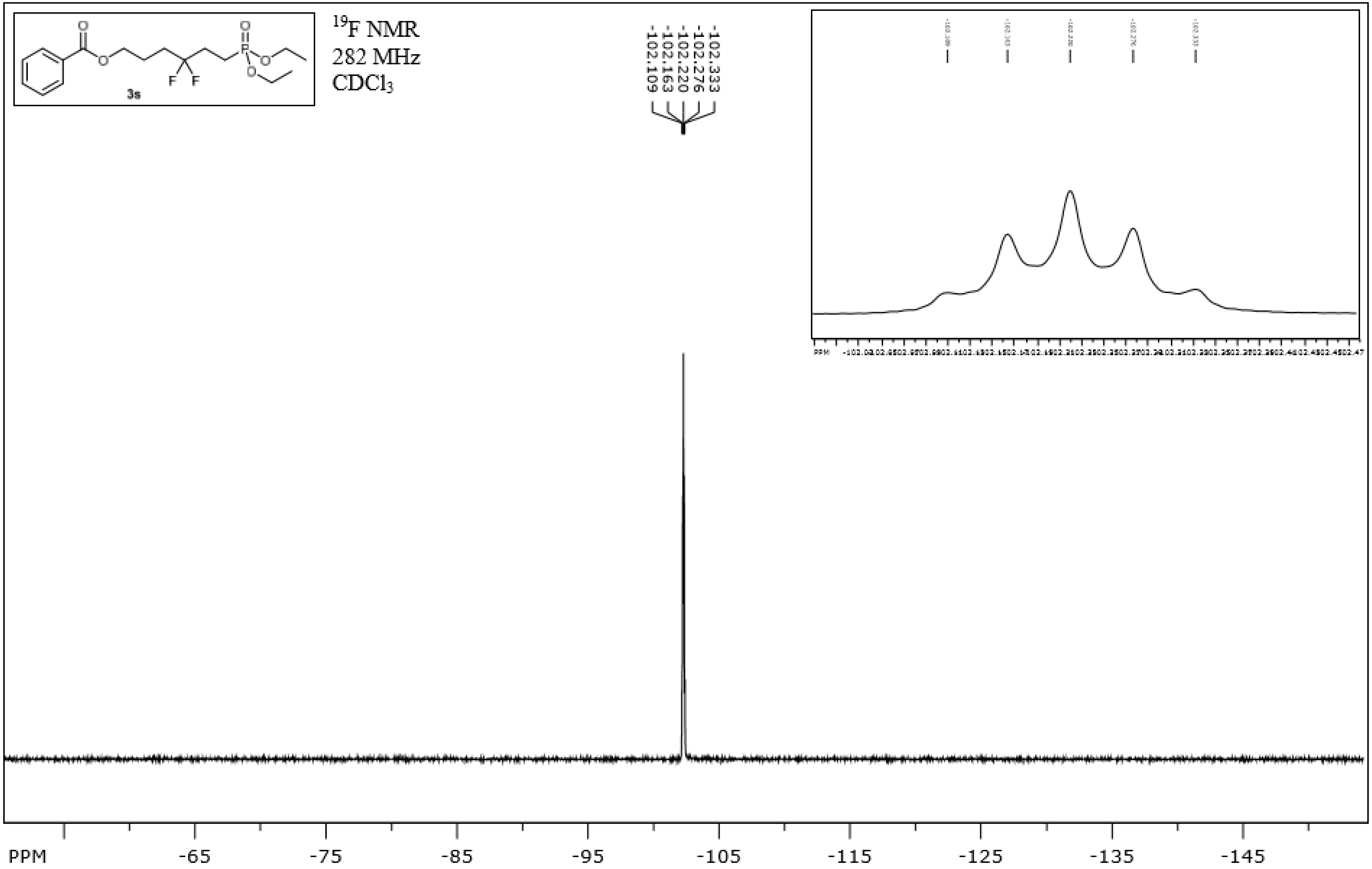

3s
19F NMR
282 MHz
CDCl3
-102.109
-102.163
-102.220
-102.276
-102.333
PPM
-65
-75
-85
-95
-105
-115
-125
-135
-145

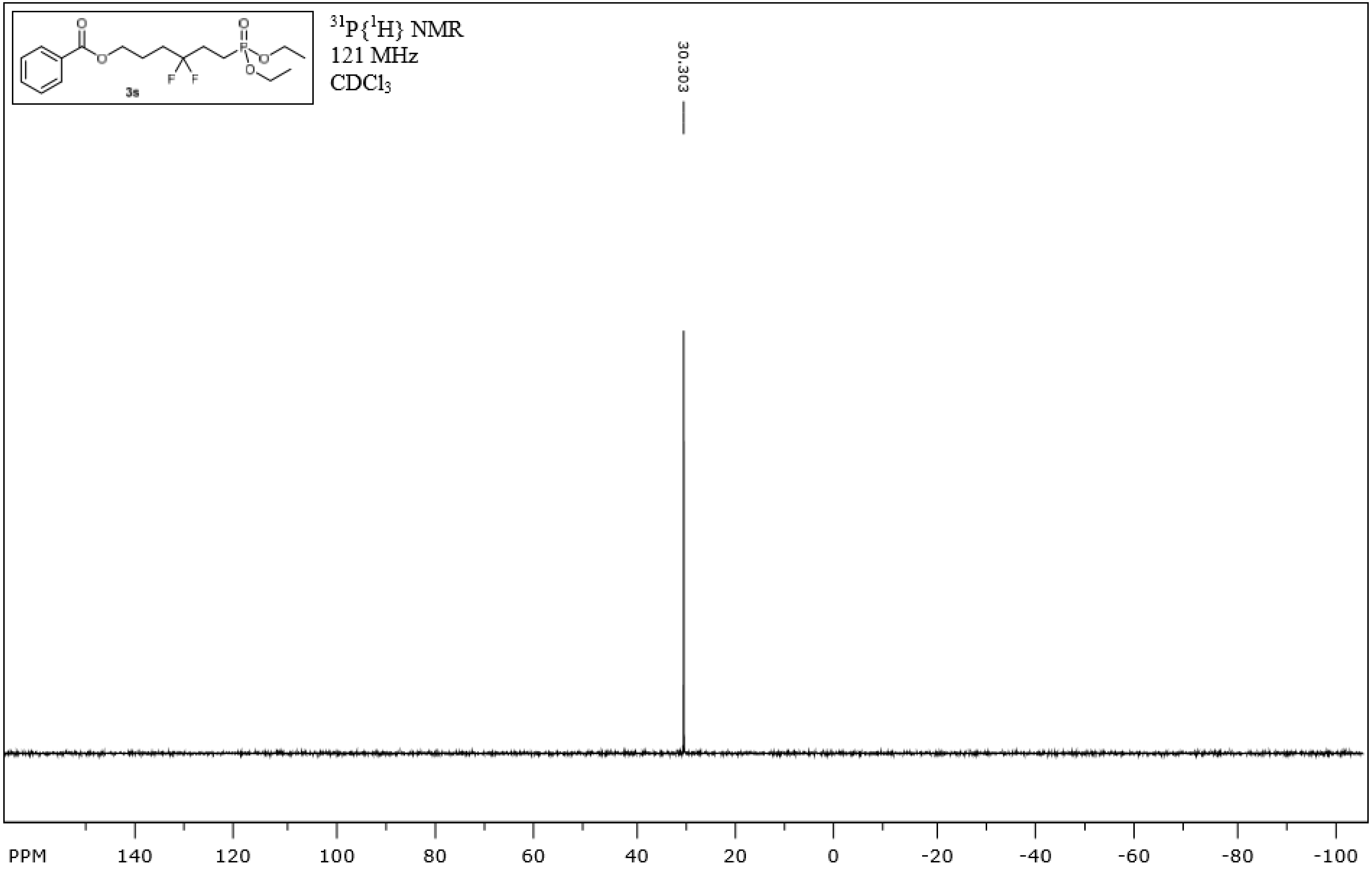

3s
$^{31}P\{^{1}H\}$ NMR
121 MHz
$CDCl_3$
30.303
PPM
140
120
100
80
60
40
20
0
-20
-40
-60
-80
-100

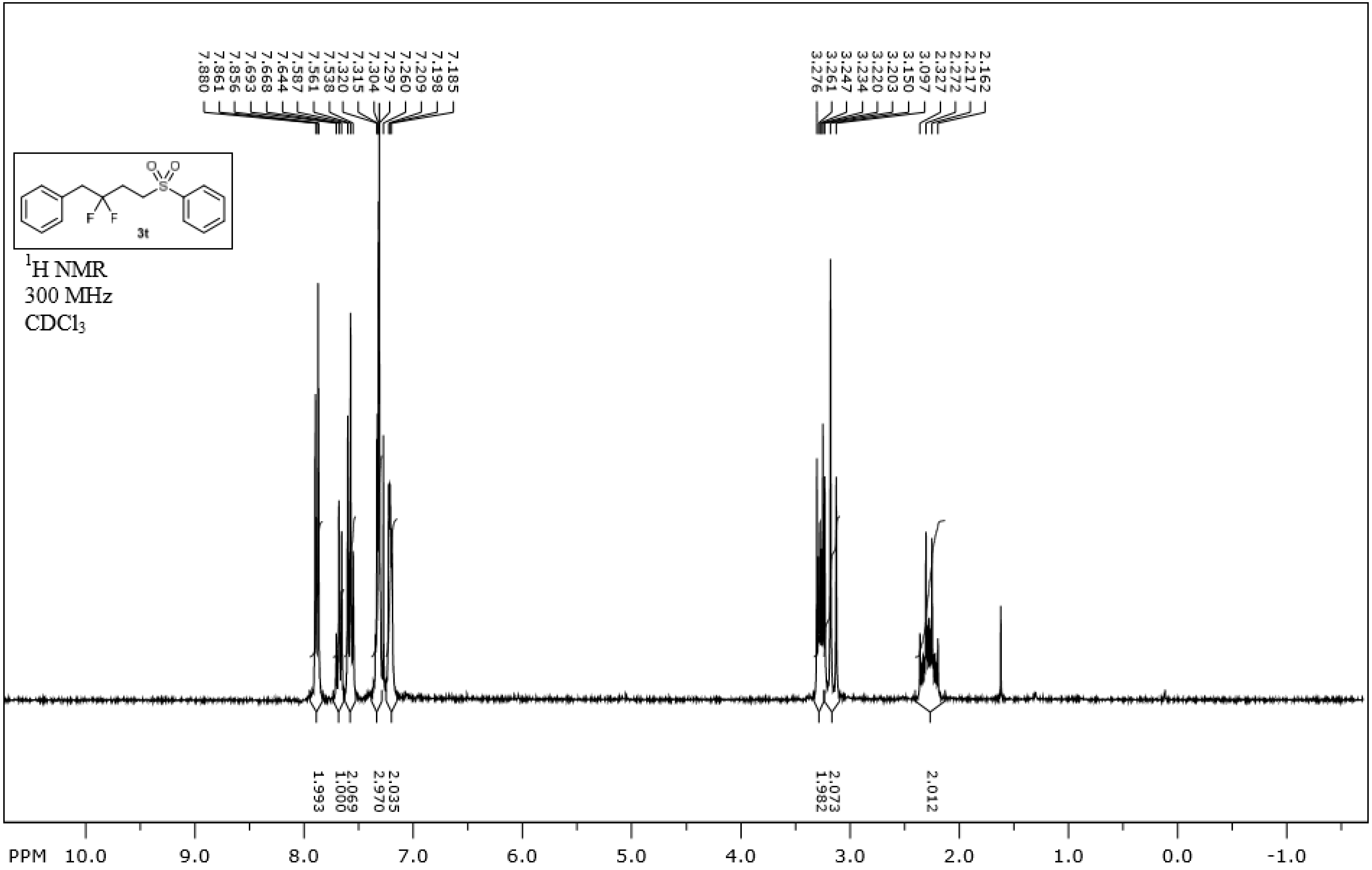
3t
^{1}H NMR
300 MHz
$CDCl_3$
7.880
7.861
7.856
7.693
7.668
7.644
7.587
7.561
7.538
7.320
7.315
7.304
7.297
7.260
7.209
7.198
7.185
3.276
3.261
3.247
3.234
3.220
3.203
3.150
3.097
2.327
2.272
2.217
2.162
1.993
1.000
2.069
2.970
2.035
1.982
2.073
2.012
PPM 10.0 9.0 8.0 7.0 6.0 5.0 4.0 3.0 2.0 1.0 0.0 -1.0

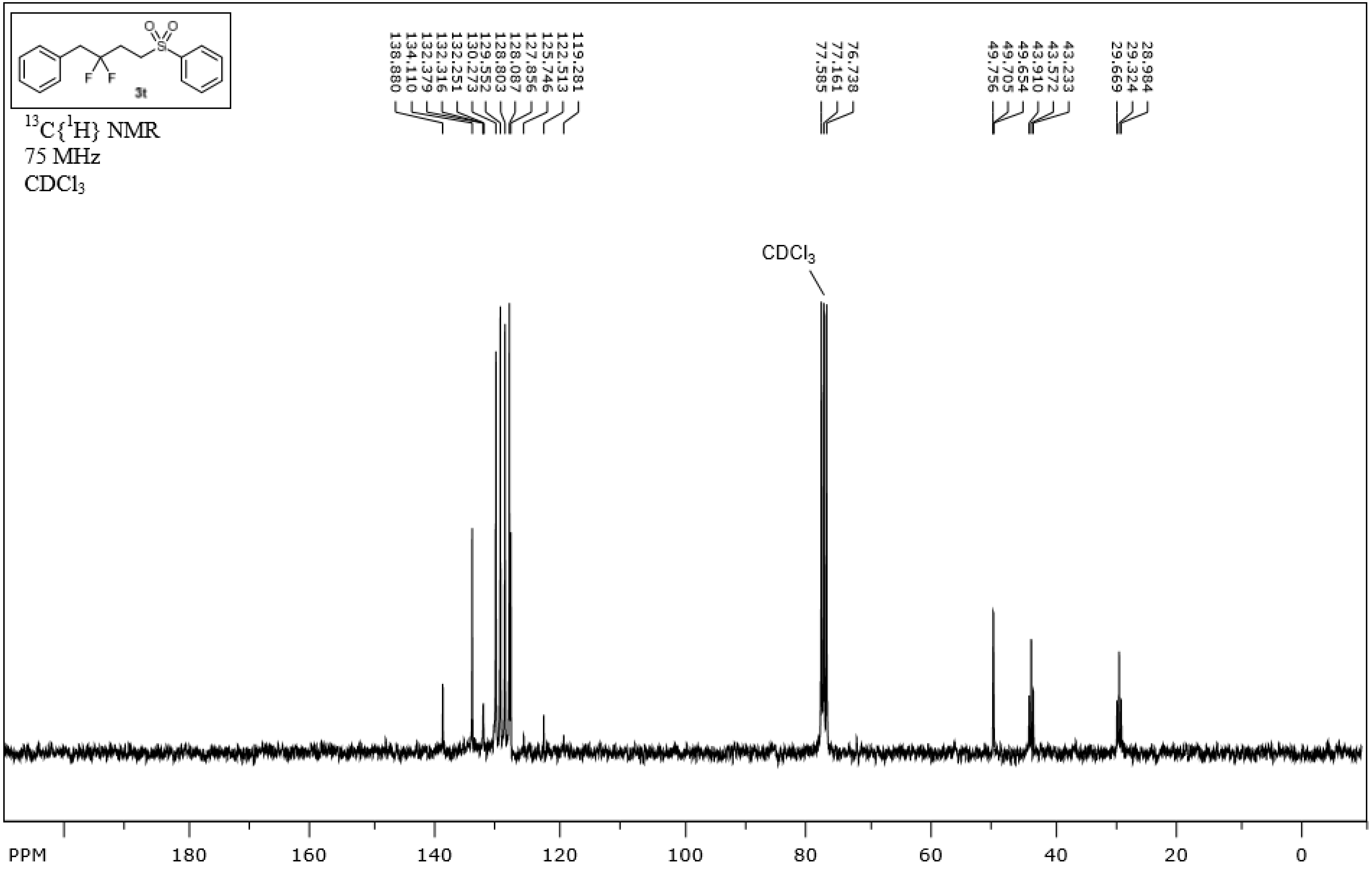
3t
$^{13}C\{^{1}H\}$ NMR
75 MHz
$CDCl_3$
138.880
134.110
132.379
132.316
132.251
130.273
129.552
128.803
128.087
127.856
125.746
122.513
119.281
77.585
77.161
76.738
49.756
49.705
49.654
43.910
43.572
43.233
29.669
29.324
28.984
$CDCl_3$
PPM
180
160
140
120
100
80
60
40
20
0

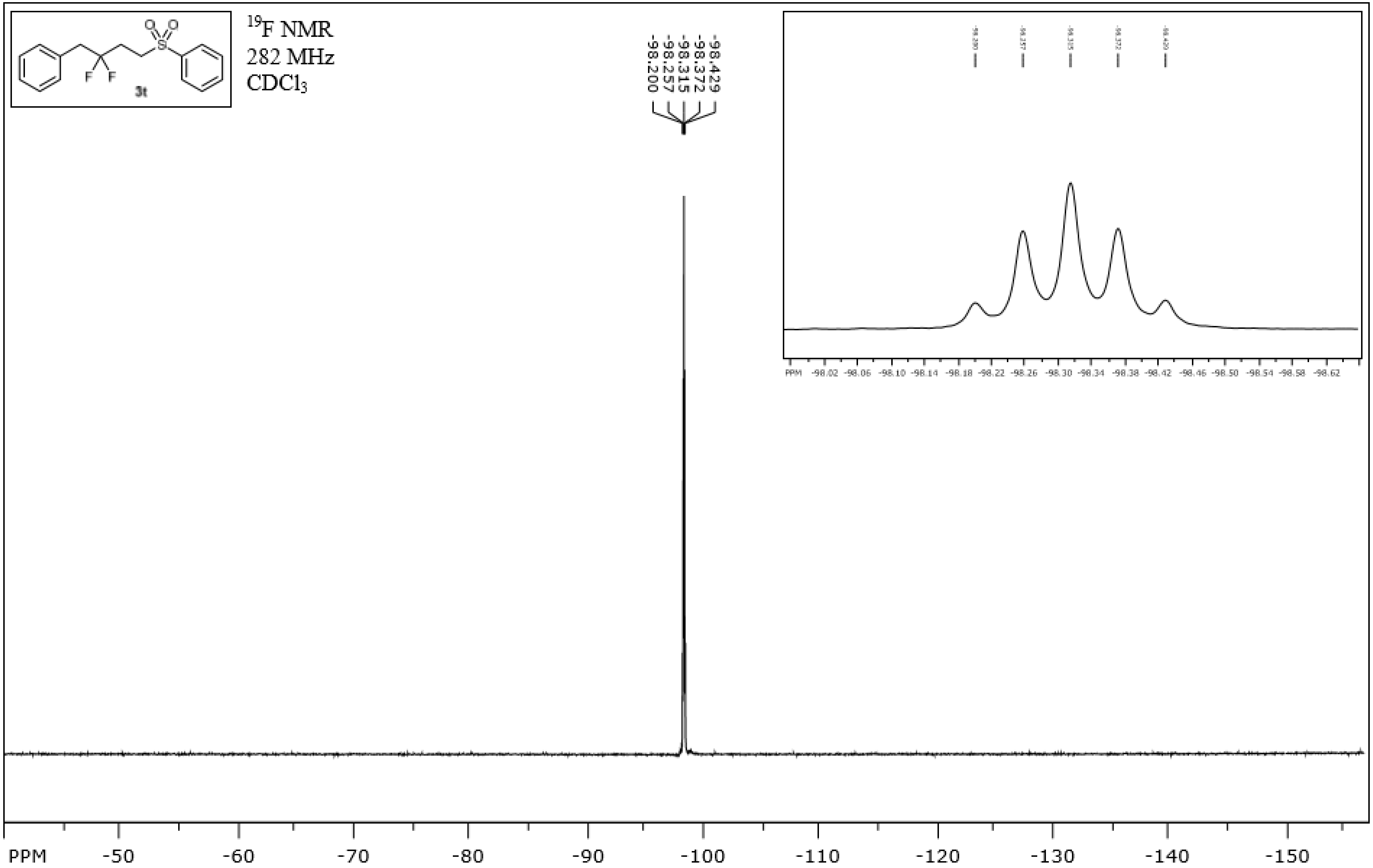

3t
^{19}F NMR
282 MHz
$CDCl_3$
-98.200
-98.257
-98.315
-98.372
-98.429
PPM -98.02 -98.06 -98.10 -98.14 -98.18 -98.22 -98.26 -98.30 -98.34 -98.38 -98.42 -98.46 -98.50 -98.54 -98.58 -98.62
PPM -50 -60 -70 -80 -90 -100 -110 -120 -130 -140 -150

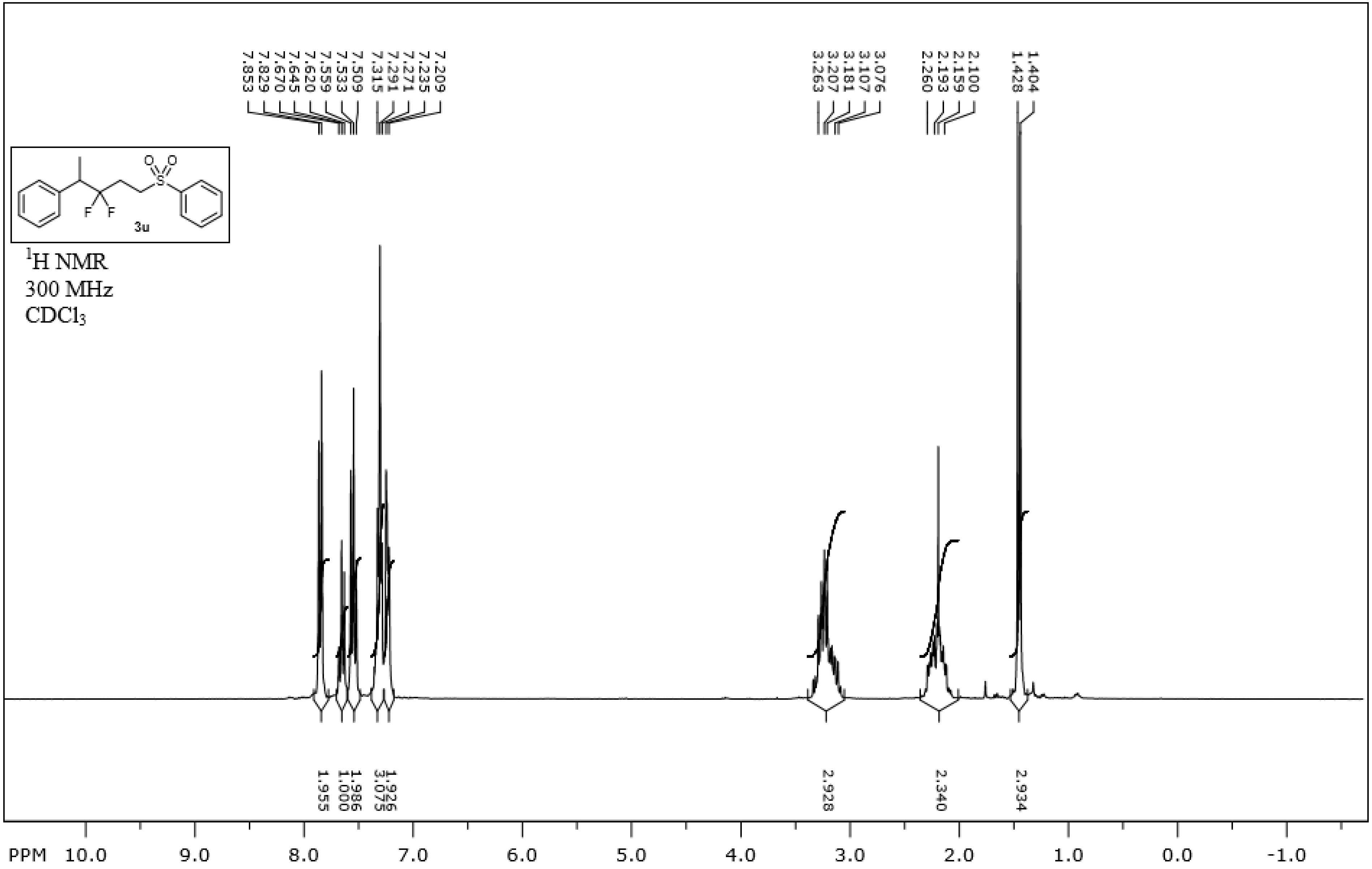

3u
1H NMR
300 MHz
CDCl3
7.853
7.829
7.670
7.645
7.620
7.559
7.533
7.509
7.315
7.291
7.271
7.235
7.209
3.263
3.207
3.181
3.107
3.076
2.260
2.193
2.159
2.100
1.428
1.404
1.955
1.000
1.986
3.075
1.926
2.928
2.340
2.934
PPM 10.0 9.0 8.0 7.0 6.0 5.0 4.0 3.0 2.0 1.0 0.0 -1.0

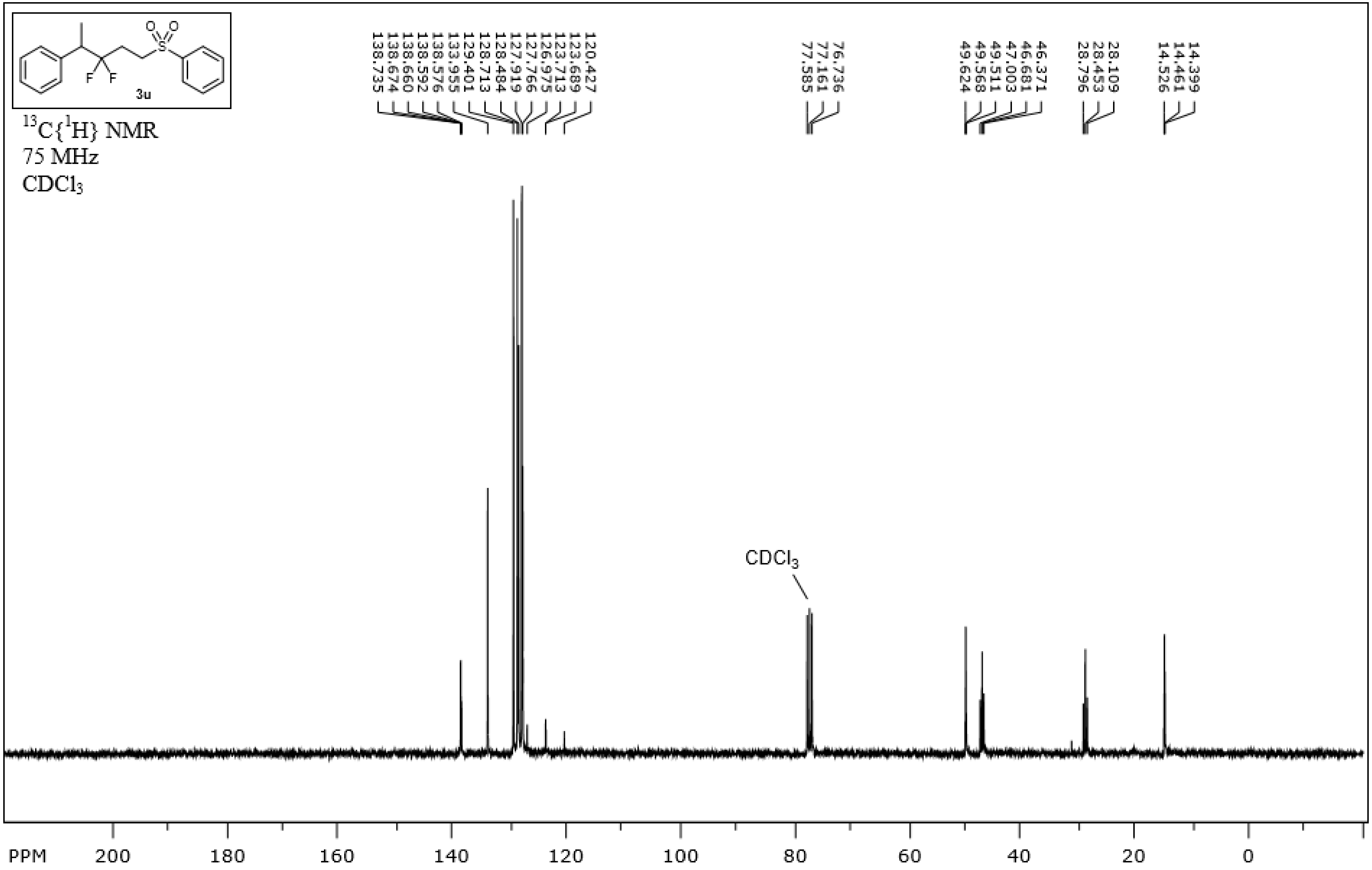
3u
$^{13}C\{^{1}H\}$ NMR
75 MHz
$CDCl_3$
138.735
138.674
138.660
138.592
138.576
133.955
129.401
128.713
128.484
127.919
127.766
126.975
123.713
123.689
120.427
77.585
77.161
76.736
49.624
49.568
49.511
47.003
46.681
46.371
28.796
28.453
28.109
14.526
14.461
14.399
$CDCl_3$
PPM
200
180
160
140
120
100
80
60
40
20
0

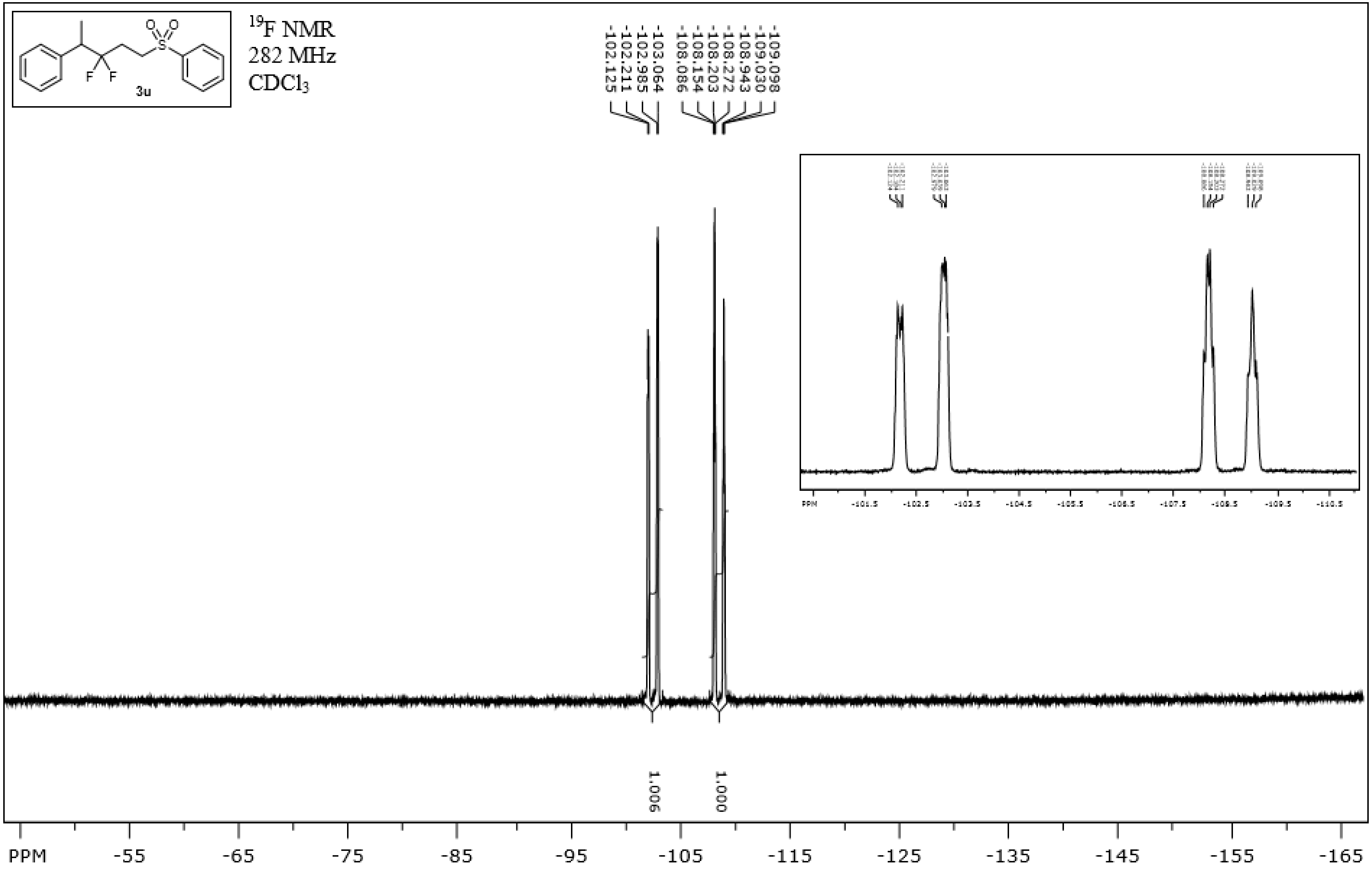

3u
19F NMR
282 MHz
CDCl3
-102.125
-102.211
-102.985
-103.064
-108.086
-108.154
-108.203
-108.272
-108.943
-109.030
-109.098
1.006
1.000
PPM
-55
-65
-75
-85
-95
-105
-115
-125
-135
-145
-155
-165
